# Investigating the mechanisms of Sini San in alleviating inflammatory responses via multi-omics and the BDNF/TrkB/PI3K/AKT signaling pathway in depressive model rats

**DOI:** 10.3389/fpsyt.2025.1628634

**Published:** 2025-10-17

**Authors:** Jia-Wei Zeng, Zhen-Jie Han, Xiu-Tang He, Xue-Jiao Liu, Hui-Yue Wang, Shu-Sheng Yang, Jing Bai, Yan-Jun Duan, Li Lin

**Affiliations:** ^1^ Department of Physiology, College of Basic Medical Sciences, Hubei University of Chinese Medicine, Hubei Shizhen Laboratory, Wuhan, China; ^2^ Department of Anatomy Teaching and Research, College of Basic Medical Sciences, Hubei University of Chinese Medicine, Wuhan, China; ^3^ Center of Monitoring and Evaluation of Teaching Quality, Jingchu University of Technology, Jingmen, China; ^4^ Department of Traditional Chinese Medicine, Wuhan Red Cross Hospital, Wuhan, China; ^5^ Department of English Major, School of Foreign Languages, Hubei University of Chinese Medicine, Wuhan, China

**Keywords:** depression, Sini San, inflammation, intestinal flora, BDNF/TrkB/PI3K/AKT signaling pathway

## Abstract

**Background:**

Sini San, from the traditional Chinese medicine classic *Treatise on Exogenous Febrile Disease*, has been reported to improve anxiety and depressive symptoms in clinical practice and exhibits certain anti-inflammatory effects. Studies have shown that the inflammatory response is not merely a concomitant feature of depression but actively contributes to its pathogenesis via neuroimmune mechanisms. However, the underlying mechanism remains unclear.

**Aim:**

This study aimed to evaluate the antidepressant effect and inflammatory profile of Sini San in chronic unpredictable mild stress (CUMS)-induced rats and to explore its potential mechanism.

**Methods:**

The primary active ingredients, targets, and pathways of Sini San in treating depression were determined through network pharmacology. The improvement of depression-like behaviors was assessed using behavioral experiments. Tissue inflammatory responses were evaluated through histopathological analysis (HE staining and Nissl staining) and quantitative measurement of inflammatory cytokines by ELISA. Western blotting (WB) was employed to quantify protein expression levels, while RT-qPCR was used to assess mRNA transcription levels. Gut microbial composition was analyzed by 16S rRNA gene amplicon sequencing, with taxonomic classification performed using the Greengenes database.

**Results:**

The data indicated that Sini San reduced inflammation related to the NLRP3 inflammasome pathway by inhibiting the expression of NLRP3, ASC, caspase-1, and downstream pro-inflammatory cytokines IL-18, IL-1β, and TNF-α. According to network pharmacology analysis, Sini San mitigated depression via modulation of the PI3K/AKT signaling pathway. Upstream and downstream proteins, including BDNF (brain-derived neurotrophic factor), TrkB (tropomyosin receptor kinase B), and p-CREB (phosphorylated cAMP response element-binding protein), which were decreased after CUMS induction, were regulated by *Sini San.* Furthermore, Sini San enhanced the expression of colonic tight junction and adhesion junction proteins ZO-1, claudin-1, and occludin-1 mRNA, while simultaneously restoring intestinal microbiota balance—indicating amelioration of CUMS-induced disruptions in intestinal barrier function and microbial composition.

**Conclusion:**

Sini San modulates the gut–brain axis by inhibiting the NLRP3 inflammasome, thereby alleviating CUMS-induced inflammation and gut microbiota dysbiosis in rats. This effect may further contribute to the improvement of depressive symptoms via regulation of the BDNF/TrkB/PI3K/AKT signaling pathway.

## Graphical Abstract

Graph abstract Schematic representation of the proposed mechanism for Sini San improving CUMS-induced depression-like behavior of rats. Our results demonstrated that CUMS-stimulated rats exhibited intestinal flora dysbiosis, which further activated the NLRP3 inflammasome pathway, leading to the release of inflammatory factors such as IL-18, IL-1β, TNF-α, etc. Inflammatory factors enter into the bloodstream and subsequently cross the blood-brain barrier (BBB), triggering neuroinflammation and synaptic damage. Furthermore, CUMS induction also affects BDNF/TrkB and its downstream PI3K/AKT signaling pathway, resulting in synaptic damage and synaptic plasticity, which ultimately contributes to depression-like behaviors. These pathological changes are reversed following treatment with Sini San. Simultaneously, our studies suggested that Sini San exerts antidepressant effects by modulating intestinal flora balance, attenuating the inflammatory response, and activating the BDNF/TrkB/PI3K/AKT signaling pathways through enhanced the phosphorylation of downstream proteins.

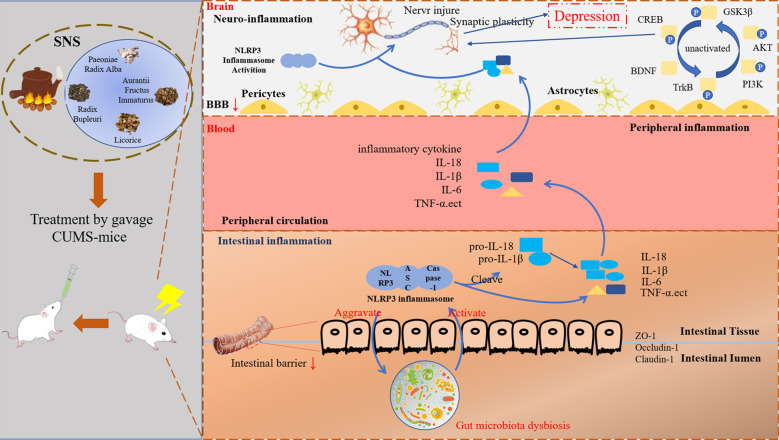

## 1 Introduction

Depression, a prevalent mental illness, manifests as persistent low mood, pervasive pessimism, cognitive slowing, sleep disturbances (insomnia/early awakening), and lethargy. According to World Health Organization (WHO) data, the global prevalence of depression is 3.8%, affecting more than 350 million people, with higher rates in adults (5.0%) and older adults (5.7%) (1, 2). Depression is further characterized by high rates of recurrence, suicide risk, and disability (3). It is estimated that approximately 800,000 people die by suicide each year, making it the second leading cause of death among individuals aged 15–29 years (4, 5). Thus, depression significantly impacts quality of life. Projections indicate that by 2030, it will become the leading contributor to the global disease burden (6).

The etiology of depression is exceedingly complex and involves multiple factors, including neurotransmitter deficiencies, dysfunctions of the hypothalamic–pituitary–adrenal axis, abnormalities in neurotrophic factors and neuroplasticity, inflammation, and imbalance of the intestinal microbiota, all of which are closely related to the onset of depression (7). Among these, the inflammation hypothesis has emerged as one of the most prominent areas of research into its pathogenesis. The inflammatory response has become central to understanding its underlying mechanisms. Furthermore, NLRP3 has been identified as a potential inflammatory marker for depression, with its activation playing a crucial role in disease development (8–11).

The intestinal microbiota is intricately linked to human health, making it a focal point of disease research. Significant differences in both diversity and homogeneity have been observed between the intestinal microbiota of patients with depression and that of healthy individuals (12). Subsequent studies revealed that certain metabolites produced by intestinal microbes can activate inflammatory cells in the circulatory system, which then migrate to the central nervous system (CNS), ultimately influencing brain function (13). Alterations in the intestinal microbiota can activate the NLRP3 inflammasome pathway, triggering inflammatory responses within the CNS and contributing to the pathogenesis of depression (14). Conversely, inflammatory responses can alter microbiota composition. Rutsch et al. (15) demonstrated that stress-induced inflammatory responses lead to gut dysbiosis and metabolite dysregulation, which further propagate neuroinflammation via the gut–brain axis, exacerbating depressive-like behaviors in mice. In summary, depression is accompanied by both inflammation and intestinal microbiota changes. The interplay between the inflammatory response and intestinal microbiota may aggravate depressive symptoms; however, the shared mechanistic pathways linking these factors in depression pathogenesis require further investigation.

Sini San, a formula from the traditional Chinese medicine classic *Treatise on Exogenous Febrile Disease* by Zhang Zhongjing, is composed of Radix Bupleuri, Paeoniae Radix Alba, Aurantii Fructus Immaturus, and *Glycyrrhizae Radix et Rhizoma* (licorice). It is traditionally used to harmonize the liver and spleen, soothe the liver, and relieve depression. Studies have shown that Sini San has antidepressant effects (16–18) and has been used clinically to treat various types of depression with notable efficacy (19–21). Recent studies further demonstrated that Sini San exerts anti-inflammatory effects by specifically inhibiting the NLRP3 inflammasome pathway, thereby alleviating systemic inflammation (22, 23). Investigations into the shared mechanisms through which Sini San modulates both anti-inflammatory processes and depressive disorders have yielded significant findings (24, 25). Based on this evidence, the present study aimed to investigate whether Sini San alleviates depression through anti-inflammatory activity and modulation of the intestinal microbiota in a chronic unpredictable mild stress (CUMS)-induced rat model.

## 2 Materials and methods

### 2.1 Network pharmacological analysis

#### 2.1.1 Depression-related target prediction

Disease-related targets with the keyword “MDD (depression)” were searched in GeneCards, Online Mendelian Inheritance in Man (OMIM), and DisGeNET. Targets from the three databases were combined, and duplicates were removed to obtain the final set of depression-related targets.

#### 2.1.2 Screening of active ingredients and targets of Sini San

The Traditional Chinese Medicine Systematic Pharmacology Database and Analysis Platform (TCMSP) was used to identify active ingredients of Radix Bupleuri, Paeoniae Radix Alba, Aurantii Fructus Immaturus, and licorice. Oral bioavailability (OB) ≥30% and drug-likeness (DL) ≥0.18 were applied as screening criteria (26). Active ingredients were further validated using the PubChem database (organic small molecule activity) and the SwissTargetPrediction database (small molecule drug target prediction). The potential targets of the active ingredients of Sini San were then retrieved.

#### 2.1.3 Prediction of potential targets of Sini San and depression

The Venny 2.1 online tool was used to map the active ingredient targets of Sini San to the disease-related targets. The intersecting targets, i.e., the potential targets of Sini San for the treatment of depression, were obtained, and a Venn diagram was generated.

#### 2.1.4 Construction of drug-active ingredient-target network

The collected drugs, their active ingredients and targets, as well as the intersecting potential targets, were imported into Cytoscape 3.9.1 to construct a “traditional Chinese medicine–active ingredient–target” network. Core ingredients were analyzed, and the degree value of each active ingredient was obtained through topological analysis.

#### 2.1.5 Screening core targets

The intersecting targets were imported into the STRING database, with “Homo sapiens” set as the species. A protein–protein interaction (PPI) network was constructed using a confidence score ≥0.9 (27). The resulting TSV files were imported into Cytoscape 3.9.1, and PPI network topology was analyzed using the CentiScaPe 2.2 plug-in. Core targets of Sini San in depression were obtained by screening based on topological parameters, including closeness centrality, betweenness centrality, and degree centrality.

#### 2.1.6 GO, KEGG enrichment analysis

Gene Ontology (GO) and Kyoto Encyclopedia of Genes and Genomes (KEGG) pathway enrichment analyses were performed on the screened core targets using the Database for Annotation and Visual of Biological Information (DAVID, http://david.ncifcrf.gov/tools.jsp), to obtain the biological process (BP), cellular components (CC), molecular functions (MF), and signaling pathways relevant to Sini San in depression treatment.

#### 2.1.7 Molecular docking verification

The top three active ingredients in the “TCM–active ingredient–target” network and the top three target proteins (core targets) and pathway-related proteins in the PPI network were selected for molecular docking. The 2D structure of the active ingredients and the 3D structure of the target proteins were downloaded from the PubChem and PDB databases, respectively. AutoDock Vina software was used for molecular docking, and hydrogen bond sites and binding energies of the active ingredients and target protein receptors were obtained. Binding energy less than 0 and < −5 kcal/mol indicated that the two molecules could bind spontaneously, with smaller binding constants reflecting tighter interactions (28). The RMSD < 2.0 Å, based on the root mean square deviation of multiple poses output by Vina, indicated that the self-detection of the docking process met the standard. Re-docking was performed to verify that the RMSD remained < 2.0 Å. Finally, PyMOL was used for visual analysis to confirm the rationality of the interactions.

### 2.2 Animal experiments

#### 2.2.1 Animals

Fifty SPF-grade male SD rats weighing 300 ± 10 g (10–12 weeks old) were purchased from Liaoning Changsheng Biotechnology Co., Ltd. (license No. SCXK [Liao] 2020-0001). Rats were housed in the animal center under controlled conditions: temperature 18–26°C, humidity 40%–70%, adequate ventilation, and a 12-h light/dark cycle with lights on at 08:00 daily. All animals underwent adaptive feeding for 1 week.

#### 2.2.2 Drugs

Sini San was purchased from Guoyitang, Hubei University of Chinese Medicine. The formulation consisted of Radix Bupleuri, Paeoniae Radix Alba, Aurantii Fructus Immaturus, and licorice, 6 g each, decocted into a medicinal soup. Based on the dose conversion ratio between humans and rats, the low concentration of *Sini San* was calculated as 24g/60kg*6.3 = 0.252g/100g, referring to the clinical dosage of Sini San and the ninth edition of *Pharmacology*) that the low concentration of Sini San was 0.25g/100g and the high concentration of 0.5g/100g, the final concentration of the soup was 1g/ml and 2g/ml, and the western reference drug escitalopram oxalate (H. Lundbeck.A/S) was dissolved in saline (administered at a concentration of 0.105mg/100g, as above) to make a suspension of 0.45mg/ml.

#### 2.2.3 Animal grouping and treatment

Fifty rats were randomly divided into five groups (n = 10 per group): normal control (CON), model (MOD), low-dose Sini San (SNS-L), high-dose Sini San (SNS-H), and positive control receiving escitalopram oxalate tablets (EOT). Except for the CON group, all rats were subjected to the CUMS procedure for 42 days before drug administration. Saline was given to the CON and MOD groups. SNS-L and SNS-H received low- and high-dose *Sini San*, respectively, and EOT received escitalopram oxalate. All medications were administered by gavage at 0.75 mL/day for 21 days.

#### 2.2.4 CUMS procedure

Except for the CON group, the other four groups were exposed to the following stressors: ①Food deprivation for 24h. ②Water deprivation for 24h. ③Cage tilted at 45° for 12h. ④Shaking the cage for 1.5h. ⑤Electric shock to the foot, 1mA for 10s, 10s intervals, total of 2min. ⑥Damping of bedding for 24h with water just enough to soak the bedding. ⑦Tail suspension for 5min. ⑧Tail clamping for 2 min. ⑨Cold water bath at 4°C for 10 min. ⑩Empty cage for 24 h. Stressors were applied randomly, with no repetition on two consecutive days (29–32).

#### 2.2.5 Behavioral tests

##### 2.2.5.1 Sucrose preference test

The SPT is a widely used method to assess anhedonia, a core symptom of depression (33). The protocol was divided into an adaptation period of 3 days and an experimental period of 1 day. On day 1, two bottles of 1% sucrose solution were given to each rat. On day 2, one bottle was replaced with pure water. On day 3, food and water were withheld. On day 4, each rat was given 100 mL of pure water and 100 mL of 1% sucrose solution, and the remaining amounts were weighed after 24 h. The sucrose preference of each rat was calculated as follows:

(Sucrose consumption/Sucrose consumption + Pure water consumption×100%) (34, 35).

##### 2.2.5.2 Forced swimming test

The FST induces a state of behavioral despair in rodents and is used to evaluate depressive-like behavior (36). The protocol was adapted from previous studies with minor modifications (37, 38). Rats were individually placed in a glass cylinder (height: 1 m, diameter: 30 cm) filled to 70 cm with water (23 ± 1°C). Each rat was subjected to a 6-min session. The first 2 min were considered acclimatization, and immobility time during the final 4 min was recorded using EthoVision XT 9 software. Immobility was defined as the absence of struggling, twisting, or bending movements (37).

##### 2.2.5.3 Open field test

The OFT was used to evaluate locomotor activity and anxiety-like behavior (38, 39). The apparatus consisted of a black wooden box (1 × 1 m²) virtually divided into 16 equal squares. At the start of each test, the rat was gently placed in the center of the arena and allowed to explore freely for 6 min. The first minute was considered habituation, and activity during the subsequent 5 min was analyzed using EthoVision XT 9 software to measure total distance moved and average speed. After each test, the arena was cleaned of fecal residues and wiped with 75% ethanol to eliminate olfactory cues (16, 40).

##### 2.2.5.4 Novelty food suppression feeding

The NSF assesses depression and anxiety by measuring latency to feed in a novel environment (41). The experimental setup was identical to that of the OFT, with minor modifications. Rats were food-deprived for 24 h before the test, with free access to water. On the test day, a known weight of high-fat chow (distinct from standard chow) was placed in the center of the wooden box. Rats were placed at the edge of the box and observed for 5 min using EthoVision XT 9.0 software to record latency to feed (time from entry into the new environment until feeding). Remaining food was weighed to calculate consumption. The arena was cleaned with 75% ethanol after each trial to remove residual odors (42).

##### 2.2.5.5 Morris water maze

This experiment was used to assess spatial learning and memory by measuring the number of times rats crossed the platform area and the latency to reach the platform (43). A circular pool (diameter 150 cm) was divided into four quadrants, with a submerged hidden platform fixed in one quadrant. The experiment lasted 7 days. For the first 6 days, rats were trained by being released from different quadrants. Each trial lasted 60 s, during which rats could search for the platform. Rats that successfully located the platform were allowed to rest on it. Rats that did not find the platform within 60 s were manually guided to it and kept there for 30 s. On day 7, the platform was removed, and a probe trial was conducted to assess spatial memory (44).

#### 2.2.6 Tissue sampling

On the day following behavioral testing, all rats were euthanized for sample collection. Under anesthesia with 1% sodium pentobarbital (50 mg/kg), rats were divided into two cohorts. In one cohort, animals were perfused with paraformaldehyde, and whole brain and colon tissues were collected and preserved in fixative. In the other cohort, blood was collected from the abdominal aorta, followed by decapitation to obtain hippocampal and cortical tissues. Colon and cecal contents were also collected. Blood samples were left at room temperature for 30 min, then centrifuged at 4 °C, 3,000 rpm for 15 min to obtain serum. Serum samples were frozen in liquid nitrogen and stored at −80 °C.

##### 2.2.6.1 HE staining

Whole brain and colon tissues were fixed in 4% paraformaldehyde for 48 h, dehydrated, embedded in paraffin, cut into 5-μm transverse sections, and stained with hematoxylin-eosin. Sections were analyzed by light microscopy.

##### 2.2.6.2 Nissl staining

Coronal and transverse brain sections (3–4 mm) were cut and placed in embedding frames. Tissues underwent gradient dehydration and paraffin embedding. Sections 4 μm thick were cut, mounted onto adhesive slides, and baked at 70 °C for 2 h. After deparaffinization, Nissl staining solution was prepared by mixing toluidine blue master solution (1 g toluidine blue in 100 mL anhydrous ethanol) with 0.1% NaCl solution in a 1:1 ratio. Staining was performed according to standard protocols. Images were captured under a microscope and analyzed.

##### 2.2.6.3 ELISA

Levels of IL-18 (RX302871R), IL-1β (RX302869R), and TNF-α (RX302058R) in serum, colon, and hippocampal tissues were measured using commercial ELISA kits (RuiXin Biotech, Guangzhou, China). All measurements were performed in triplicate following the manufacturer’s instructions. Absorbance at 450 nm was measured using a CLARIOstar Plus microplate reader (BMG LABTECH, Germany). Cytokine concentrations were determined from standard curves for each cytokine.

##### 2.2.6.4 Western blot

Hippocampal tissues were homogenized in RIPA lysate (G2002-100ML, Servicebio) supplemented with phosphatase inhibitor (G2007-1ML, Servicebio) and 1 mM PMSF (G2008-1ML, Servicebio) using a bead mill (60 Hz, 120 s × 3 cycles). The lysates were centrifuged at 12,000 rpm for 15 min at 4°C. The protein supernatant was collected, concentration determined by BCA protein assay kit, and proteins denatured with 5× buffer by boiling. Each protein sample was separated by SDS-PAGE and transferred to a PVDF membrane, blocked with 5% skimmed milk at room temperature for 1 h, then washed with TBST (10 min × 3 cycles). Primary antibodies were incubated overnight at 4°C (NLRP3, 68102-1-Ig. ASC, 67494-1-Ig. Caspase-1, 22915-1-AP. IL-18, 60070-1-Ig. IL-1β, 16806-1-AP. TNF-α, 60291-1-Ig. all Proteintech) (BDNF, bs-4989R, bioss. Trkb, WL00839, Wanleibio. CREB, A10826, p-CREB, AP0019, PI3K, A0265, P13K-p110α, A22730, Abclonal. AKT, 9272S, p-AKT, 4060S, CST. GSK3β, 67329-1-Ig, p-GSK3β, 67558-1-Ig, Proteintech). After washing the membranes (ibid), goat anti-rabbit or goat anti-mouse IgG enzyme-labelled antibodies (HRP-Goat Anti-Rabbit, RGAR001, HRP-Goat Anti-Mouse, RGAM001, Proteintech) were incubated for 2 h at room temperature. Protein bands were visualized using ECL and analyzed with ImageJ software, normalized to GAPDH.

##### 2.2.6.5 Real-Time Quantitative Reverse Transcription PCR

Referring to previous methods with appropriate modifications (45, 46), total RNA was extracted from isolated hippocampus and colon using QIAzol lysis reagent and purified with HiScript II Q RT SuperMix for qPCR (+gDNA wiper) (Vazyme Biotech Co., Ltd). cDNA was synthesized from total RNA with a high-capacity reverse transcription kit (Life Technologies). cDNA was then amplified using Taq Pro Universal SYBR qPCR Master Mix (Vazyme Biotech Co., Ltd), noted for good specificity, high sensitivity, and high amplification yield. qPCR was performed using the CFX96 real-time system (Bio-Rad, USA). Relative quantification was carried out using the ΔΔCT method. Primers were designed and synthesized by Beijing Kengke Biotechnology Co., Ltd (**Table 1**).

**Table 1:** Sequence of primers.

Primers	Forward primer	Reverse primer
ZO-1	AACCCGAAACTGATGCTGTG	CCCTTGGAATGTATGTGGAGAG
Claudin-1	GGAGTCAGTGTTTCAGCCTATGGT	GAAGGGTTCATGCCTCTCATCT
Occludin-1	CCCAGATTAGAGTCCAAAGTCAGT	CGGAAACCTTAGAGAGATGCC
GAPDH	AAGAAGGTGGTGAAGCAGG	GAAGGTGGAAGAGTGGGAGT
BDNF	AGCTTGTATCCGACCCTCTCTG	CAGCAATCAGTTTGTTCGGC
Trkb	GGTGGCTGTGAAGACGCTGAAG	AATGTGCTCGTGCTGGAGGTTG
CREB	CTGATTCCCAAAAACGAA	CTGCCCACTGCTAGTTTGGT
GAPDH	CCATGTTTGTGATGGGTGTG	CCTTCCACAATGCCAAAGTT

##### 2.2.6.6 16s RNA assay

Total DNA was extracted from cecum content samples using a standardized protocol, and microbial composition was analyzed by 16S rRNA sequencing. All procedures were performed by Shanghai Sangon Biotechnology Co., Ltd (Shanghai, China). To analyze taxonomic composition, the V3–V4 hypervariable region of the 16S rRNA gene was selected for pyrosequencing. The V3–V4 region was amplified by PCR using primers 341F (CCCTACACACGACGCTCTTCCGATCTG) and 805R (GACTGGAGTTCCTTGGCACCCGAGAATTCCA). Sequencing results were analyzed on the Paisano Gene Cloud Platform (Paisano, Shanghai, China) according to standard instructions.

### 2.3 Statistical analysis

All data were expressed as mean ± standard deviation and statistically analyzed using SPSS Statistics 26.0 software. Statistical differences were compared using one-way ANOVA, and p < 0.05 was considered statistically significant.

## 3 Results

### 3.1 Identification of the core targets and pathways of Sini San in depression treatment via network pharmacology

**Figure 1:**
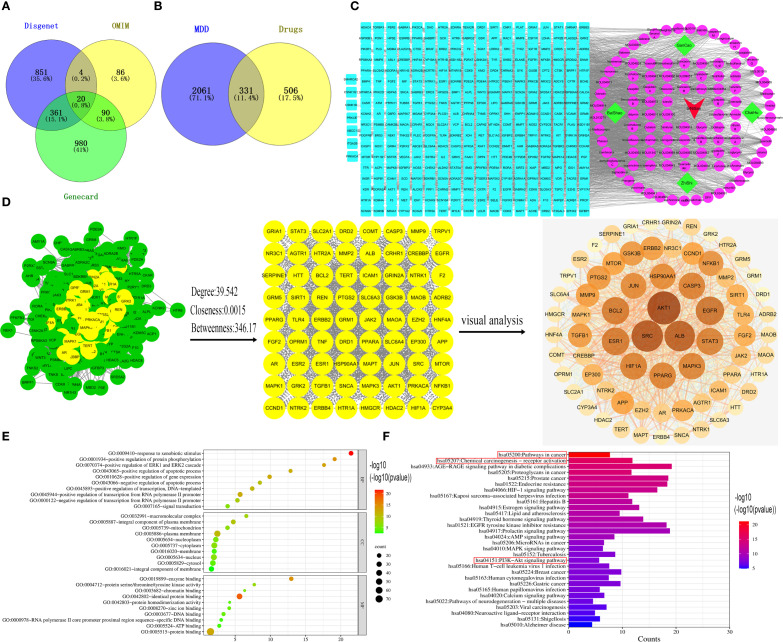
Network pharmacological analysis of the treatment of depression by Sini San. **(A)** Venn diagram of depression-related targets, querying depression targets from the Gene Card, OMIM and Disgent databases and taking their intersection. **(B)** We identified the relevant targets for Sini San from the TCMSP, Pubchem and Swiss Target databases, then by intersecting these with the targets related to depression, we obtained potential targets for Sini San in treating depression. **(C)** Drug-active ingredient-target network diagram. using Cytoscpe 3.9.1 software to analyze traditional Chinese medicines, the corresponding active ingredients and related targets, to obtain their topological analysis diagrams. **(D)** Core target PPI network of Sini San in treating depression, put potential target into the String database to get the initial PPI, and then use CytoNCA to analyze and identify the core targets. **(E)** GO enrichment analysis, the core targets were analyzed by GO and the top 10 highest correlations to the targets were taken separately. **(F)** KEGG enrichment analysis, the core targets were analyzed by KEGG and the top 30 pathways with the highest correlation to the targets were taken.

GeneCards returned 1,451 related targets, OMIM 200, and DisGeNET 1,236. After merging and removing duplicates, 2,392 depression-related targets were obtained (**Figure 1A**). Through the TCMSP database, the active ingredients of Sini San were identified according to OB and DL conditions: 13 from Radix Bupleuri, 92 from licorice, 13 from Paeoniae Radix Alba, and 22 from Aurantii Fructus Immaturus, totaling 144 (**Table 2** lists the top 30 by bioavailability). From PubChem and SwissTarget, 837 predicted targets were obtained, and the intersection with disease-related targets yielded 331 depression-related targets of Sini San (**Figure 1B**). In Cytoscape 3.9.1, a “traditional Chinese medicine–active ingredient–target” network was constructed, and topological analysis identified the 10 active ingredients with the highest degree values (**Table 3**, **Figure 1C**). The PPI network from the STRING database was analyzed using the CentiScaPe 2.2 plug-in in Cytoscape 3.9.1. Based on topological parameters (degree = 39.542, closeness = 0.00150, betweenness = 364.170), 72 core targets of Sini San in depression were identified (**Figure 1D**; **Table 4** lists the top 10). GO enrichment analysis revealed 785 terms: 589 molecular functions (mainly protein binding, enzyme binding, DNA binding, ATP binding, zinc ion binding), 83 cellular components (macromolecular complexes, integral membrane proteins, mitochondria, plasma membrane, nucleoplasm), and 113 biological processes (signal transduction, regulation of RNA polymerase II promoter transcription, response to external stimuli, promotion of apoptosis) (**Figure 1E**). KEGG pathway analysis identified 160 significantly enriched pathways, including cancer pathways, chemical carcinogenesis–receptor activation, cardiovascular disease, Kaposi sarcoma–associated herpesvirus infection, lipid and atherosclerosis, PI3K–AKT signaling, AGE–RAGE signaling in diabetic complications, MAPK signaling, prostate cancer, and HIF-1 signaling (**Figure 1F**).

**Table 2:** Top 30 for bioavailability.

Number	Mol ID	Compound name	OB%	DL	Source
1	MOL002311	Glycyrol	90.78	0.67	Glycyrrhiza uralensis Fisch.
2	MOL001918	Paeoniflorgenone	87.59	0.37	Cynanchum otophyllum Schneid
3	MOL004990	7,2',4'-trihydroxy-5-methoxy-3-arylcoumarin	83.71	0.27	Glycyrrhiza uralensis Fisch.
4	MOL004904	Licopyranocoumarin	80.36	0.65	Glycyrrhiza uralensis Fisch.
5	MOL004891	shinpterocarpin	80.3	0.73	Glycyrrhiza uralensis Fisch.
6	MOL004644	Sainfuran	79.91	0.23	Radix Bupleuri
7	MOL005017	Phaseol	78.77	0.58	Glycyrrhiza uralensis Fisch.
8	MOL004841	Licochalcone B	76.76	0.19	Glycyrrhiza uralensis Fisch.
9	MOL004810	glyasperin F	75.84	0.54	Glycyrrhiza uralensis Fisch.
10	MOL001484	Inermine	75.18	0.54	Glycyrrhiza uralensis Fisch.
11	MOL000500	Vestitol	74.66	0.21	Glycyrrhiza uralensis Fisch.
12	MOL005007	Glyasperins M	72.67	0.59	Glycyrrhiza uralensis Fisch.
13	MOL013433	prangenin hydrate	72.63	0.29	Aurantii Fructus Immaturus
14	MOL001798	neohesperidin_qt	71.17	0.27	Aurantii Fructus Immaturus
15	MOL004941	(2R)-7-hydroxy-2-(4-hydroxyphenyl)chroman-4-one	71.12	0.18	Glycyrrhiza uralensis Fisch.
16	MOL004959	1-Methoxyphaseollidin	69.98	0.64	Glycyrrhiza uralensis Fisch.
17	MOL000392	formononetin	69.67	0.21	Glycyrrhiza uralensis Fisch.
18	MOL001925	paeoniflorin_qt	68.18	0.4	Cynanchum otophyllum Schneid
19	MOL001928	albiflorin_qt	66.64	0.33	Cynanchum otophyllum Schneid
20	MOL004863	3-(3,4-dihydroxyphenyl)-5,7-dihydroxy-8-(3-methylbut-2-enyl)chromone	66.37	0.41	Glycyrrhiza uralensis Fisch.
21	MOL004903	liquiritin	65.69	0.74	Glycyrrhiza uralensis Fisch.
22	MOL004808	glyasperin B	65.22	0.44	Glycyrrhiza uralensis Fisch.
23	MOL001910	11alpha,12alpha-epoxy-3beta-23-dihydroxy-30-norolean-20-en-28,12beta-olide	64.77	0.38	Cynanchum otophyllum Schneid
24	MOL004829	Glepidotin B	64.46	0.34	Glycyrrhiza uralensis Fisch.
25	MOL013435	poncimarin	63.62	0.35	Aurantii Fructus Immaturus
26	MOL004855	Licoricone	63.58	0.47	Glycyrrhiza uralensis Fisch.
27	MOL013436	isoponcimarin	63.28	0.31	Aurantii Fructus Immaturus
28	MOL004914	1,3-dihydroxy-8,9-dimethoxy-6-benzofurano(3,2-c)chromenone	62.9	0.53	Glycyrrhiza uralensis Fisch.
29	MOL005828	nobiletin	61.67	0.52	Aurantii Fructus Immaturus
30	MOL004835	Glypallichalcone	61.6	0.19	Glycyrrhiza uralensis Fisch.

**Table 3:** Top 10 active ingredients.

Number	Mol ID	Compound name	Degree
1	MOL000422	kaempferol	126
2	MOL004328	naringenin	88
3	MOL000098	quercetin	78
4	MOL000354	isorhamnetin	78
5	MOL004905	3,22-Dihydroxy-11-oxo-delta(12)-oleanene-27-alpha-methoxycarbonyl-29-oic acid	55
6	MOL004991	7-Acetoxy-2-methylisoflavone	54
7	MOL004653	(+)-Anomalin	53
8	MOL004908	Glabridin	51
9	MOL013279	5,7,4'-Trimethylapigenin	49
10	MOL013277	Sinensetin	49

**Table 4:** Top 10 core targets for score.

Number	Gene name	Betweenness	Closeness	Degree
1	AKT1	5477.587	0.00211	180
2	SRC	9637.33	0.002041	167
3	ALB	4605.429	0.002024	160
4	EGFR	2251.665	0.001961	144
5	ESR1	3248.132	0.001927	138
6	BCL2	1446.834	0.001923	136
7	STAT3	1631.776	0.001916	133
8	HIF1A	1893.308	0.001898	132
9	CASP3	1155.049	0.001905	131
10	MAPK3	1793.716	0.001905	130

### 3.2 Molecular docking of core pathway proteins, core targets and active ingredients

**Figure 2:**
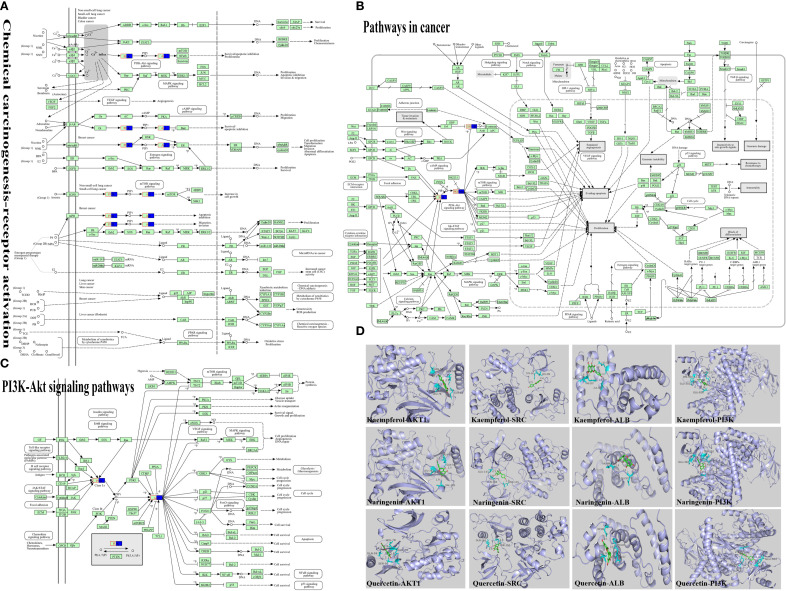
Molecular docking of active ingredients to relevant targets. **(A)** Chemical carcinogenesis-receptor activation, the pathways with the first score value obtained by KEGG enrichment analysis were visualized. **(B)** Pathway in cancer, the pathways with the second score value obtained by KEGG enrichment analysis were visualized. **(C)** PI3K-Akt signaling pathway, from the two highest-scoring pathways, we identified the key pathway as the PI3K/AKT signaling pathway. **(D)** Visualisation of molecular docking of key active ingredients to core targets, we identified the key targets as PI3K and AKT from the visualization analysis of the PI3K/AKT signaling pathway, perform molecular docking analysis using the main active compounds, core targets, and pathway proteins obtained from the analysis.

KEGG analysis indicated that cancer-related and chemical carcinogenesis–receptor activation pathways had the highest enrichment scores, suggesting a close association with depression. The PI3K/AKT signaling pathway was visualized as a key pathway (**Figures 2A–C**). Active ingredients kaempferol, naringenin, and quercetin were docked with core targets (AKT1, SRC, ALB) and pathway protein PI3K using AutoDock Vina software (**Figure 2D**). The binding energies (**Table 5**) were all ≤ −5 kcal/mol, with the lowest between naringenin and AKT1 (−6.97 kcal/mol), indicating the strongest binding.

**Table 5:** Binding energies.

Compounds PDB ID	Docking score (-kcal/mol)			
	AKT1	SRC	ALB	PI3K
	7NH5	2SRC	1YSX	5JHB
Kaempferol	6.61	5.21	6.22	6.14
Naringenin	6.97	5.83	6.04	6.45
Quercetin	5.63	6.22	6.85	5.26

### 3.3 Sini San improves CUMS-induced depression-like behavior of rats

**Figure 3:**
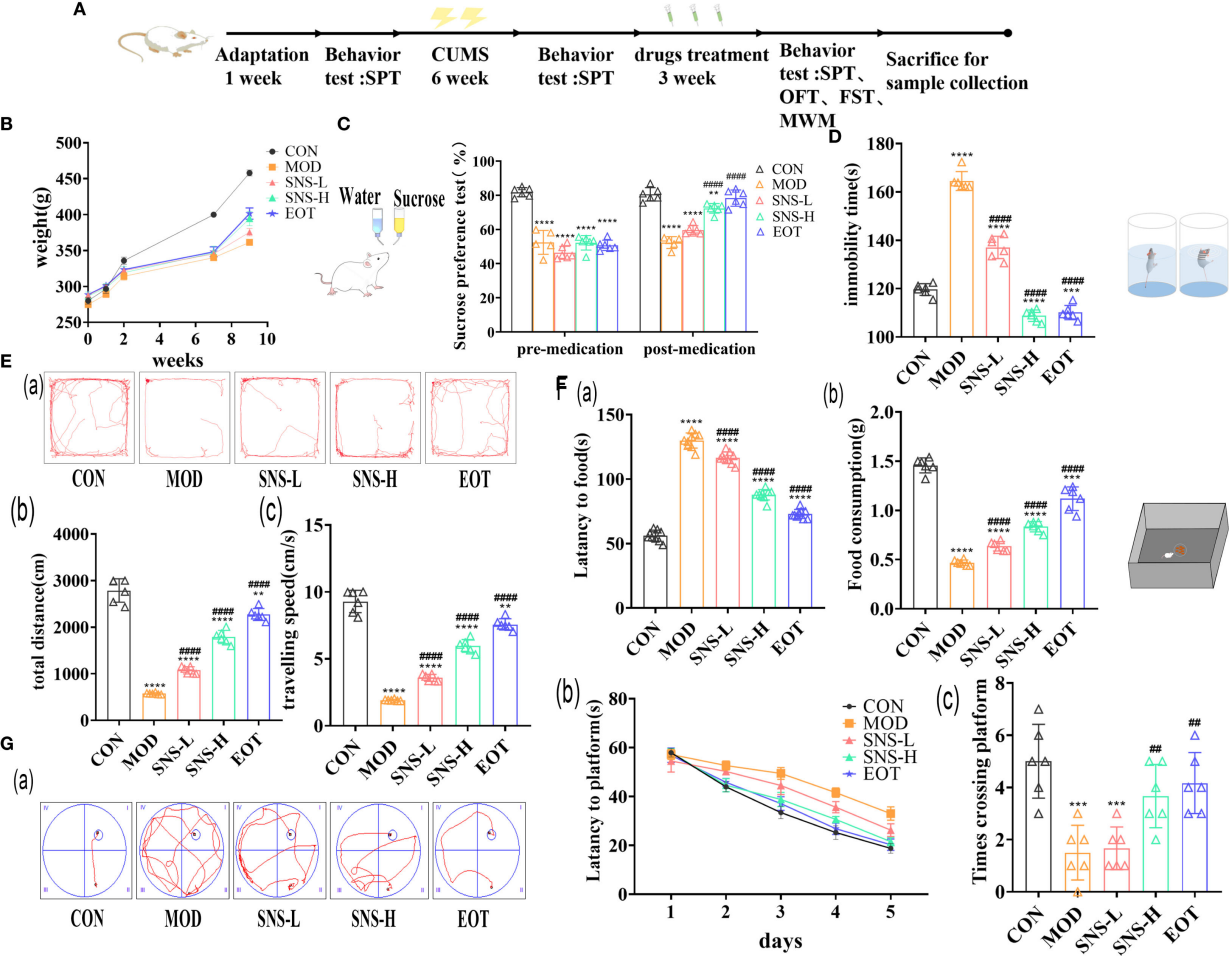
Evaluation of the efficacy of behavioral experiments on the treatment of depression by Sini San. **(A)** Experimental protocol. **(B)** Body weight change, determination of the body weight of rats after one week of adaptive feeding, one week of CUMS intervention, six weeks of CUMS intervention, and three weeks of pharmacological treatment. **(C)** Preference for Sucroce, determining the preference level of rats for sucrose solution after CUMS intervention and subsequent drug treatment. **(D)** Duration of immobility, measured the duration of immobility in each group of rats in water to assess their stress response after CUMS intervention and drug treatment. **(Ea–c)**. Trajectory, total distance travelled and speed of movement, statistics and analysis the distance **(Eb)** and speed **(Ec)** of rats in an open field to reflect their spatial exploration abilities, with trajectory **(Ea)** providing a more intuitive representation **(Fa, b)**. Latency, Food consumption, the curiosity of rats about novel food was reflected by measuring the time of first exposure to the novel food **(Fa)** and the consumption of food **(Fb)** ingested by rats. **(Ga–c)**. Trajectory of movement, Latency change, Number of crossings of the plateau, in the pre-training phase, the time for the rats to find the platform **(Gb)** was recorded, and in the final testing phase, the number of times the rats traversed the platform **(Gc)** was recorded, and the combination of the two was used to reflect the learning and memory ability of the rats, and the trajectory **(Ga)** can be visualized as follows (^##/^** P<0.01, *** P<0.001, ^####/^**** P<0.0001 vs. CON group; # is consistent with * vs. MOD group).

CUMS stimulation in rats significantly reduced appetite as well as sucrose preference. After treatment with Sini San, the rats’ appetite increased and their interest in sugar water improved; body weight also rose (**Figure 3B**), and sucrose preference in the SPT was elevated (**Figure 3C**). CUMS induced anxiety-like behaviors and impaired stress resilience in rats. With Sini San treatment, anxiety was reduced and stress capacity improved. In the FST, immobility time in the MOD group was significantly higher than in the CON group, while immobility times in the SNS-L, SNS-H, and EOT groups gradually decreased (**Figure 3D**). In the OFT and NSF, spatial exploration and novelty-seeking behaviors were reduced after CUMS but improved after drug treatment. Compared with the CON group, MOD rats showed reduced distance traveled and movement speed (**Figures 3Eb, c**), increased latency, and decreased food consumption in the NSF (**Figures 3Fa, b**). In contrast, SNS-L, SNS-H, and EOT groups showed increased distance and speed, reduced latency, and greater food consumption compared with MOD. In the MWM, MOD rats had significantly longer latency (**Figures 3Gb**) and fewer platform crossings (**Figures 3Gc**) than CON rats. Treatment with *Sini San* (SNS-L, SNS-H) and EOT significantly reduced latency and increased the number of platform crossings compared with MOD. These findings indicate that CUMS-induced learning and memory deficits in rats were ameliorated by Sini San treatment.

### 3.4 Sini San ameliorates CUMS-induced inflammatory response of rats

**Figure 4:**
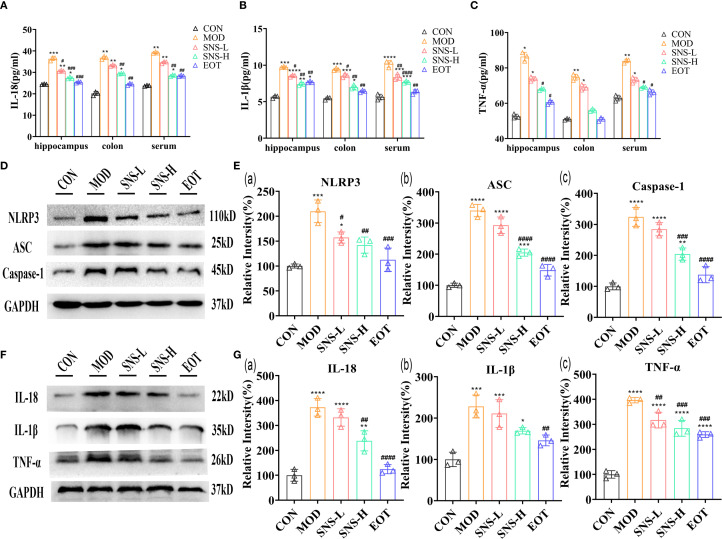
Improvement of inflammatory response in CUMS-induced rats by Sini San. **(A–C)**. The levels of inflammatory factor, IL-18 **(A)**, IL-1β **(B)** and TNF-α **(C)** in hippocampus, colon and serum were detected by ELISA, and CUMS induced an inflammatory response in the rat. **(D)** The level of NLRP3 signaling pathway protein in the hippocampus as a reflection of inflammation in rats by WB to reflect the treatment of CUMS-induced inflammation by Sini San. **(Ea–c)**. Densitometric quantification of NLRP3 versus GAPDH for experiments in **(D, Ea)**. Densitometric quantification of ACS versus GAPDH for experiments in **(D, Eb)**. Densitometric quantification of Caspase-1 versus GAPDH for experiments in **(D, Ec)**. **(F)** The level of NLRP3 signaling pathway downstream protein in the hippocampus as a reflection of inflammation in rats by WB to reflect the treatment of CUMS-induced inflammation by Sini San, also validate results **(A–C)**. **(Ga–c)**. Densitometric quantification of IL-18 versus GAPDH for experiments in **(F, Ga)**. Densitometric quantification of IL-1β versus GAPDH for experiments in **(F, Gb)**. Densitometric quantification of TNF-α versus GAPDH for experiments in **(F, Gc)**. (^#/^* P <0.05, ^##/^** P<0.01, ^###/^*** P<0.001, ^####/^**** P<0.0001 vs. CON group; # is consistent with * vs. MOD group).

As illustrated in the figure, CUMS induced inflammatory responses in both the central nervous system (CNS) and peripheral tissues of rats. Specifically, the concentrations of IL-18 (**Figure 4A**), IL-1β (**Figure 4B**), and TNF-α (**Figure 4C**) in the hippocampus, colon, and serum were significantly higher in the MOD group compared with the CON group. After treatment with Sini San, the levels of these cytokines were reduced, indicating that Sini San could mitigate inflammation in both peripheral and CNS regions. The NLRP3 inflammasome is known to be closely associated with inflammation. To determine whether the anti-inflammatory effect of Sini San involves NLRP3 inflammasome suppression, WB analysis was performed to detect NLRP3 and related proteins. Results showed that compared with the CON group, the levels of NLRP3, ASC, and caspase-1 increased in the MOD group (**Figures 4D, Ea–c**), along with downstream cytokines IL-18, IL-1β, and TNF-α (**Figures 4F, Ga–c**). In contrast, these protein levels decreased in the SNS-L, SNS-H, and EOT groups. These findings suggest that Sini San inhibits the NLRP3 inflammasome pathway, thereby ameliorating neuroinflammation.

### 3.5 Sini San improves CUMS-induced depression-like behavior of rats via BDNF/TrkB/PI3K/AKT pathway

**Figure 5:**
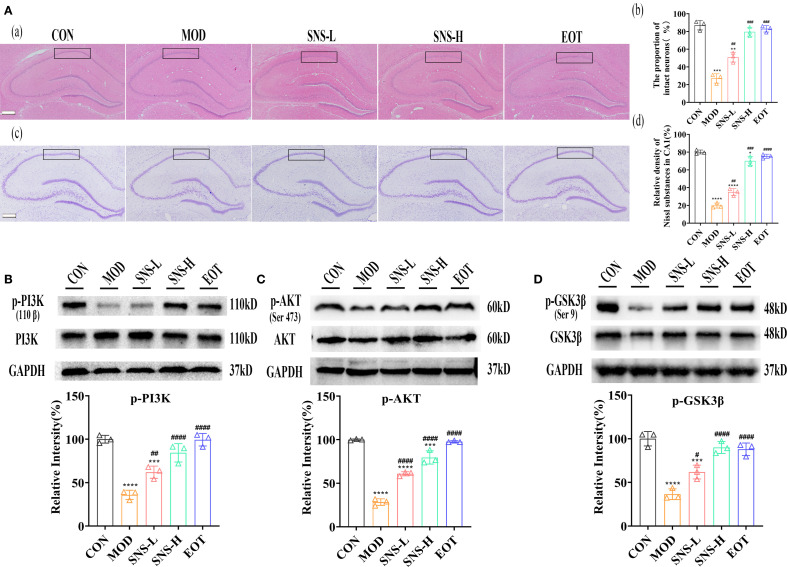
Sini San improves CUMS-induced depression in rats via PI3K/AKT pathway. **(Aa–d)**. HE staining of the hippocampus; The proportion of intact neurons in the CA1 region of the hippocampus; Nysted staining of the hippocampus; Relative density of Nissl substances in CA1. Stained the hippocampus by using HE **(Aa)** and Nysted staining **(Ab)**, and the improvement of the hippocampus of CUMS-intervened rats by Sini San was reflected by observing the number and morphology of hippocampal Nysted bodies. **(Ba, b)**. The level of protein of PI3K phosphorylation **(Ba)** in the hippocampus as a reflection of depressionin in rats by WB to reflect the treatment of CUMS-induced depression by Sini San. Densitometric quantification of p-PI3K versus total PI3K **(Bb)**. **(Ca, b)**. The level of protein of AKT phosphorylation **(Ca)** in the hippocampus as a reflection of depressionin in rats by WB to reflect the treatment of CUMS-induced depression by Sini San. Densitometric quantification of p-AKT versus total AKT **(Cb)**. **(Da, b)**. The level of protein of GSK3β phosphorylation **(Da)** in the hippocampus as a reflection of depressionin in rats by WB to reflect the treatment of CUMS-induced depression by Sini San. Densitometric quantification of p-GSK3β versus total GSK3β **(Db)**. (^#/^* P <0.05, ^##/^** P<0.01, ^###/^*** P<0.001, ^####/^**** P<0.0001 vs. CON group; # is consistent with * vs. MOD group).

**Figure 6:**
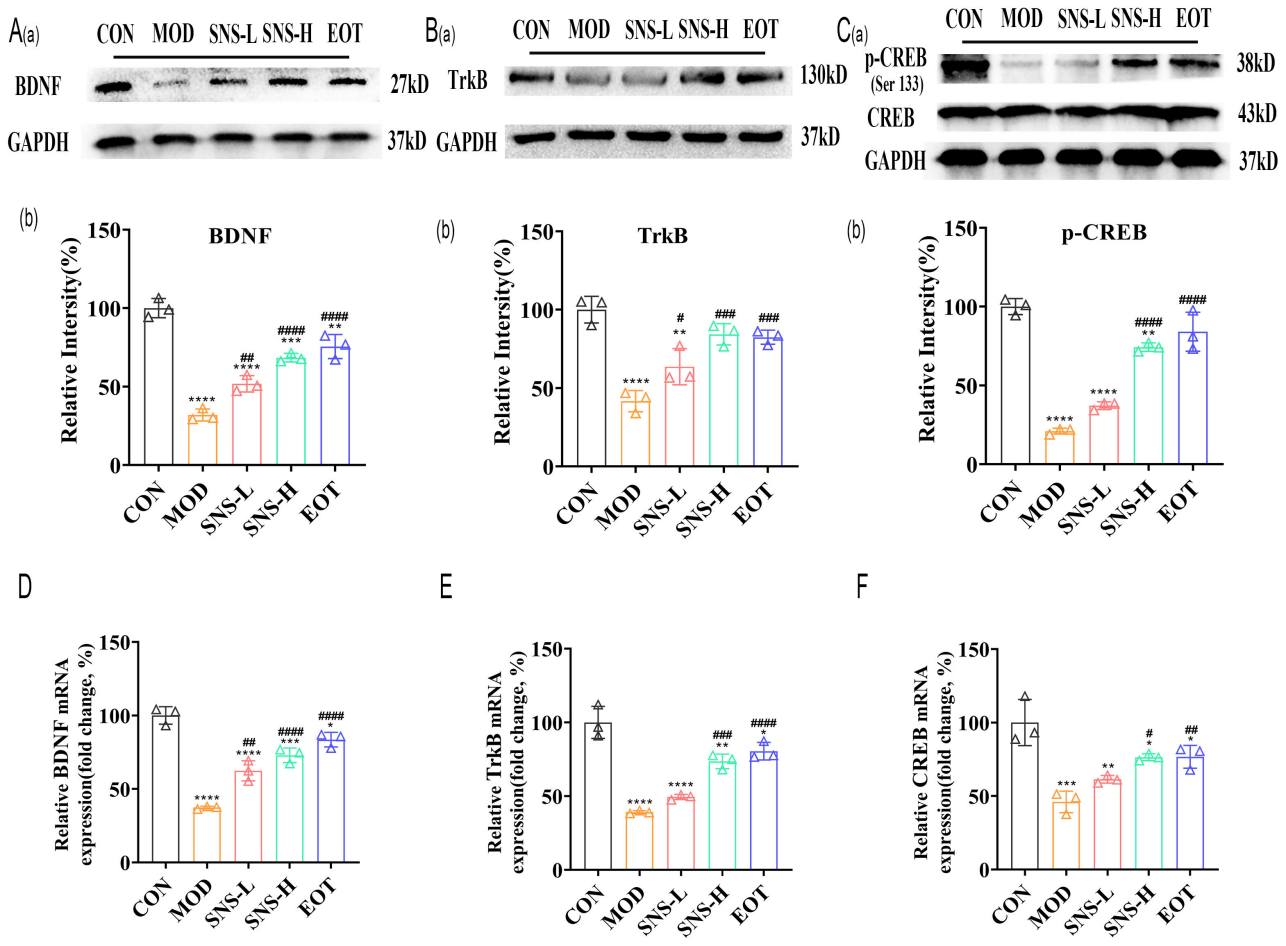
Sini San improves CUMS-induced depression in rats via BDNF/TrkB pathway. **(Aa, b)** The level of upstream protein of PI3K/AKT pathway as BDNF **(Aa)** in the hippocampus as a reflection of depressionin in rats by WB to reflect the treatment of CUMS-induced depression by Sini San. Densitometric quantification of BDNF versus GAPDH **(Ab)**. **(Ba, b)** The level of upstream protein of PI3K/AKT pathway as TrkB **(Ba)** in the hippocampus as a reflection of depressionin in rats by WB to reflect the treatment of CUMS-induced depression by Sini San. Densitometric quantification of TrkB versus GAPDH **(Bb)**. **(Ca, b)** The level of downstream protein phosphorylation of PI3K/AKT as CREB **(Ca)** in the hippocampus as a reflection of depressionin in rats by WB to reflect the treatment of CUMS-induced depression by Sini San. Densitometric quantification of p-CREB versus total CREB **(Cb)**. **(D–F)** The mRNA level of protein of BDNF/Trkb/CREB pathway **(Aa)** in the hippocampus as a reflection of depressionin in rats by RT-qPCR, densitometric quantification of BDNF **(D)**, TrkB **(E)**, CREB **(F)** versus GAPDH, consistent with the WB results. (^#/^* P <0.05, ^##/^** P<0.01, ^###/^*** P<0.001, ^####/^**** P<0.0001 vs. CON group; # is consistent with * vs. MOD group).

Was the amelioration of CUMS-induced depression-like behavior by Sini San related to neuronal loss? We subsequently examined the morphological changes of hippocampal neurons in rats using HE staining and Nissl staining to assess neuron number and Nissl body distribution. In the CON group, a large number of Nissl bodies with regular morphology were observed, whereas in the MOD group, many Nissl bodies were lost, severely disassembled, and irregular in morphology (**Figure 5A**). The number of Nissl bodies increased in the SNS-L, SNS-H, and EOT groups, with greater increases in the SNS-H and EOT groups; morphology in these groups was also more regular and complete compared with the MOD group. What are the underlying mechanisms of these phenomena? To explore the underlying mechanisms of these phenomena, network pharmacology analysis of core regulatory targets and their association with disease progression, together with semi-flexible molecular docking and compound affinity prediction, identified the PI3K/AKT pathway and related proteins as key targets. WB analysis showed that the levels of p-PI3K/PI3K, p-AKT/AKT, and p-GSK3β/GSK3β (**Figures 5B–D**) were significantly reduced in the MOD group compared with the CON group. These protein levels were elevated in the SNS-L, SNS-H, and EOT groups, with the highest increases in the SNS-H and EOT groups, suggesting that these changes could be reversed by the treatment. Further investigation of the PI3K/AKT pathway’s upstream (BDNF, TrkB) and downstream (CREB) depression-related proteins demonstrated that CUMS induction significantly reduced hippocampal levels of BDNF, TrkB, and p-CREB/CREB (**Figures 6A–C**), as detected by WB. These reductions were significantly reversed by Sini San treatment. Consistent results were observed at the mRNA level for BDNF, TrkB, and CREB through RT-qPCR analysis (**Figures 6D–F**), confirming the protein-level findings.

These data collectively indicate that Sini San ameliorates depression-like behaviors in CUMS-induced rats by activating the BDNF/TrkB/PI3K/AKT/CREB signaling axis.

### 3.6 Sini San balancing intestinal flora by repairing the integrity of the intestinal wall, reversing intestinal flora differences and improving intestinal function

**Figure 7:**
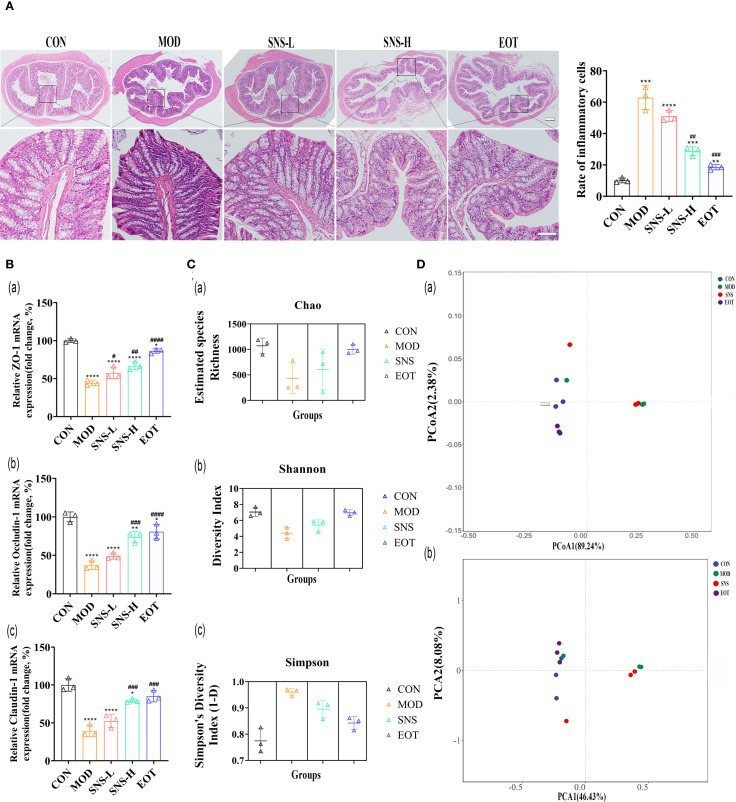
Balancing CUMS-induced intestinal flora in rats by Sini San. **(A)** HE staining of the Colonic, rate of inflammatory cell. Comparing the integrity of intestinal wall as well as the infiltration inflammatory cell in each group of rats to reflect the treatment of intestinal effects in CUMS-intervened rats by Sini San. **(Ba–c)**. The mRNA expression level of colonic junction protein was determined by RT-qPCR to reflect the disruption of the integrity of intestinal wall by CUMS and the repair of the intestinal wall by Sini San, which was incidentally validated in experiment **(A, Ca–c)**. Analysis of intestinal contents in terms of alpha diversity. Differences in intestinal flora among groups of rats using the Chao index **(Ca)**, Shannon index **(Cb)** and Simpson index **(Cc)**. **(Da, b)**. Analysis of intestinal contents in terms of beta diversity. Differences in intestinal flora of rats in each group by PcoA **(Da)** and PCA analysis **(Db)**. (^#/^* P <0.05, ^##/^** P<0.01, ^###/^*** P<0.001, ^####/^**** P<0.0001 vs. CON group; # is consistent with * vs. MOD group).

Histopathological examination of colon tissue showed that CUMS disrupted the arrangement of glandular cells and compromised the integrity of the intestinal mucosa, and Sini San treatment inhibited the histological damage in the colon (**Figure 7A**). There was a large number of inflammatory cell infiltration in the colonic tissues after the induction of CUMS, whereas Sini San treatment significantly reduced inflammatory infiltration of the colon, which was in line with the results of the previous ELISA (**Figures 4A–C**). The RT-qPCR results demonstrated that mRNA expression of colonic tight junction proteins (ZO-1, Occludin-1, Claudin-1) was significantly decreased in the MOD group compared to the CON group (**Figures 7Ba–c**). In contrast, a progressive increase was observed in the SNS-L, SNS-H and EOT groups, corroborating the pathological findings. The previous experiments indicated that high concentration of Sini San was effective in CUMS-induced inflammation and depression, so the intestinal contents of rats in CON, MOD, SNS-H and EOT groups were selected for bacterial flora analysis. The α-diversity of rat intestinal flora showed significant differences between the MOD and CON groups: the Chao index (**Figures 7Ca**) and Shannon index (**Figures 7Cb**) were significantly decreased, while the Simpson index (**Figures 7Cc**) was increased in the MOD group. Compared with the MOD, the Chao index and Shannon index were significantly increased and the Simpson index was markedly decreased in the SNS-H and EOT groups. The most pronounced changes were observed in the EOT group. For the β-diversity of rat intestinal flora, Principal Coordinate Analysis (PCoA) (**Figures 7Da**) revealed no significant overlap between the CON, EOT and MOD groups, indicating distinct microbial community structures. In contrast, partial overlap was observed between the SNS-H and MOD groups. Principal Component Analysis (PCA) results (**Figures 7Db**) were consistent with those of PCoA (**Figures 7Da**), showing similar clustering patterns among groups.

**Figure 8:**
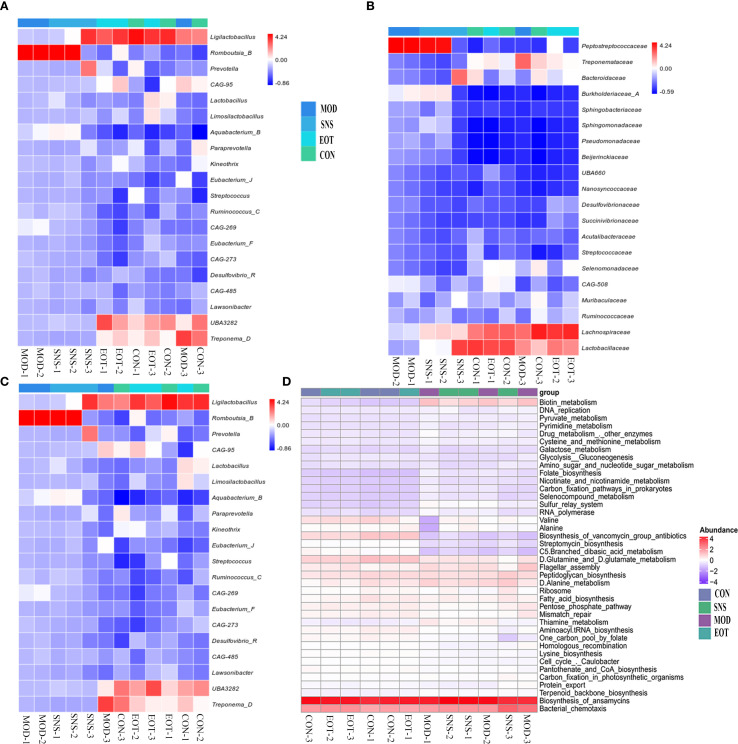
Reversal of intestinal flora differences and improvement of intestinal function by Sini San. **(A)** Analyzing the differences in intestinal flora in rats at the phylum to reflect the balance of intestinal flora in CUMS-intervened rats by Sini San. **(B)** Analyzing the differences in intestinal flora in rats at the family to reflect the balance of intestinal flora in CUMS-intervened rats by Sini San. **(C)** Analyzing the differences in intestinal flora in rats at the genus in oredr to reflect the balance of intestinal flora in CUMS-intervened rats by Sini San. **(D)** Results of the analysis of Rat Enterobacteriaceae PICRUSt2, predicting differences in function based on differences in intestinal flora among groups of rats.

From the quantitative results, at the level of the phylum, Ligilactobacillus, UBA3238, Treponema-D and Romboutsia-B differed significantly in the MOD and CON groups, and the proportions of Ligilactobacillus, UBA3238, Treponema-D in the intestinal tract of CUMS-induced rats were relatively low, while the proportion of Romboutsia*-*B was relatively high, which was altered by Sini San treatment (**Figure 8A**). At the family level, the proportion of Peptostreptococcaceae increased in the MOD group compared with the CON group, and the proportion of Treponemataceae, Bacteroidaceae, Lachnospiraceae and Lactobacillaceae decreased, and the difference between the MOD group and the CON group was obvious in the above-mentioned items, and the Sini San treatment reversed the above changes (**Figure 8B**). At the genus level, the proportion of Ligilactobacillus, CAG-95, Aquabacterium*-*B, UBA3282, and Treponema*-*D was reduced in the MOD group compared to the CON group, and increased in the proportion of Romboutsia-B, and the difference was significant, and the above genera of intestinal bacteria were improved after the treatment of Sini San (**Figure 8C**). In PICRUSt2 metabolic function prediction, the difference between the MOD group and SNS group was not significant, nor were those between the CON group and EOT group. However, a significant difference was observed between the CON group and MOD group. Compared to the CON group, the MOD group showed significant alterations in biotin metabolism, biosynthesis of vancomycin group antibiotics, streptomycin biosynthesis, branched-chain dibasic acid metabolism, thiamine metabolism and the one-carbon pool by folate pathway (**Figure 8D**).

## 4 Discussion

For the construction of depression models, it is recognized that CUMS, olfactory bulb destruction, etc., CUMS is the most widely used (29). In this study, CUMS was employed to establish depression model rats, with successful modeling confirmed through behavioral assessments including the SPT, FST, OFT and so on (**Figure 3**). The treatment of depression in Chinese medicine is based on the principle of relieving the liver and resolving depression, and the therapeutic formulas include Jieyu Anshen Granules, Sini San, Chaihu Shugan San, Xiaoyao San, etc. Through intervention with the classical TCM formula Sini San, this study demonstrated its antidepressant effects in depressed rats by ameliorating inflammatory responses and restoring gut microbiota balance. Network pharmacology analysis further identified the PI3K/Akt signaling pathway as a key regulatory target (**Figure 2C**). In the inflammatory response accompanying the onset of depression, TLR4, indoleamine 2,3-dioxygenase (IDO) and NLRP3 play important roles, with activation of NLRP3 inflammasome being more critical. In addition, it has been found that psychological stress activates the NLRP3 inflammasome pathway, leading to the assembly of NLRP3 with the adaptor protein ASC and procaspase-1. This complex triggers the autocleavage of caspase-1, which subsequently cleaves and activates the pro-inflammatory cytokines IL-1β and IL-18 (30). Subsequently, the downstream signal transduction pathways were activated and large amounts of inflammatory mediators were released, such as TNF-α (**Figures 4B–D**), which cross the blood-brain barrier (BBB) to induce dysregulation of neurotransmitters and promote depression-like behaviors (47–49). Activation of the NLRP3 inflammasome induces metabolite dysregulation and functional changes in intestinal flora, which exacerbate neuroinflammation and depression-like behaviors through the “gut-brain axis” (**Figure 4A**) (48, 50).

Phosphatidyl-inositol 3-kinase (PI3K) is associated with changes in synaptic plasticity, learning and memory (29) as well as depression-related alterations (51). Then, AKT (also called protein kinase B, PKB), as a downstream effector of PI3K, serves as a critical regulator of neuronal survival, growth, and synaptic plasticity. In the same way, glycogen synthase kinase 3 beta (GSK-3β), as a downstream effector of PI3K/AKT signaling, has emerged as a promising therapeutic target for affective disorders through its regulation of neuroplasticity and inflammatory pathways. PI3K activation mediates AKT phosphorylation, which subsequently regulates GSK-3β activity through Ser9 phosphorylation, thereby modulating depressive-like behaviors via regulation of synaptic plasticity and neuroinflammation (52–54). PI3K/AKT/GSK-3β plays a key role in neuronal development and synaptic plasticity, and is a critical therapeutic target pathway for many antidepressants (55–57). Forming a closed loop with upstream BDNF/TrκB as well as downstream cAMP-responsive element-binding protein (CREB), the PI3K/AKT pathway can lead to the phosphorylation of CREB. Next, p-CREB can mediate BDNF expression, which in turn activates TrκB, and activated TrκB in turn activates the PI3K/AKT pathway (58, 59). In the present study, CUMS-induced rats treated with Sini San had increased levels of phosphorylation of PI3K and AKT, subsequently activated GSK-3β (**Figures 5B–D**), upregulated the upstream protein BDNF and TrκB expression as well as increased the phosphorylation level of downstream CREB (**Figure 6**). Moreover, it has been found that the PI3K/AKT signaling pathway and NLRP3 inflammasome are linked in the pathogenesis of depression, and the activation of the former stimulates the expression of downstream nuclear factor-κB (NF-κB). At the same time, activated NF-κB stimulates the activation of the NLRP3 inflammasome and the production of inflammatory factors, such as IL-18, which in turn induced PI3K/AKT signaling pathway after IL-18 binding to its corresponding receptor (55). Research suggests IL-1β and IL-18 released after NLRP3 inflammasome activation can downregulate the PI3K/AKT pathway by inhibiting TrkB receptor expression or blocking its phosphorylation (60). Conversely, studies suggest that PI3K/AKT pathway activation can inhibit NLRP3 inflammasome assembly through phosphorylation or promote its ubiquitination and degradation, thereby reducing inflammatory factor release (61). Furthermore, BDNF-mediated TrkB receptor activation of the PI3K/AKT pathway upregulates SIRT6 and other deacetylases, indirectly suppressing NLRP3 transcription (62, 63). Thus, the regulatory relationship between NLRP3 inflammasome activation and BDNF/TrkB/PI3K/AKT is bidirectional, highlighting the complexity of disease pathogenesis and therapeutic challenges. Finding the balance between these pathways may offer new directions for depression treatment. In the present study, BDNF/TrkB/PI3K/AKT was used as the core pathway, combining its upstream and downstream substrates and NLRP3 inflammasome pathway together to explore the therapeutic mechanism of Sini San in depression. This approach initially validated our experimental hypothesis.

In an analysis of the rat intestine, it was observed that CUMS disrupted intestinal integrity, which could be repaired by Sini San (**Figures 7A, B**). Using 16S DNA sequencing technology to analyze the intestinal flora of CUMS-induced rats, Sini San significantly increased the Chao and Shannon indices and decreased the Simpson index (**Figure 7C**). Moreover, in terms of β-diversity, the intestinal flora difference between CUMS-induced and normal rats was evident (**Figure 7B**), indicating that Sini San could restore intestinal flora diversity. Further analysis of intestinal flora composition showed that after Sini San treatment, the expression of Ligilactobacillus, UBA3238, and Treponema-D was upregulated at the phylum level (**Figure 8A**), while Romboutsia-B was downregulated. At the family level (**Figure 8B**), Treponemataceae, Bacteroidaceae, Lachnospiraceae, and Lactobacillaceae were enriched, whereas Peptostreptococcaceae was depleted. At the genus level (**Figure 8C**), there was a significant increase in the relative abundance of Ligilactobacillus, CAG-95, Aquabacterium-B, UBA3282, and Treponema-D, while Romboutsia-B showed marked depletion. Studies have shown that Ligilactobacillus and Treponema are potential anti-inflammatory bacteria with protective effects (64). Bacteroidaceae ferment carbohydrates into short-chain fatty acids (SCFAs), and SCFA deficiency leads to reduced intestinal barrier function. The gut wall disruption caused by CUMS may be related to this bacterium (65). Lachnospiraceae and Lactobacillaceae not only have anti-inflammatory effects but also play crucial roles in maintaining intestinal flora balance by inhibiting the proliferation of pathogenic bacteria and improving gut microbiota (64, 66, 67). Romboutsia is associated with anxiety (68), consistent with CUMS-induced anxiety-like behavior in rats. Other studies have shown that Ligilactobacillus can indirectly promote BDNF expression and enhance synaptic plasticity by increasing SCFA production (69). Its prebiotic effect may reduce neuroinflammation through competitive inhibition of potential pathogenic bacteria (e.g., Romboutsia), thereby maintaining intestinal microecological balance (70, 71). The mechanism includes reducing endotoxin (e.g., LPS) translocation into the bloodstream, consequently suppressing microglial activation and pro-inflammatory cytokine release (IL-1β, TNF-α), ultimately alleviating neuroinflammation. Therefore, these bacterial genera serve as important indicators of “gut–brain axis” dysfunction. Our results demonstrate that Sini San modulates the gut–brain axis, as evidenced by its regulatory effects on Lactobacillaceae and Romboutsia. Exploring dynamic changes in these genera in depression could reveal the clinical potential of microbiota transplantation or metabolite-targeted therapy, offering new breakthroughs for depression treatment. In brief, Sini San may exert anti-inflammatory and intestinal barrier–repairing effects by regulating the intestinal flora, which contributes to its antidepressant effects in CUMS-induced rats.

However, this experiment did not specifically target pathway proteins and lacked *in vitro* cellular validation. Furthermore, the study cannot fully verify the gut–brain axis because of the absence of reverse validation. In future experiments, we plan to use gene knockout and gene overexpression techniques in combination with inhibitor drugs to target specific proteins in specific pathways, further validating the accuracy of the mechanism. Validation will be conducted through both *in vitro* and *in vivo* experiments using LPS-induced HT22 or BV-2 cells. In addition, intestinal flora from depressed model rats will be transplanted into normal rats to perform reverse verification of the gut–brain axis, thereby further confirming the role of Sini San in treating depression through modulation of the gut–brain axis.

## 5 Conclusion

On the one hand, CUMS stimulation in rats can cause intestinal flora dysbiosis, further activating the NLRP3 inflammasome pathway, which releases inflammatory factors such as IL-18, IL-1β, and TNF-α. These inflammatory factors enter the bloodstream and then cross the BBB to induce neuroinflammation, leading to synaptic damage. On the other hand, CUMS induction also affects BDNF/TrkB and its downstream PI3K/AKT signaling pathway, resulting in synaptic damage, impaired synaptic plasticity, and ultimately depression. After treatment with Sini San, these changes were reversed, i.e., intestinal flora was balanced, inflammatory responses were alleviated, and the BDNF/TrkB and PI3K/AKT signaling pathways were activated, prompting phosphorylation of their pathway proteins to exert antidepressant effects (see Graphical Abstract).

In conclusion, we identified the main active compounds kaempferol, naringenin, and quercetin, and the PI3K/AKT signaling pathway associated with the treatment of depression by Sini San using network pharmacology and molecular docking methods. Further experiments demonstrated that Sini San exerts antidepressant effects by improving inflammatory responses and balancing intestinal flora through the PI3K/AKT and NLRP3 signaling pathways (Graphical Abstract).

## Data Availability

The original contributions presented in the study are included in the article/**Supplementary Material**, further inquiries can be directed to the corresponding author/s.
