# Supplementary figures and images for: Investigating the mechanisms of Sini San in alleviating inflammatory responses via multi-omics and the BDNF/TrkB/PI3K/AKT signaling pathway in depressive model rats
Source: Front Psychiatry. 2025 Oct 17;16:1628634. doi: 10.3389/fpsyt.2025.1628634 (PMC12575371; doi:10.3389/fpsyt.2025.1628634)

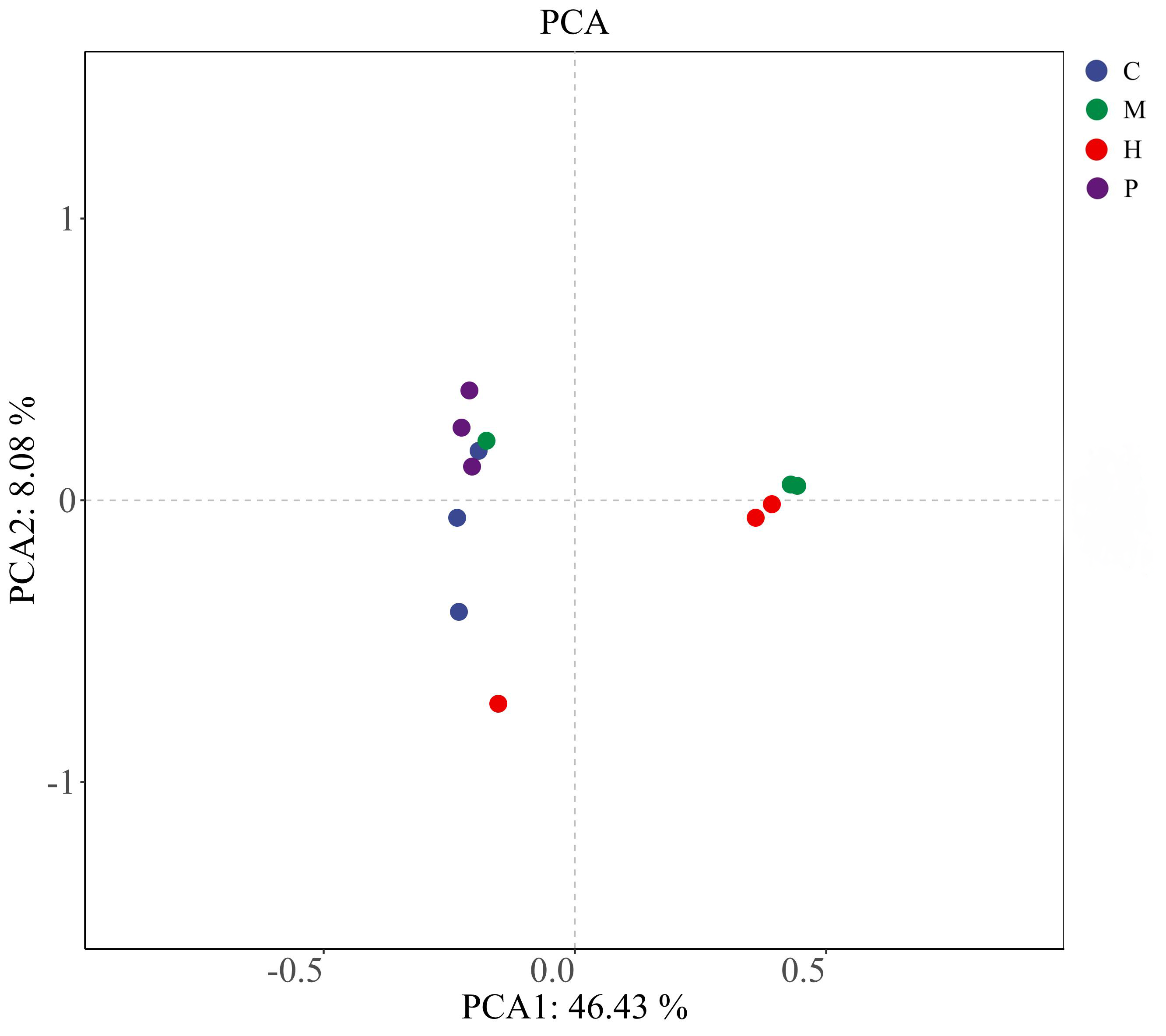

Supplement: Supplementary file 1 [file Presentation1.zip › Analysis of intestinal contents in terms of beta diversity/PCA.png]

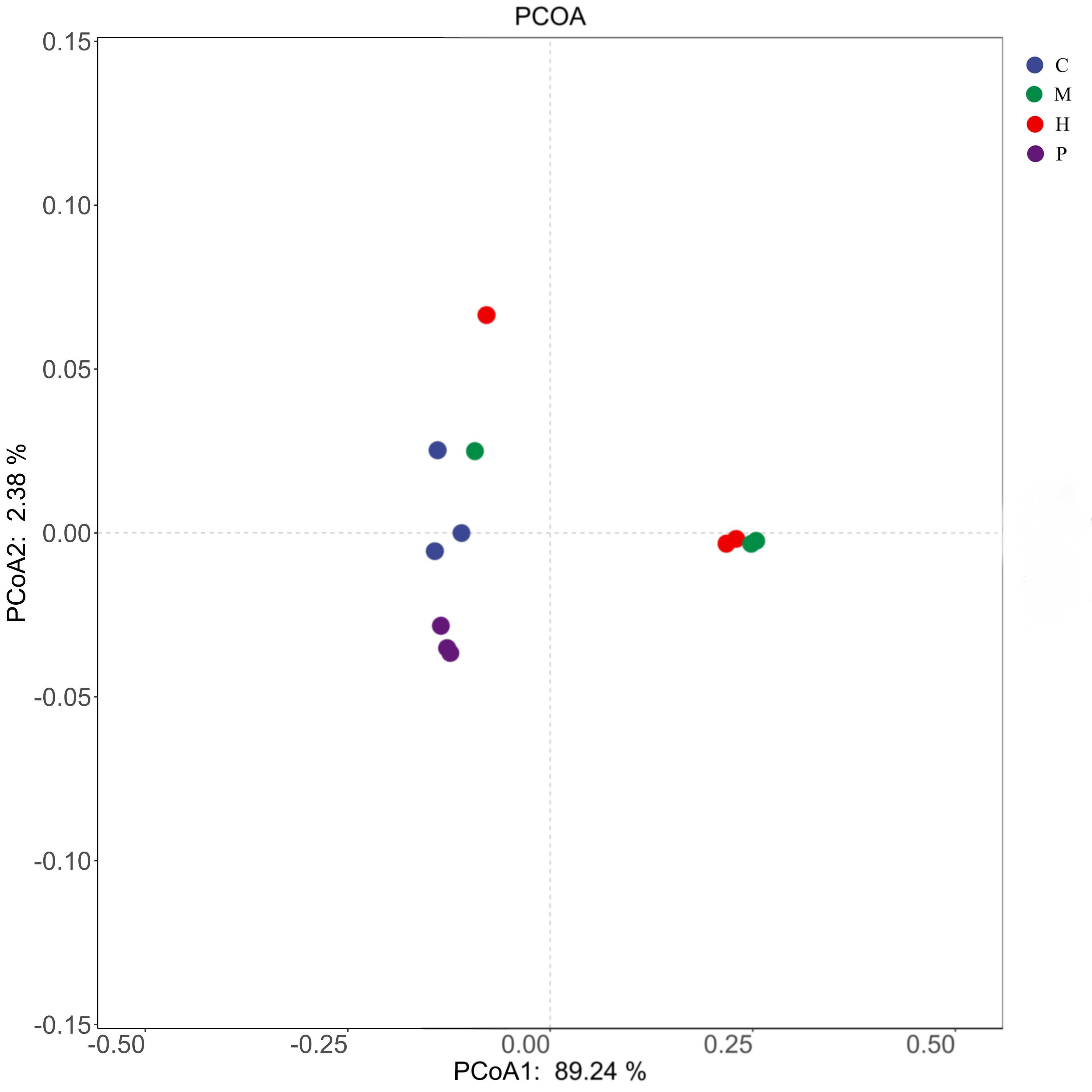

Supplement: Supplementary file 1 [file Presentation1.zip › Analysis of intestinal contents in terms of beta diversity/PCOA.png]

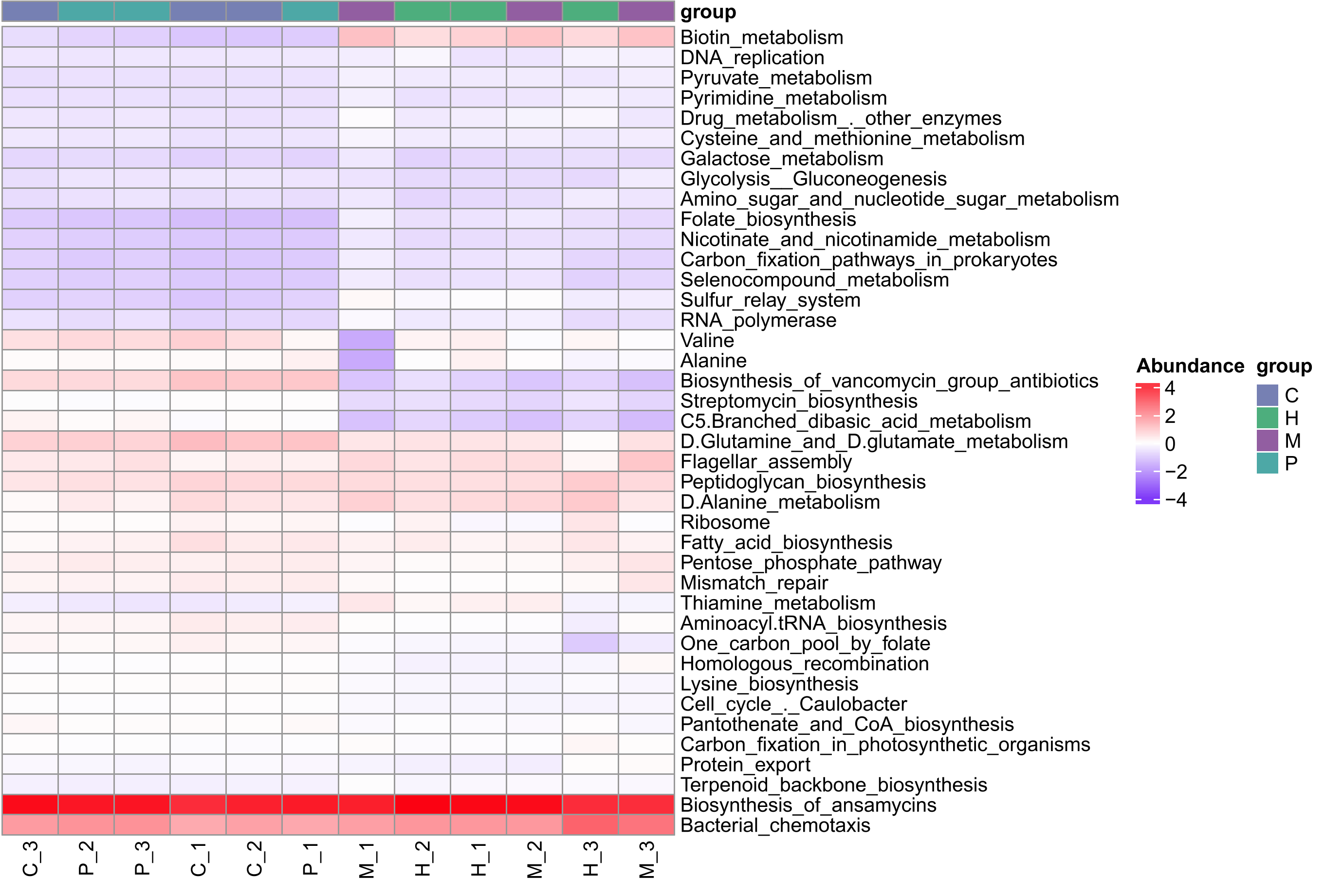

Supplement: Supplementary file 1 [file Presentation1.zip › Analysis of Rat Enterobacteriaceae PICRUSt2/PICRUSt2.PNG]

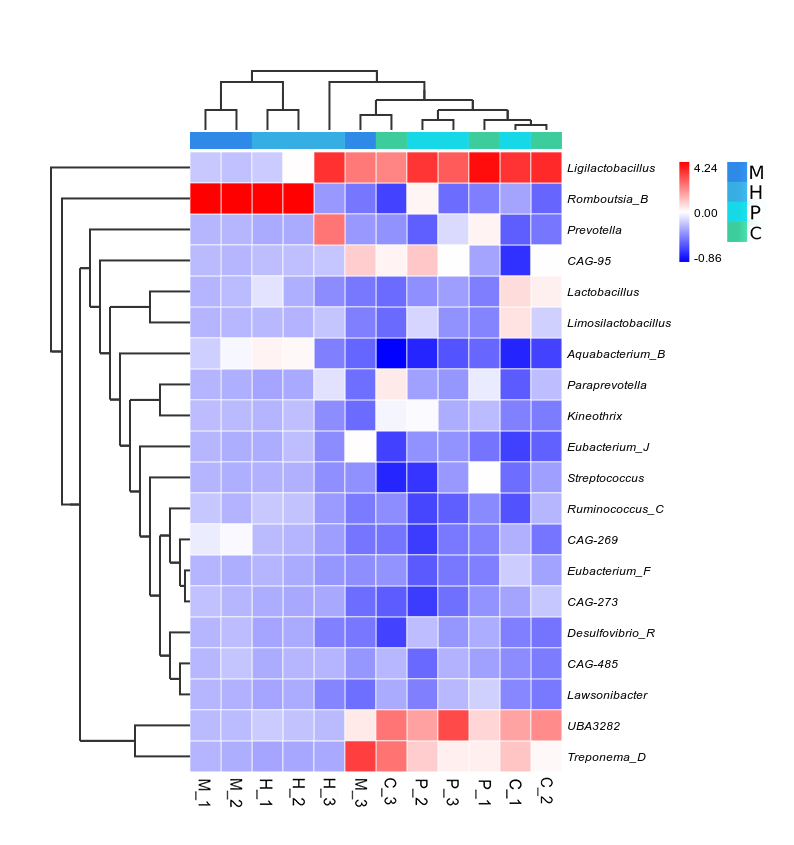

Supplement: Supplementary file 1 [file Presentation1.zip › Category/Family.png]

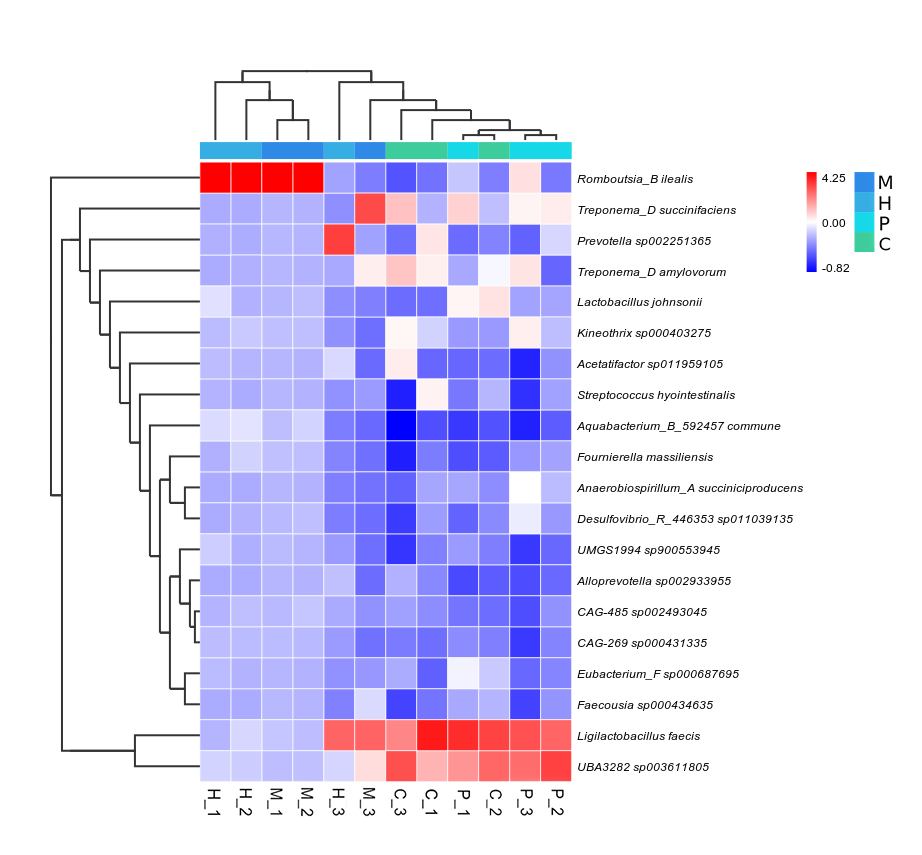

Supplement: Supplementary file 1 [file Presentation1.zip › Category/Genus.png]

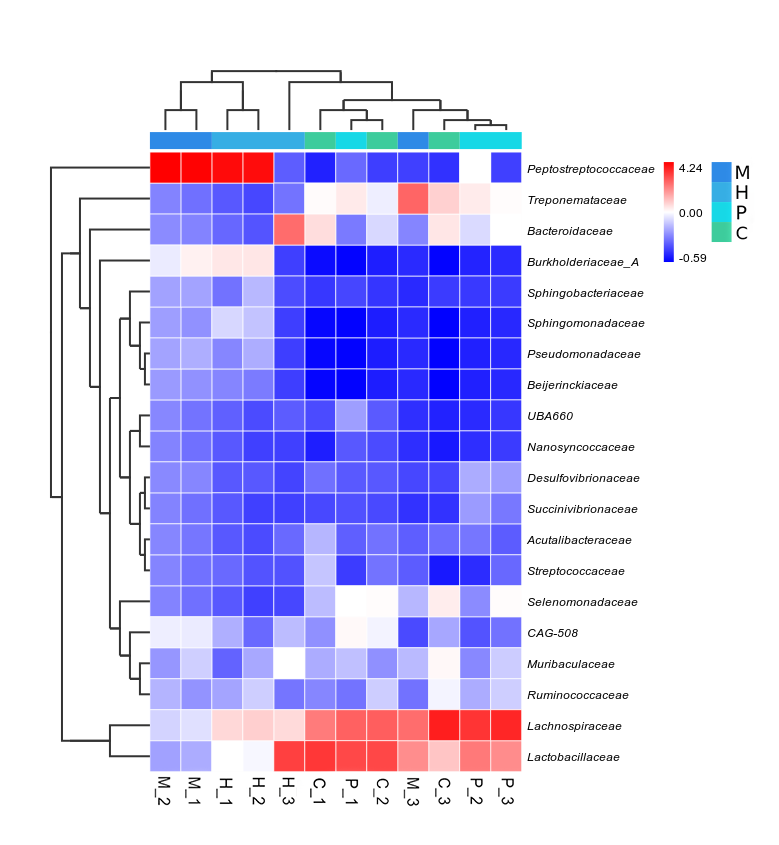

Supplement: Supplementary file 1 [file Presentation1.zip › Category/Phylum.png]

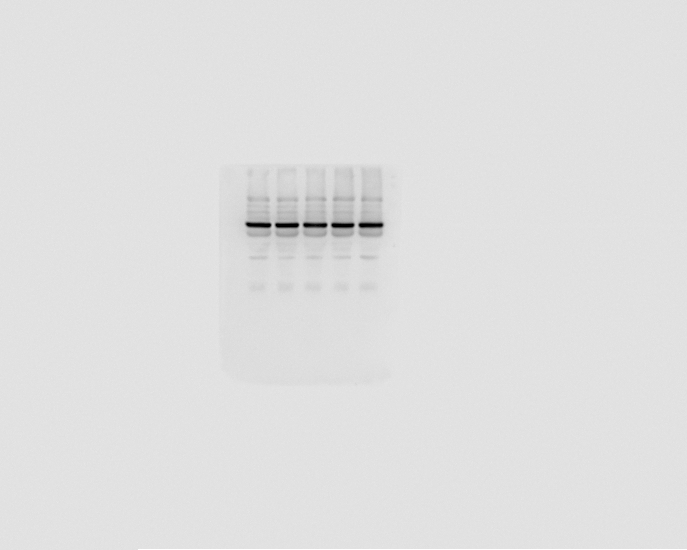

Supplement: Supplementary file 1 [file Presentation1.zip › Western Blot/AKT-2.tif]

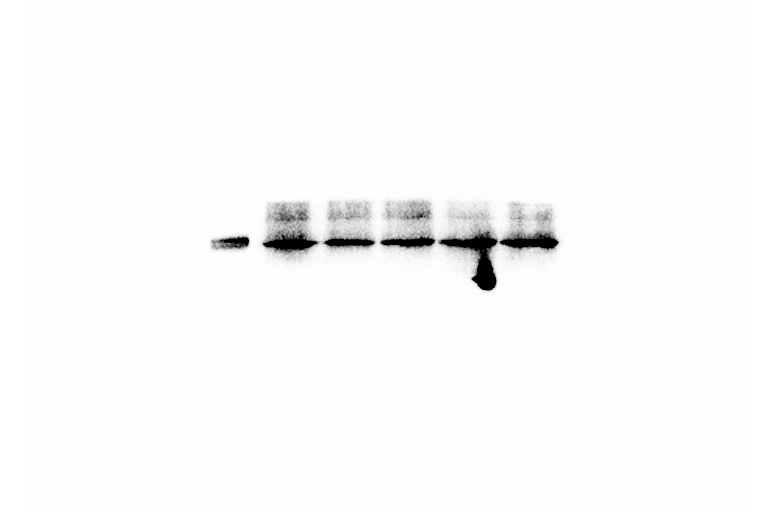

Supplement: Supplementary file 1 [file Presentation1.zip › Western Blot/AKT-3.tif]

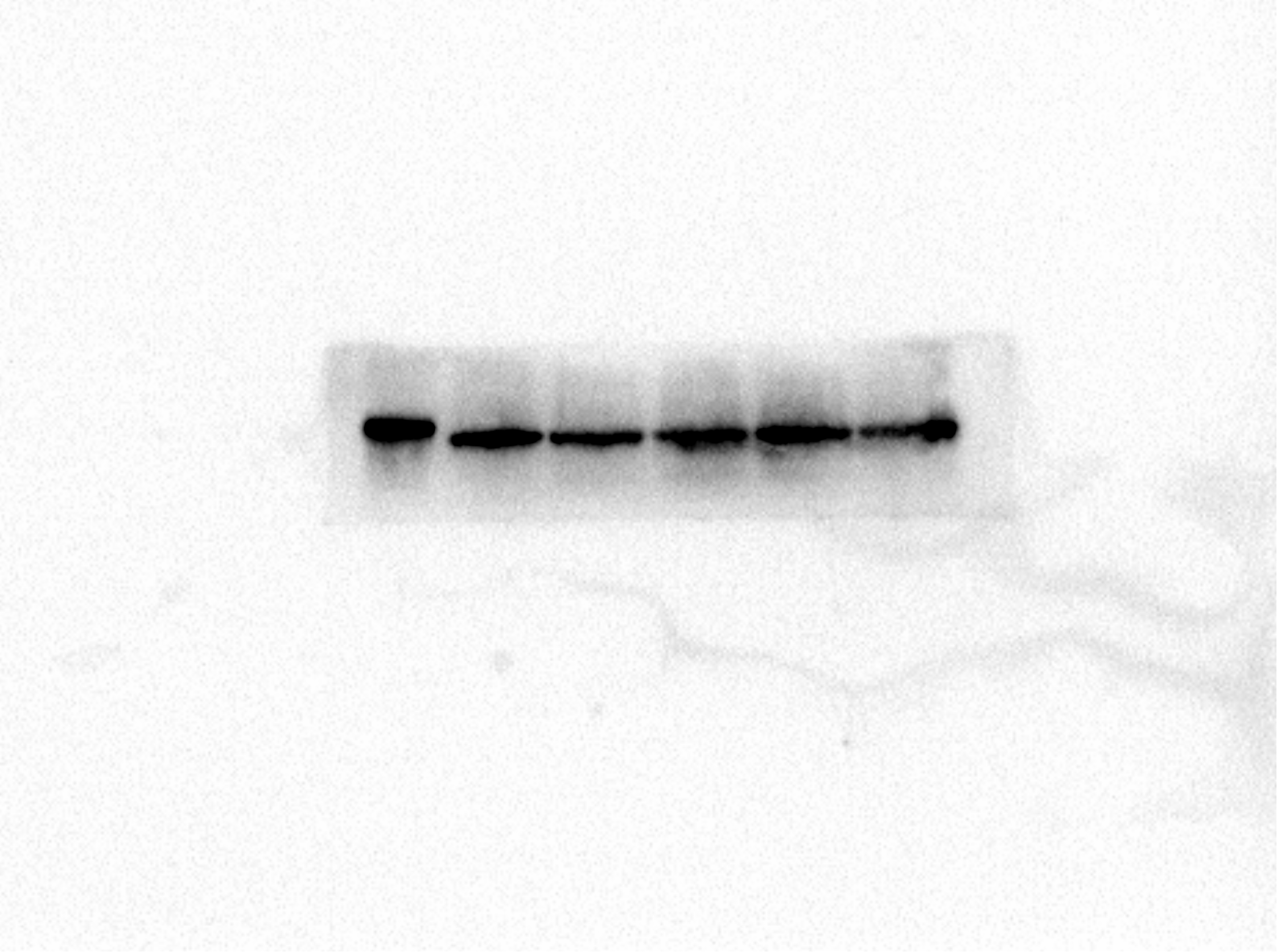

Supplement: Supplementary file 1 [file Presentation1.zip › Western Blot/AKT.tif]

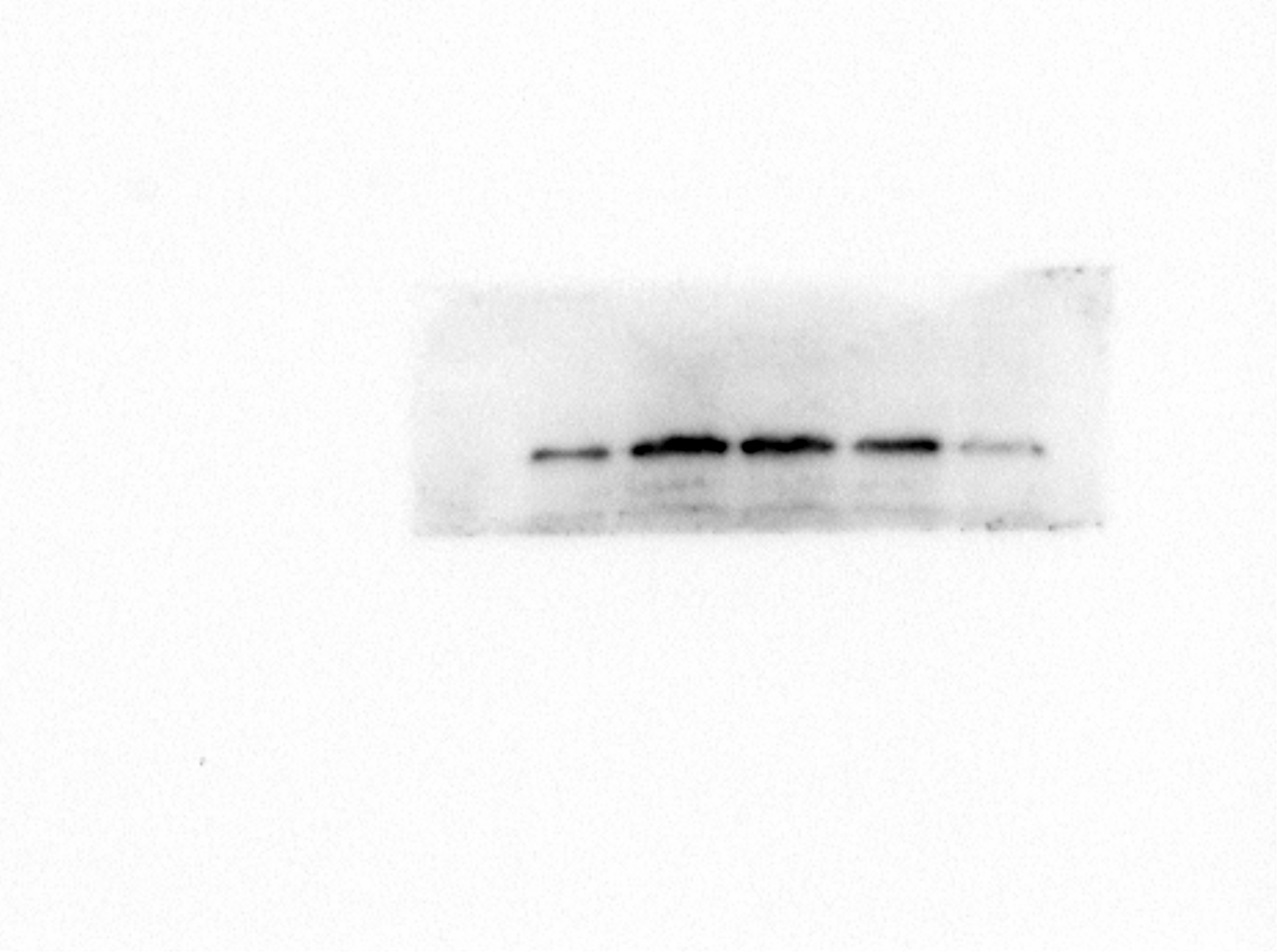

Supplement: Supplementary file 1 [file Presentation1.zip › Western Blot/ASC-2.tif]

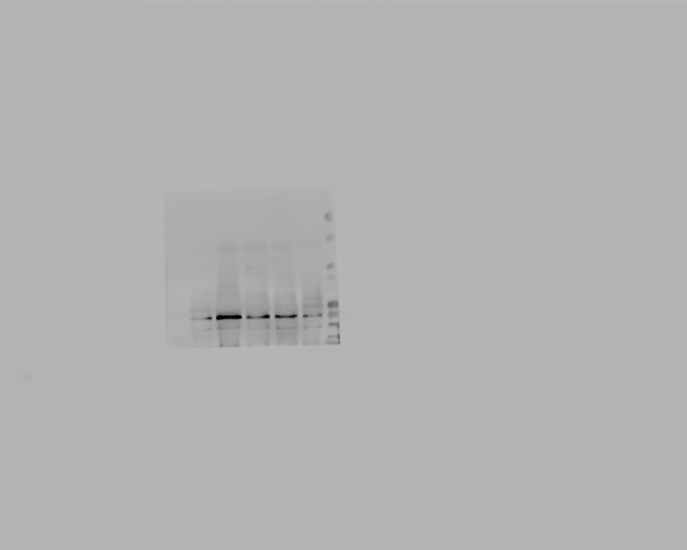

Supplement: Supplementary file 1 [file Presentation1.zip › Western Blot/ASC-3.tif]

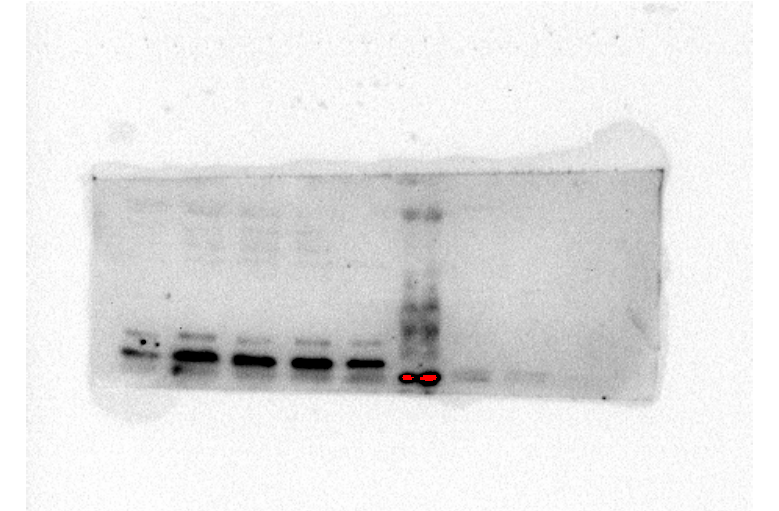

Supplement: Supplementary file 1 [file Presentation1.zip › Western Blot/ASC.tif]

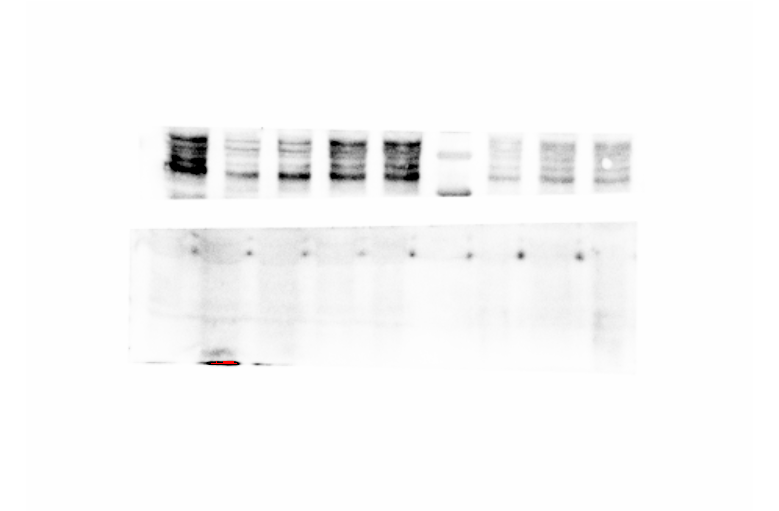

Supplement: Supplementary file 1 [file Presentation1.zip › Western Blot/BDNF-2.tif]

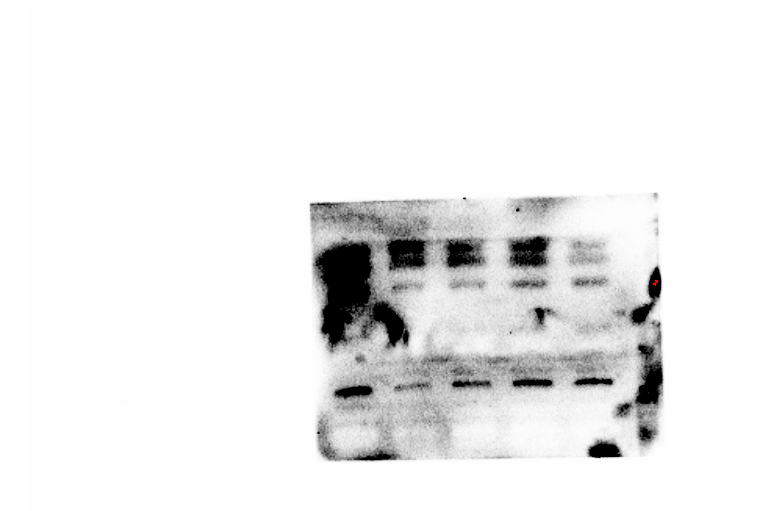

Supplement: Supplementary file 1 [file Presentation1.zip › Western Blot/BDNF-3.tif]

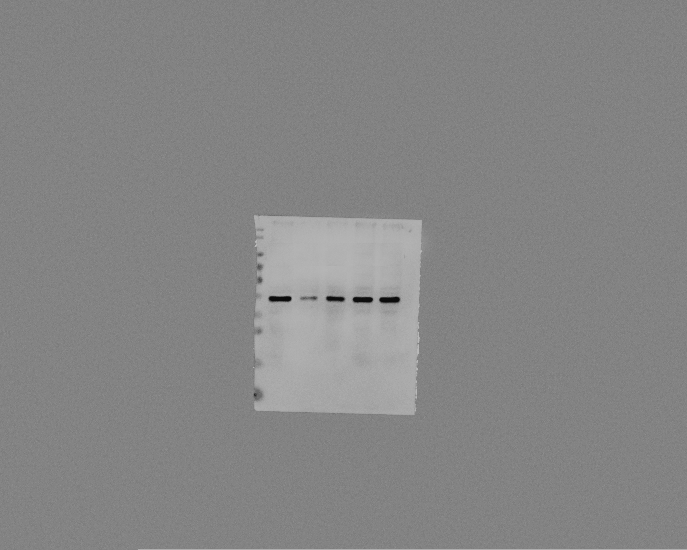

Supplement: Supplementary file 1 [file Presentation1.zip › Western Blot/BDNF.tif]

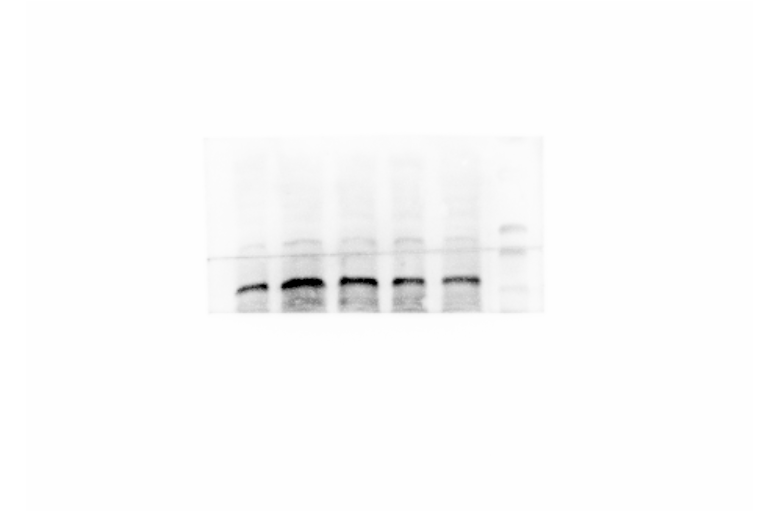

Supplement: Supplementary file 1 [file Presentation1.zip › Western Blot/Caspase-1-2.tif]

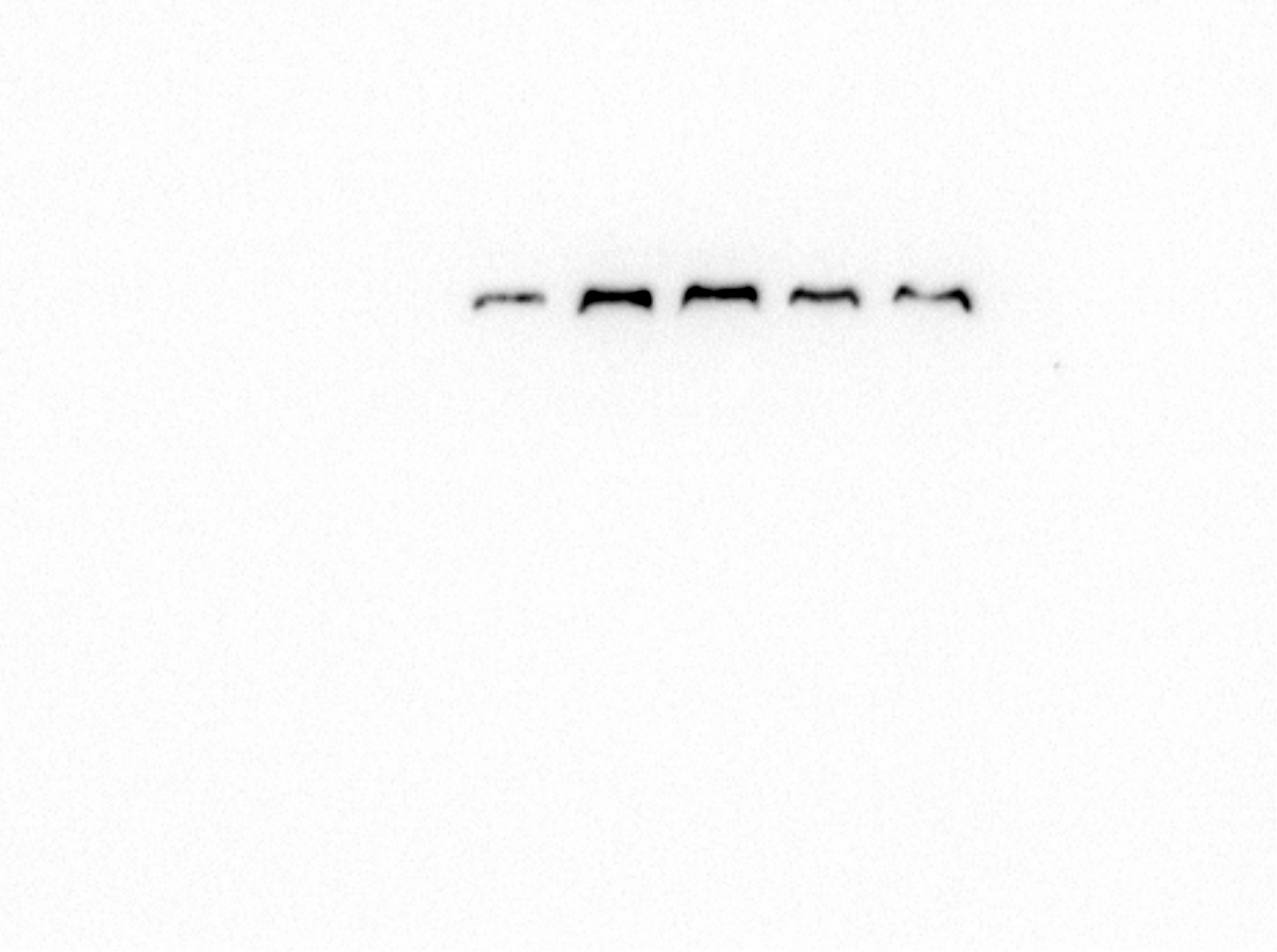

Supplement: Supplementary file 1 [file Presentation1.zip › Western Blot/Caspase-1-3.tif]

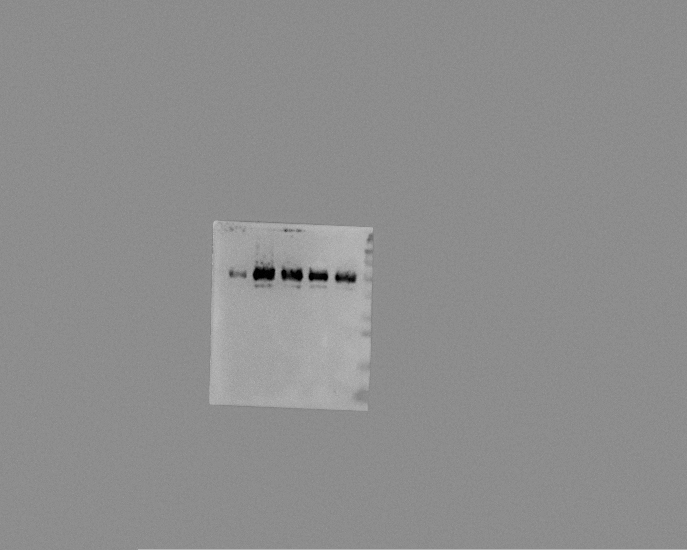

Supplement: Supplementary file 1 [file Presentation1.zip › Western Blot/Caspase-1.tif]

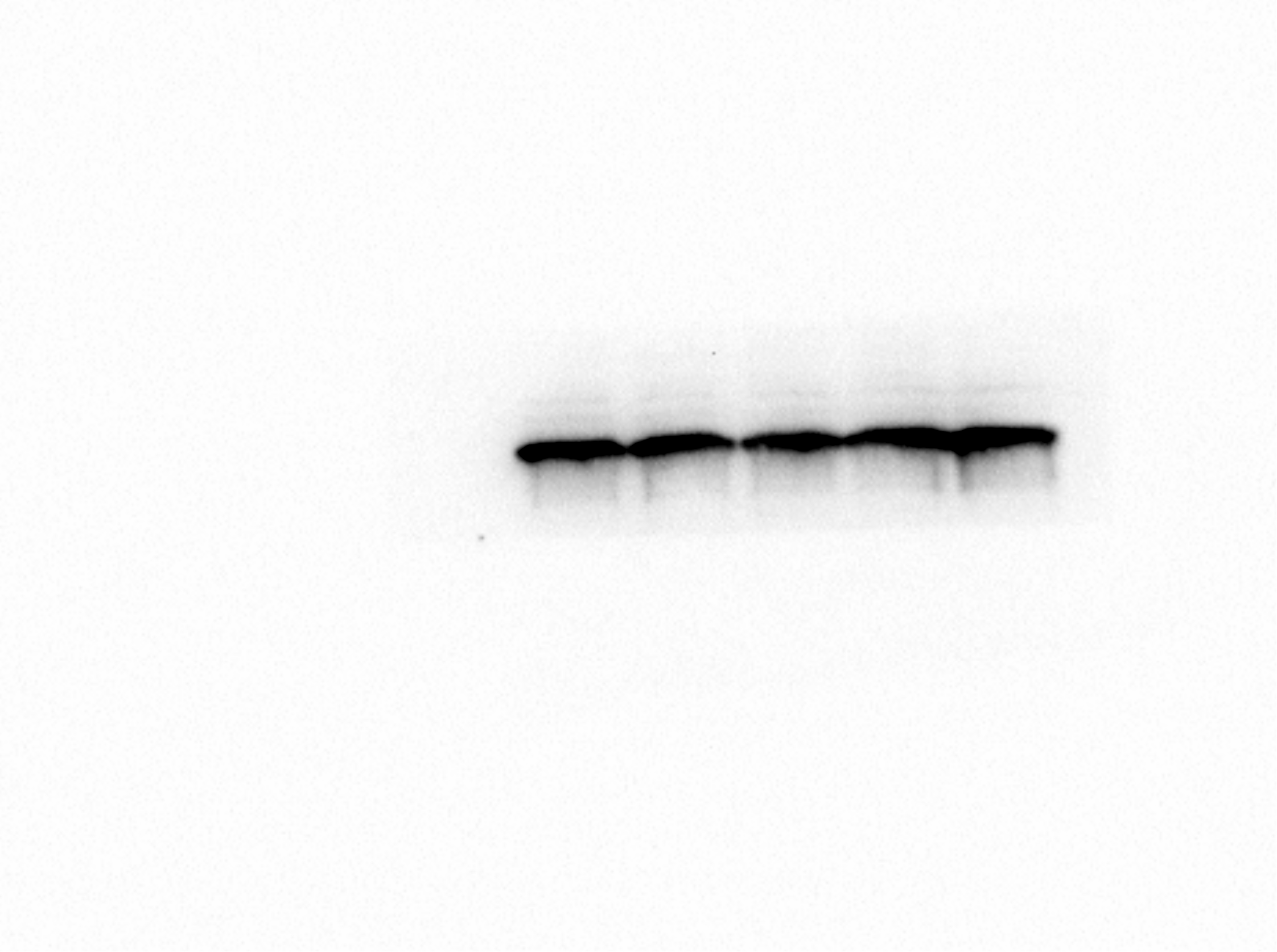

Supplement: Supplementary file 1 [file Presentation1.zip › Western Blot/CREB-2.tif]

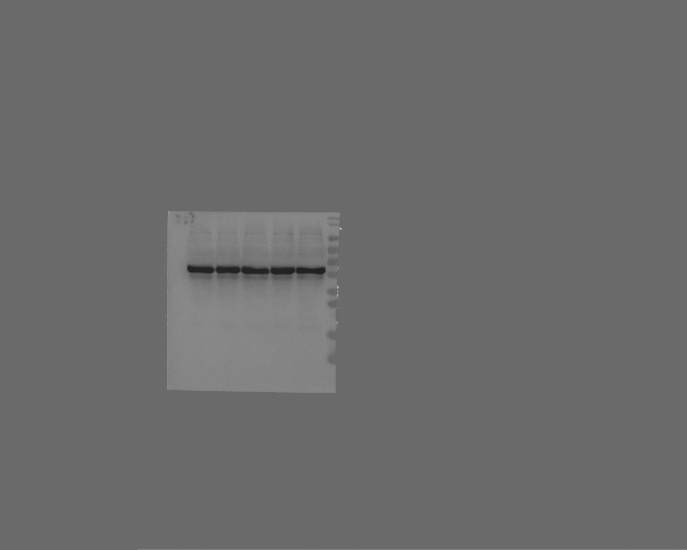

Supplement: Supplementary file 1 [file Presentation1.zip › Western Blot/CREB-3.tif]

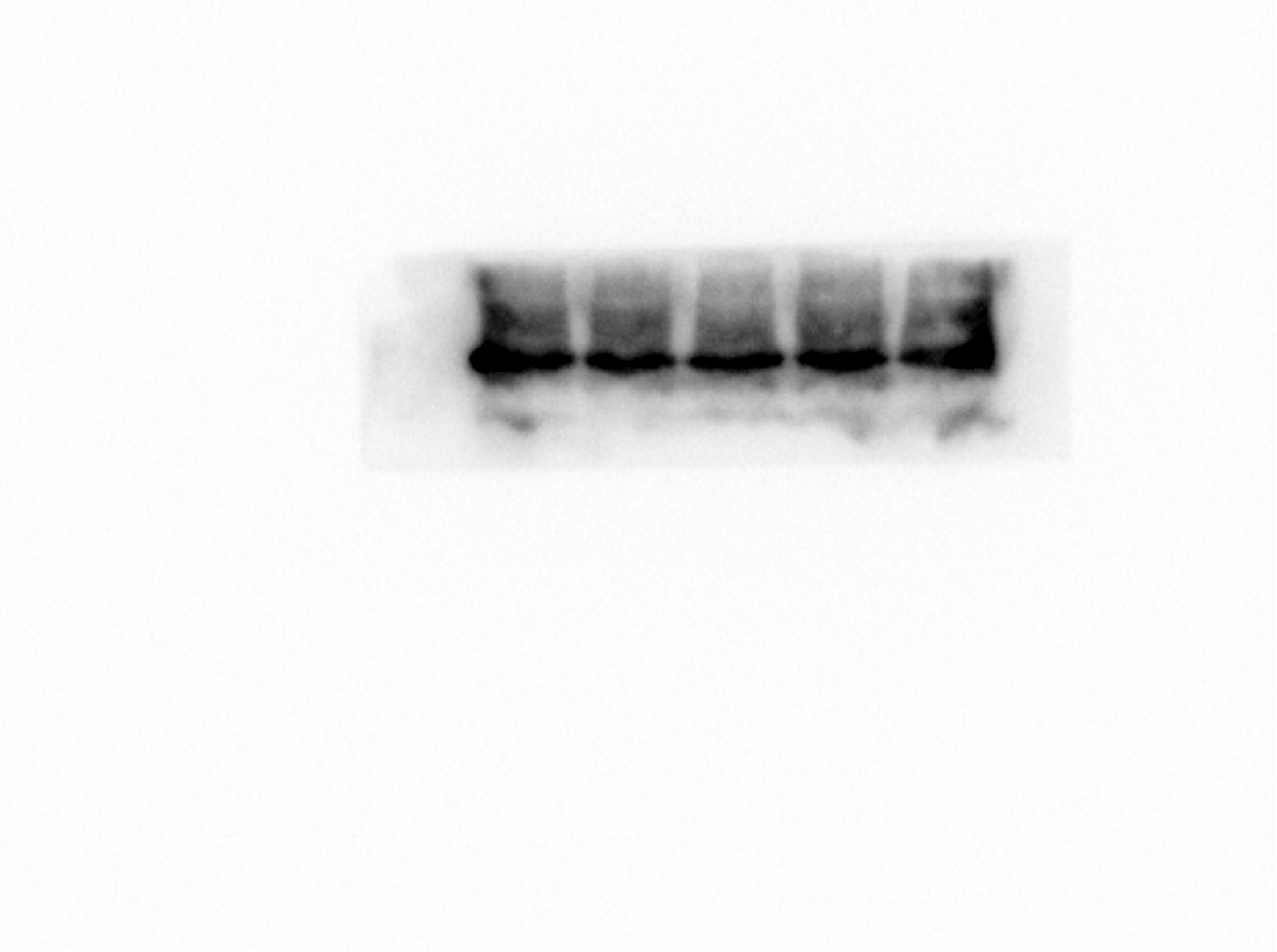

Supplement: Supplementary file 1 [file Presentation1.zip › Western Blot/CREB.tif]

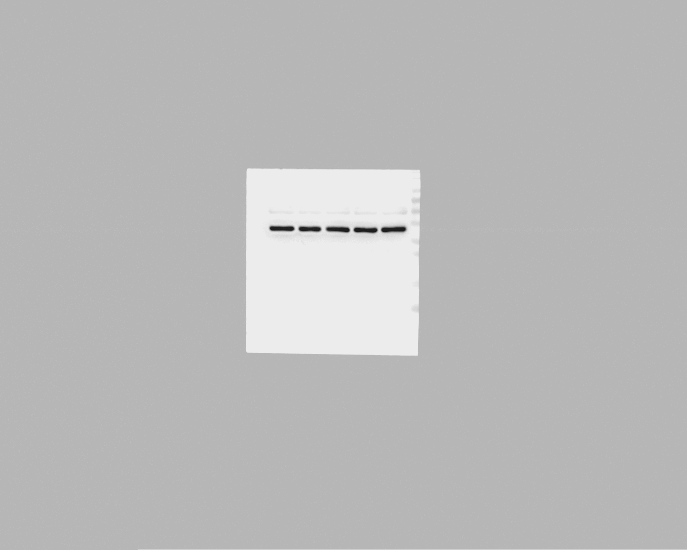

Supplement: Supplementary file 1 [file Presentation1.zip › Western Blot/GAPDH-2.tif]

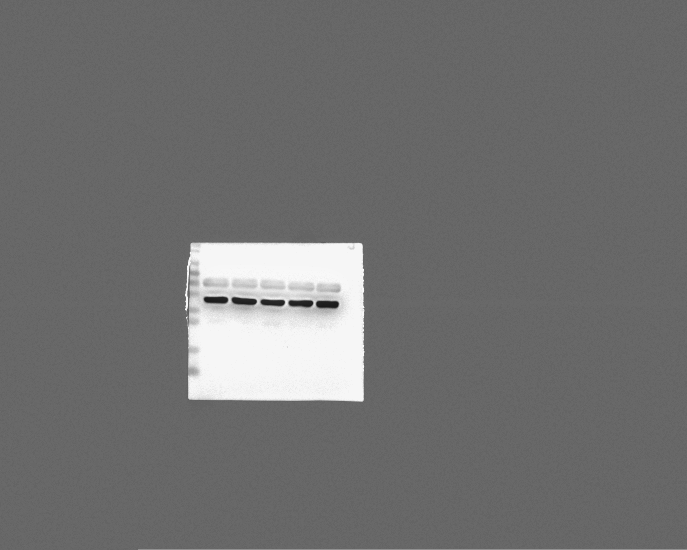

Supplement: Supplementary file 1 [file Presentation1.zip › Western Blot/GAPDH-3.tif]

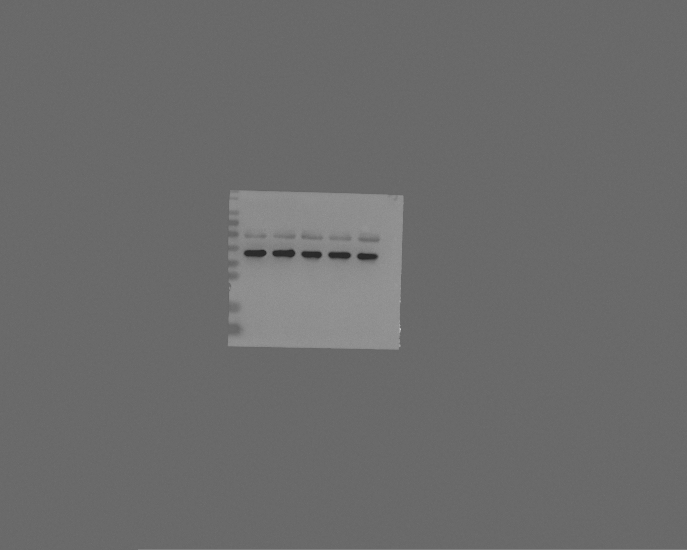

Supplement: Supplementary file 1 [file Presentation1.zip › Western Blot/GAPDH-4.tif]

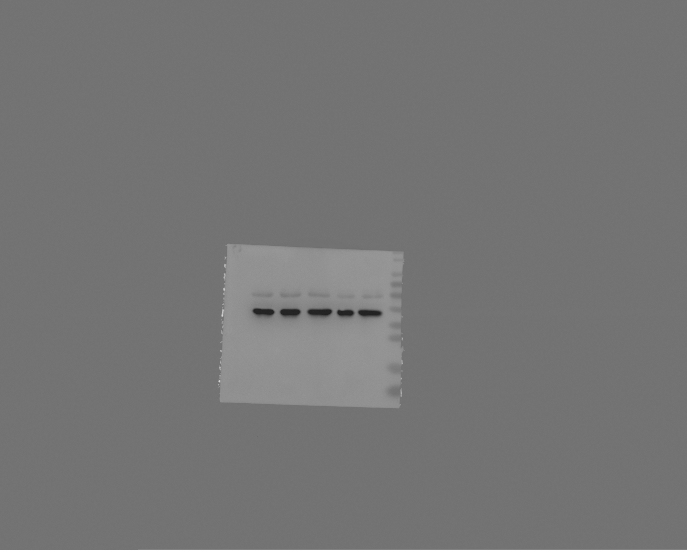

Supplement: Supplementary file 1 [file Presentation1.zip › Western Blot/GAPDH-5.tif]

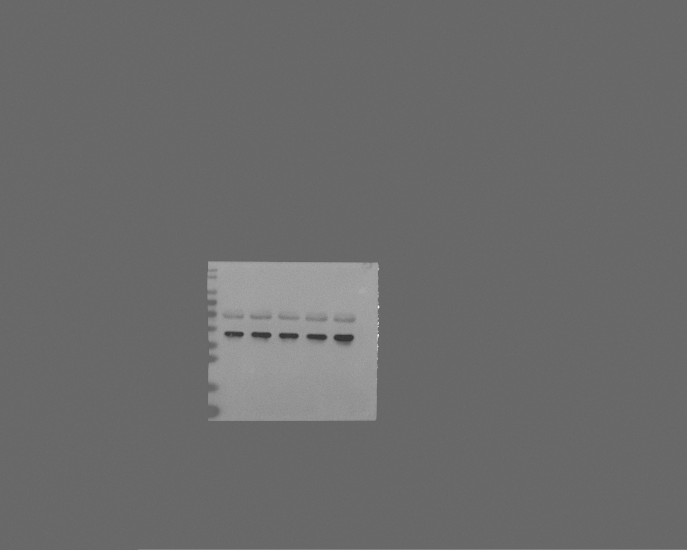

Supplement: Supplementary file 1 [file Presentation1.zip › Western Blot/GAPDH-6.tif]

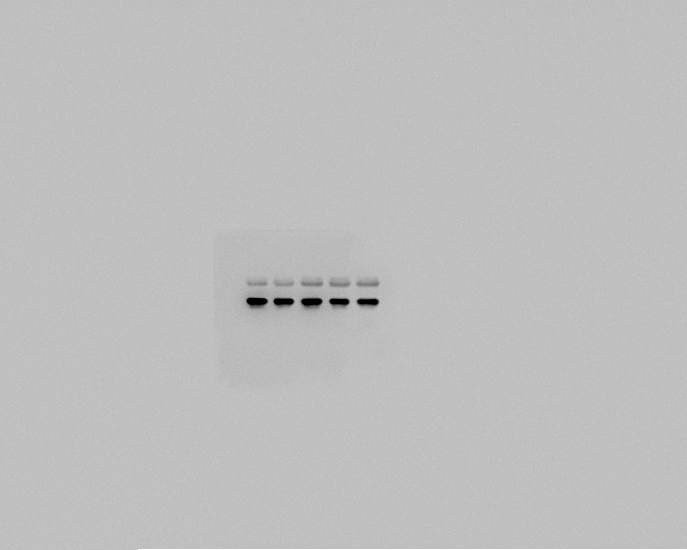

Supplement: Supplementary file 1 [file Presentation1.zip › Western Blot/GAPDH-7.tif]

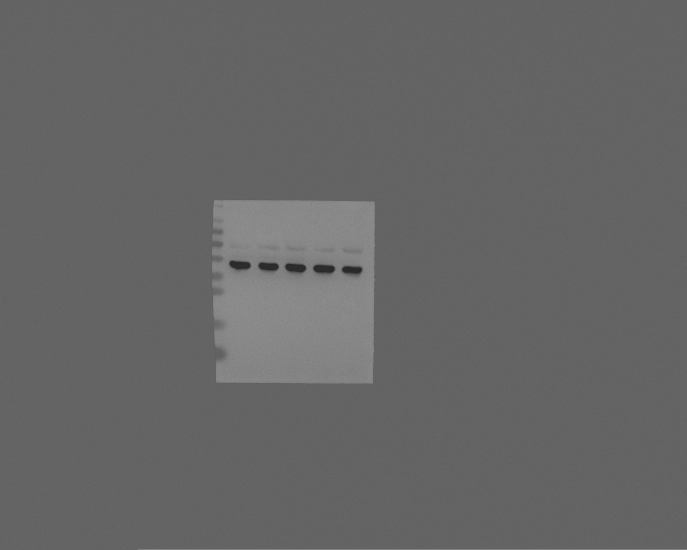

Supplement: Supplementary file 1 [file Presentation1.zip › Western Blot/GAPDH-8.tif]

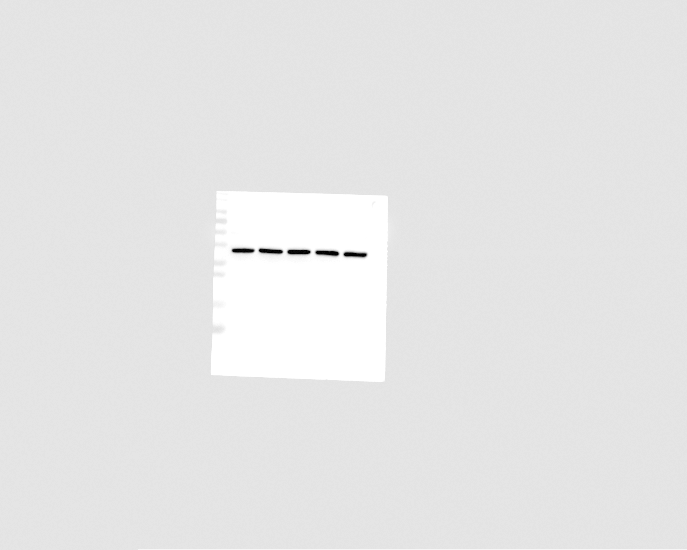

Supplement: Supplementary file 1 [file Presentation1.zip › Western Blot/GAPDH.tif]

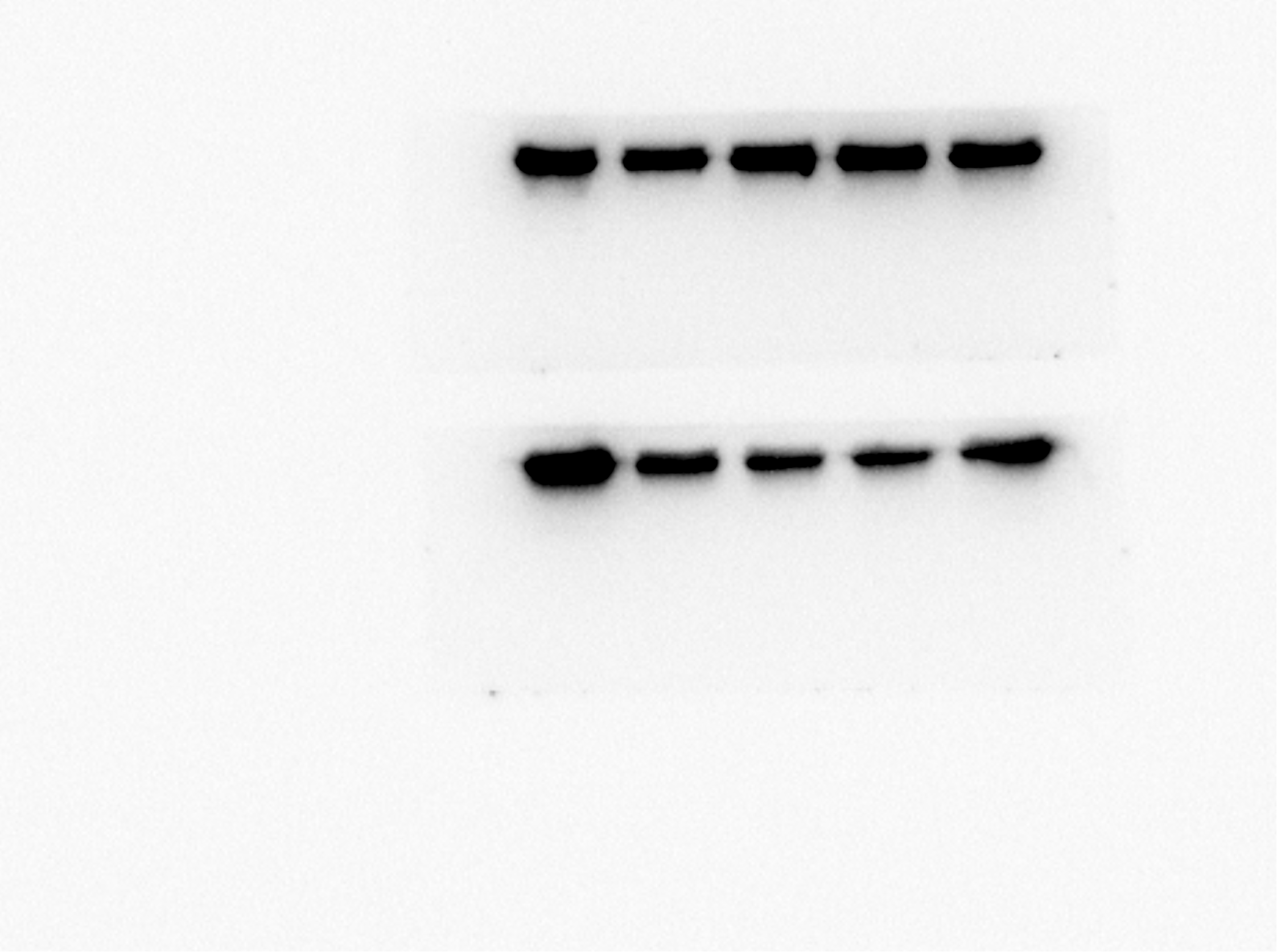

Supplement: Supplementary file 1 [file Presentation1.zip › Western Blot/GSK3β-2.tif]

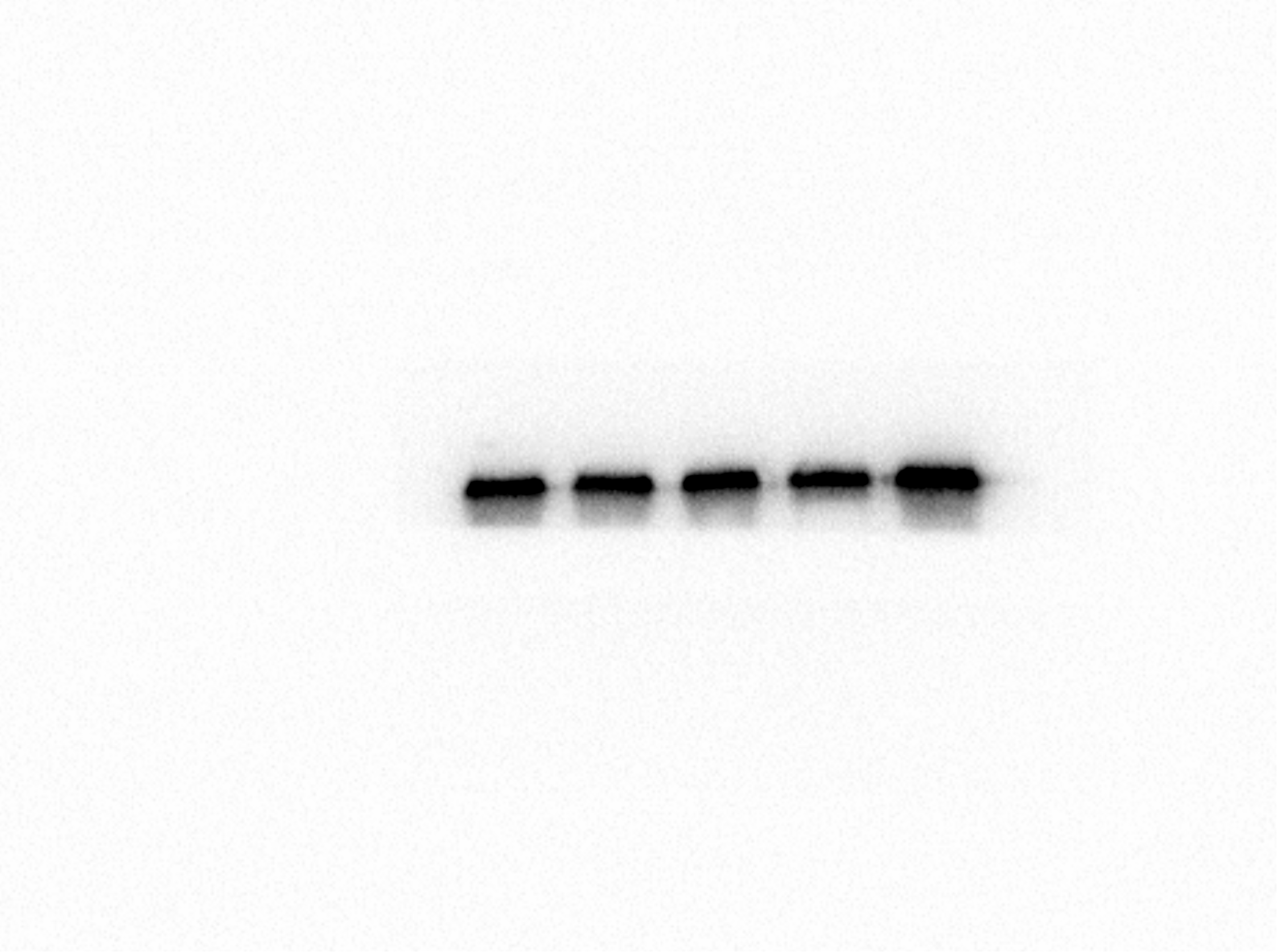

Supplement: Supplementary file 1 [file Presentation1.zip › Western Blot/GSK3β-3.tif]

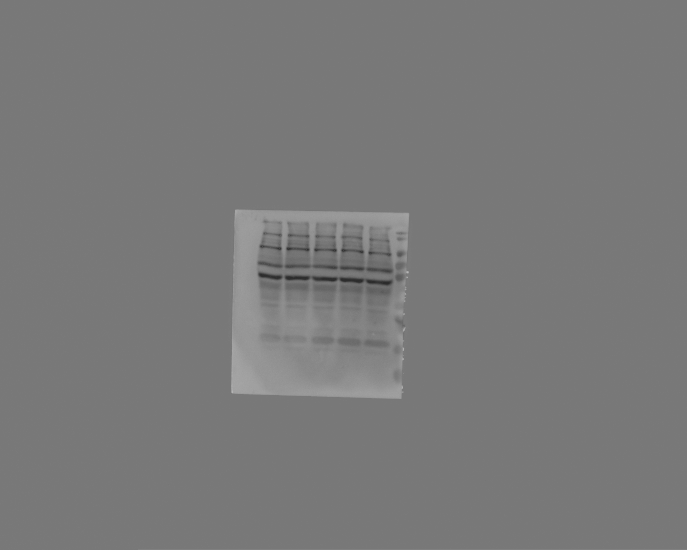

Supplement: Supplementary file 1 [file Presentation1.zip › Western Blot/GSK3β.tif]

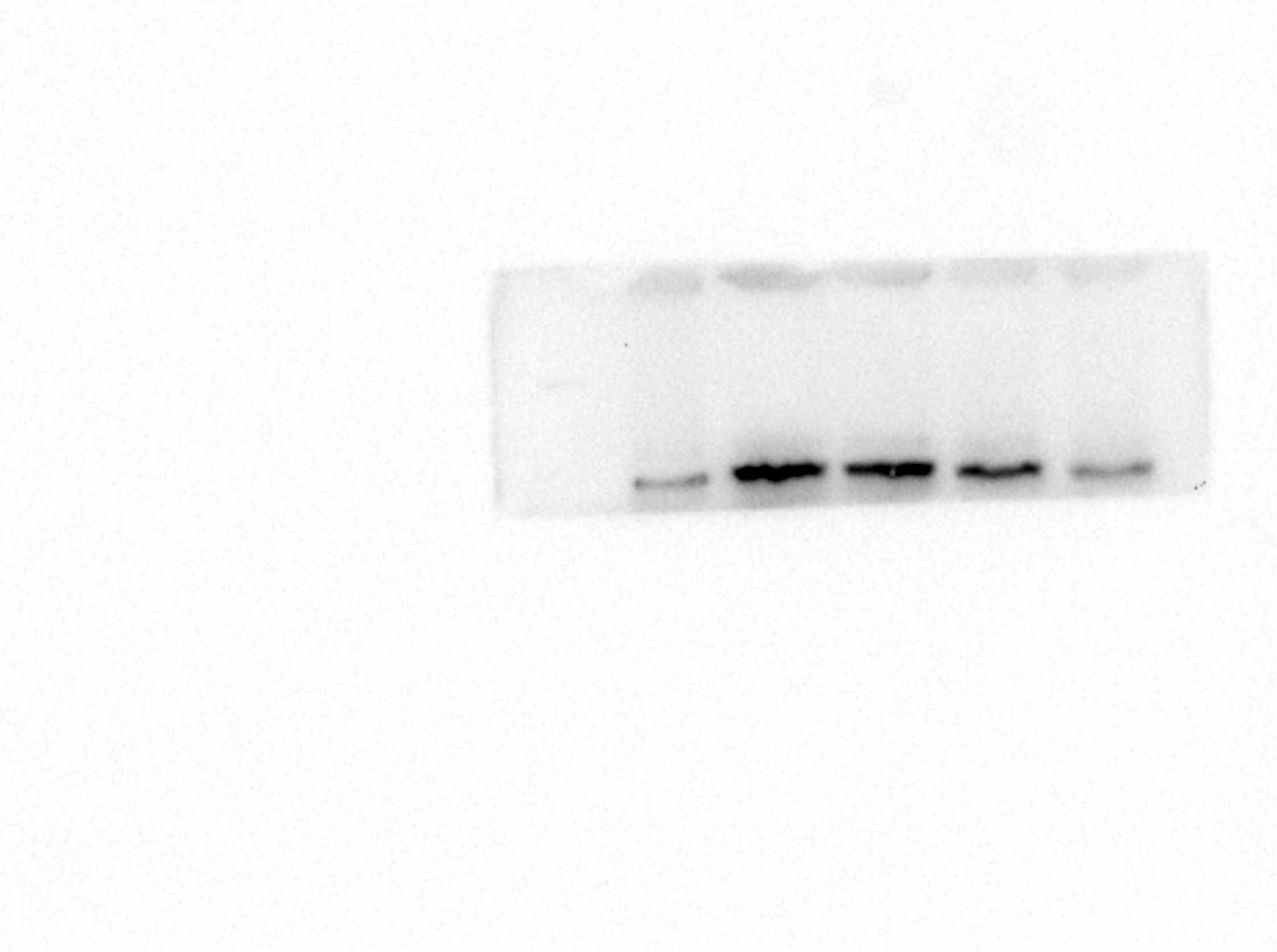

Supplement: Supplementary file 1 [file Presentation1.zip › Western Blot/IL-18-2.tif]

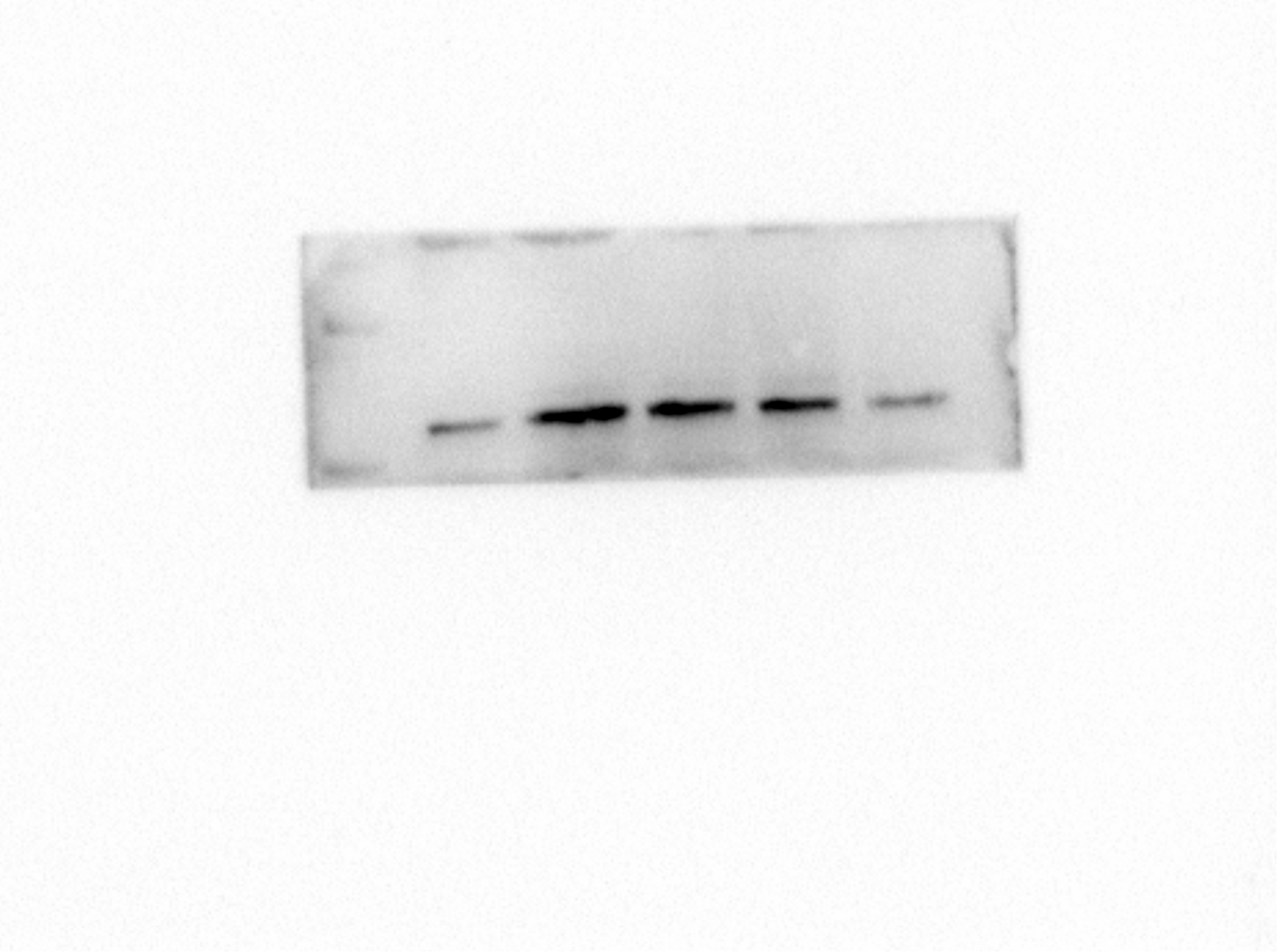

Supplement: Supplementary file 1 [file Presentation1.zip › Western Blot/IL-18-3.tif]

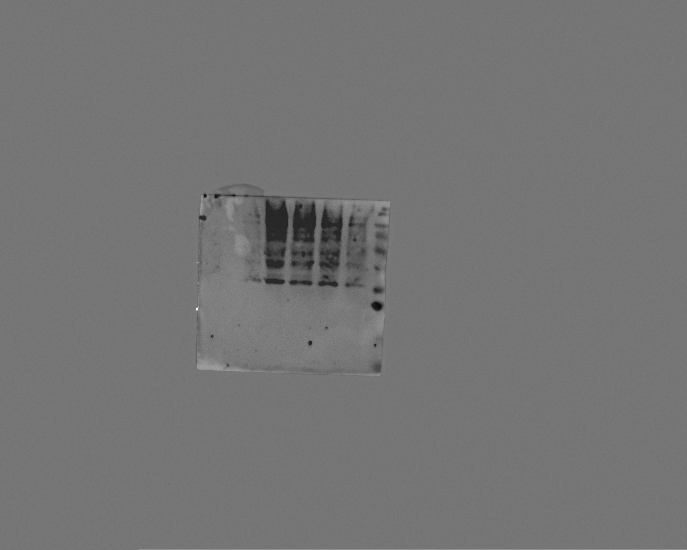

Supplement: Supplementary file 1 [file Presentation1.zip › Western Blot/IL-18.tif]

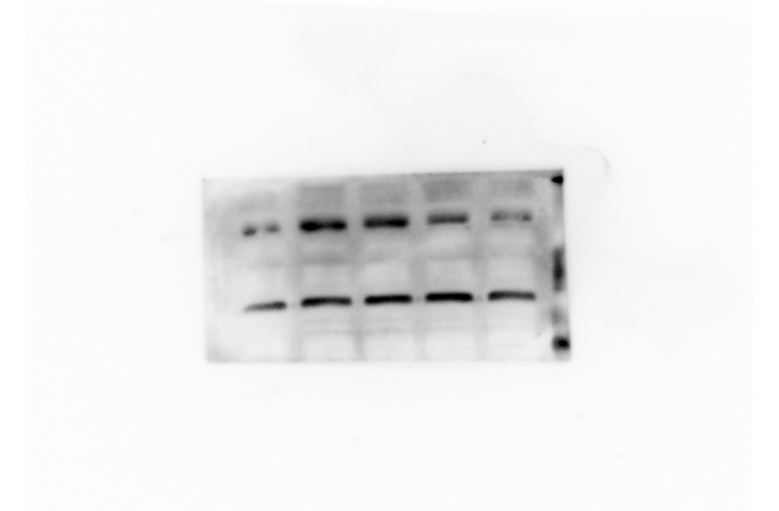

Supplement: Supplementary file 1 [file Presentation1.zip › Western Blot/IL-1β-2.tif]

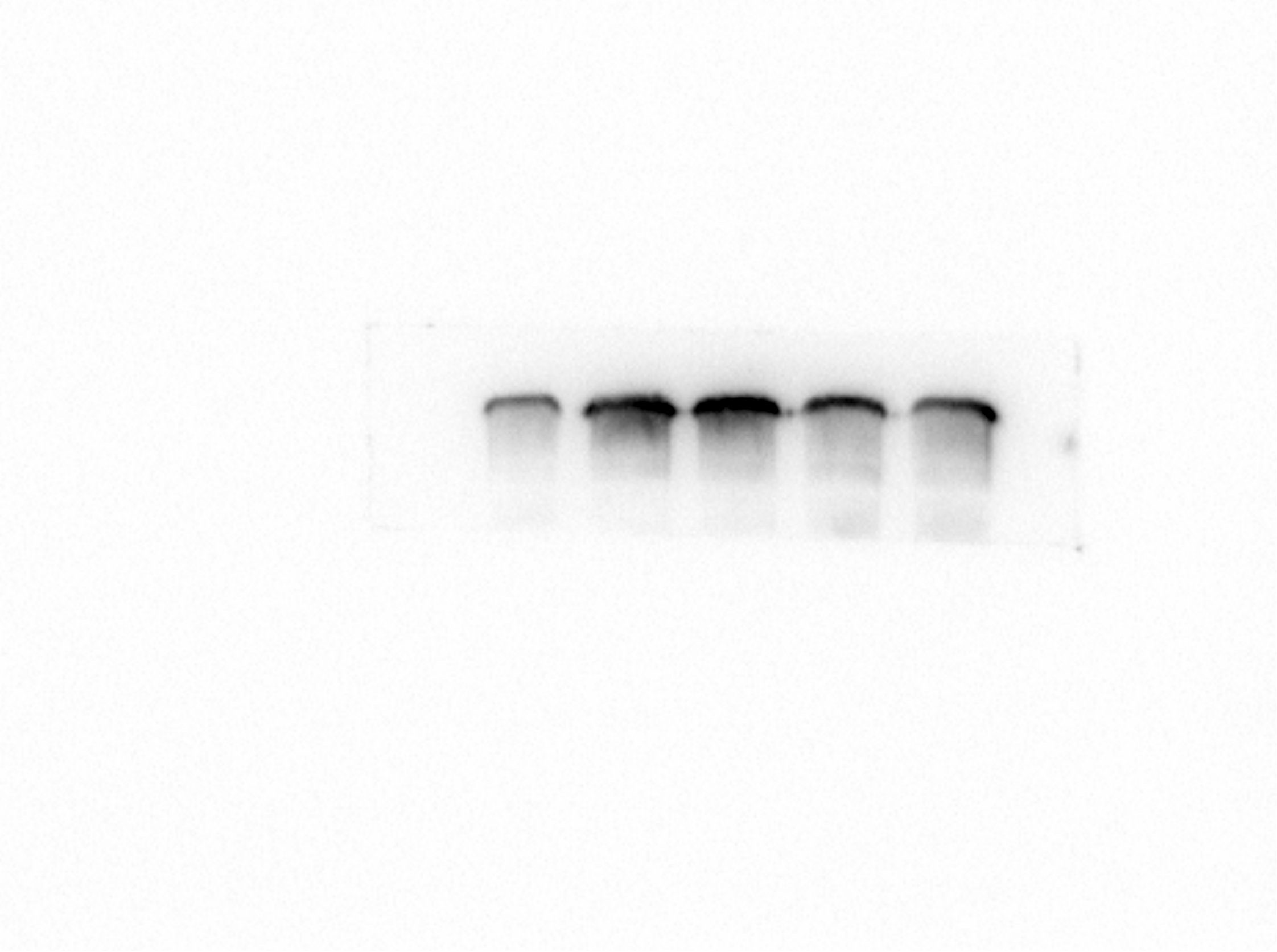

Supplement: Supplementary file 1 [file Presentation1.zip › Western Blot/IL-1β-3.tif]

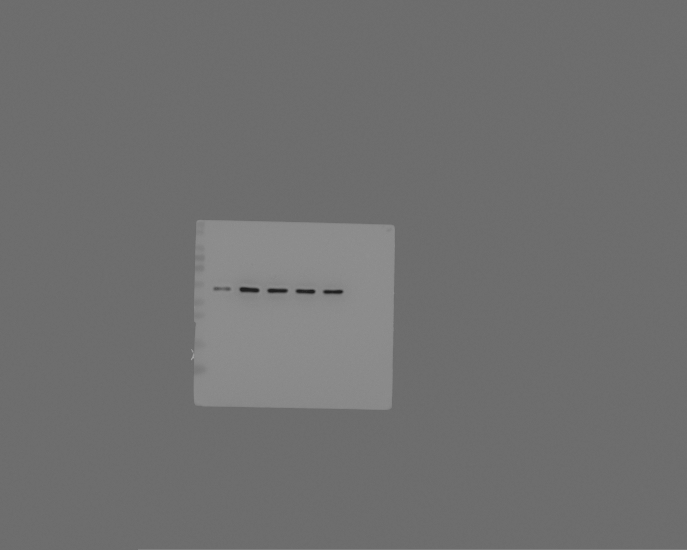

Supplement: Supplementary file 1 [file Presentation1.zip › Western Blot/IL-1β.tif]

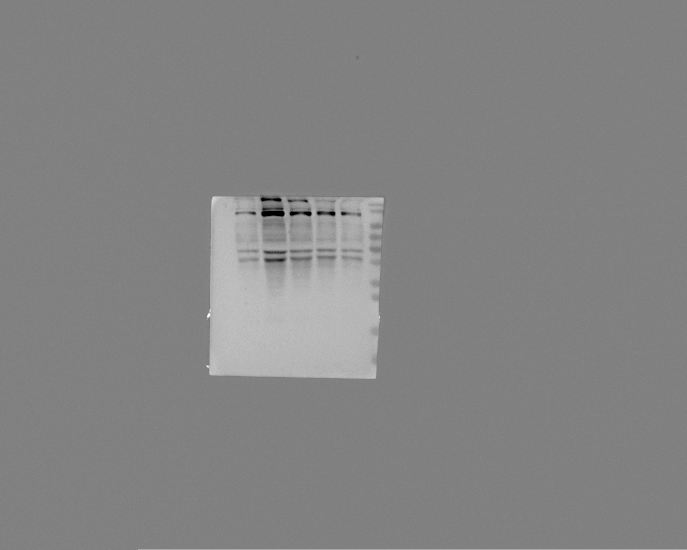

Supplement: Supplementary file 1 [file Presentation1.zip › Western Blot/NLRP3-2.tif]

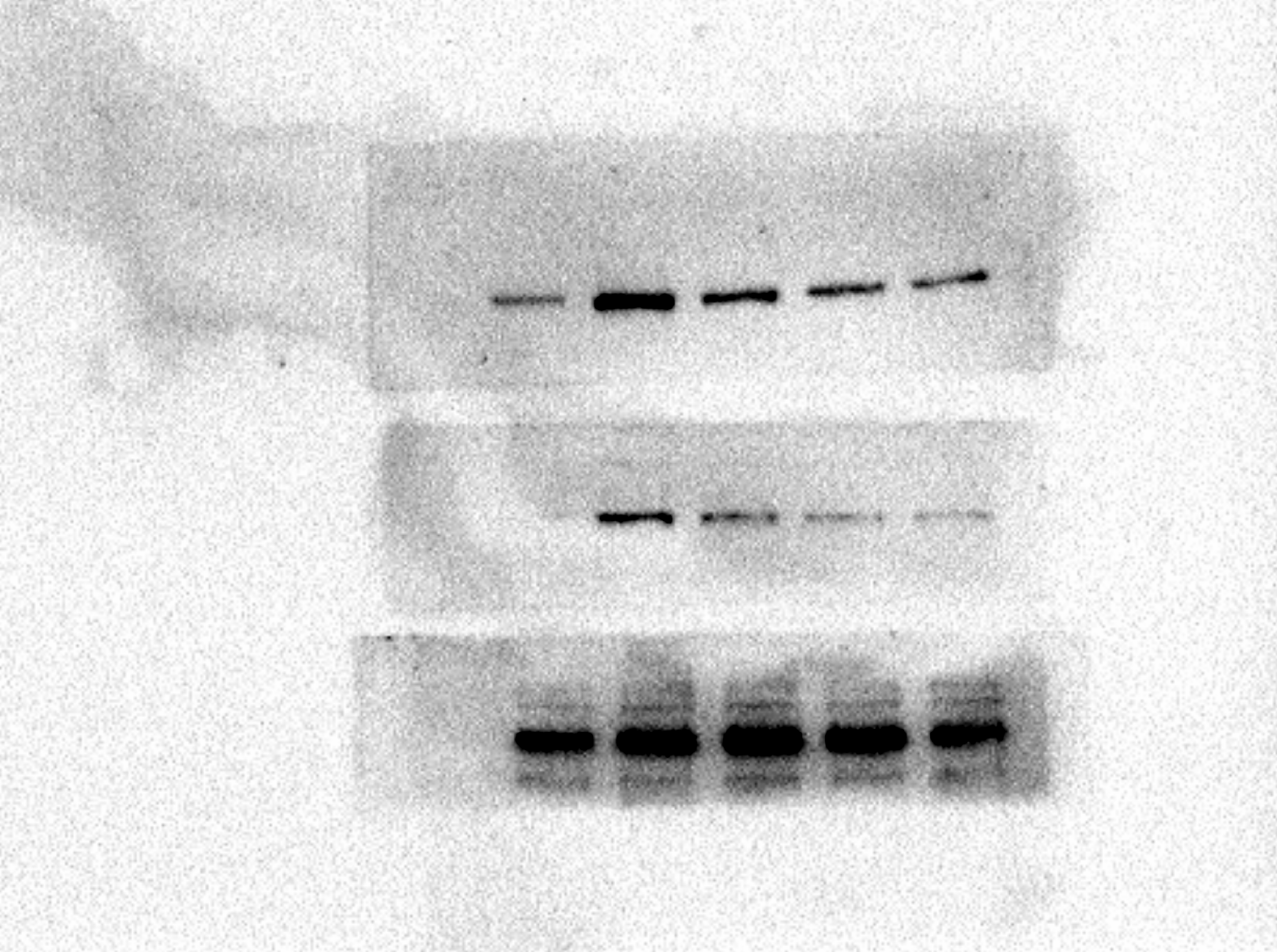

Supplement: Supplementary file 1 [file Presentation1.zip › Western Blot/NLRP3-3.tif]

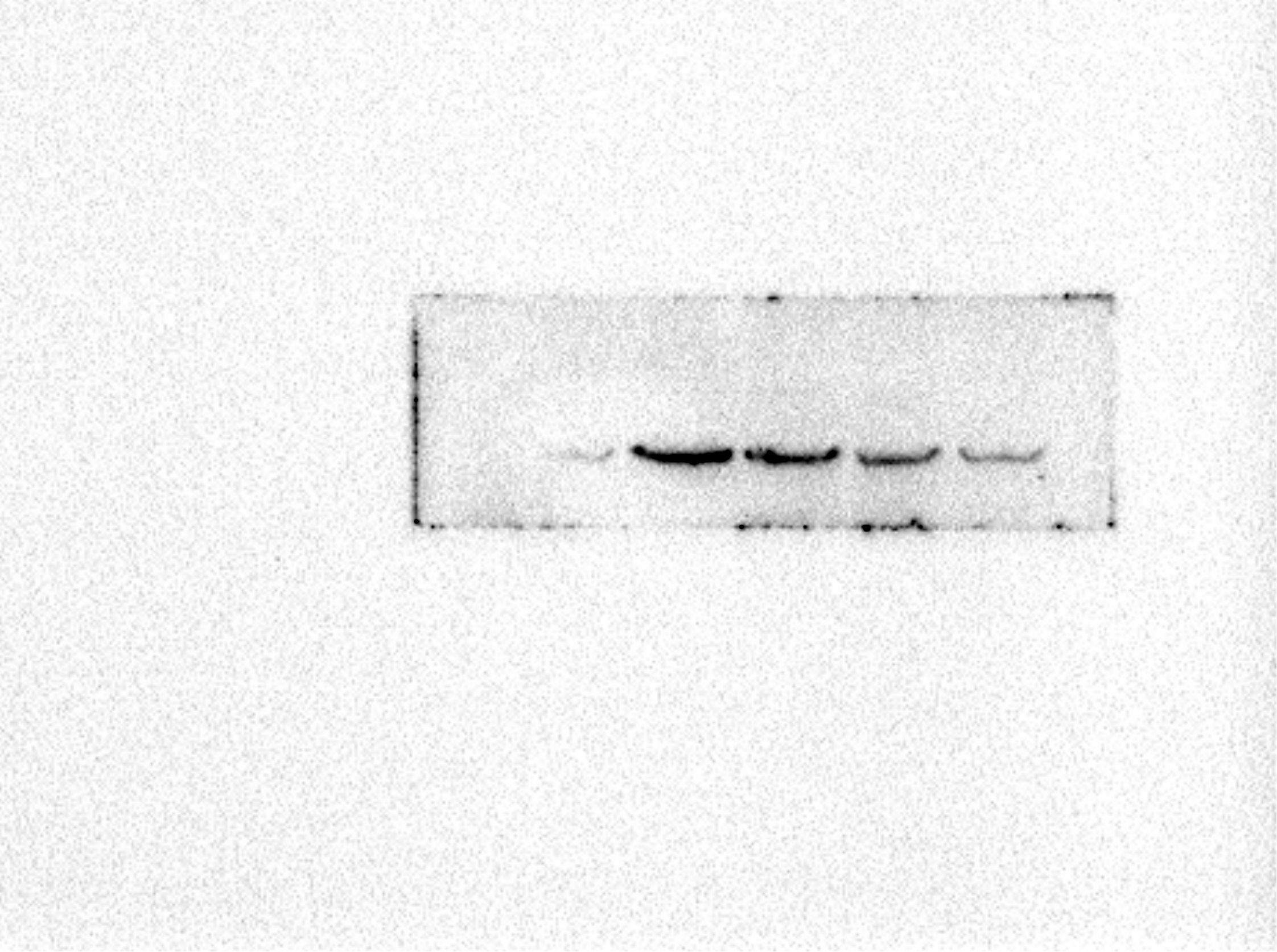

Supplement: Supplementary file 1 [file Presentation1.zip › Western Blot/NLRP3.tif]

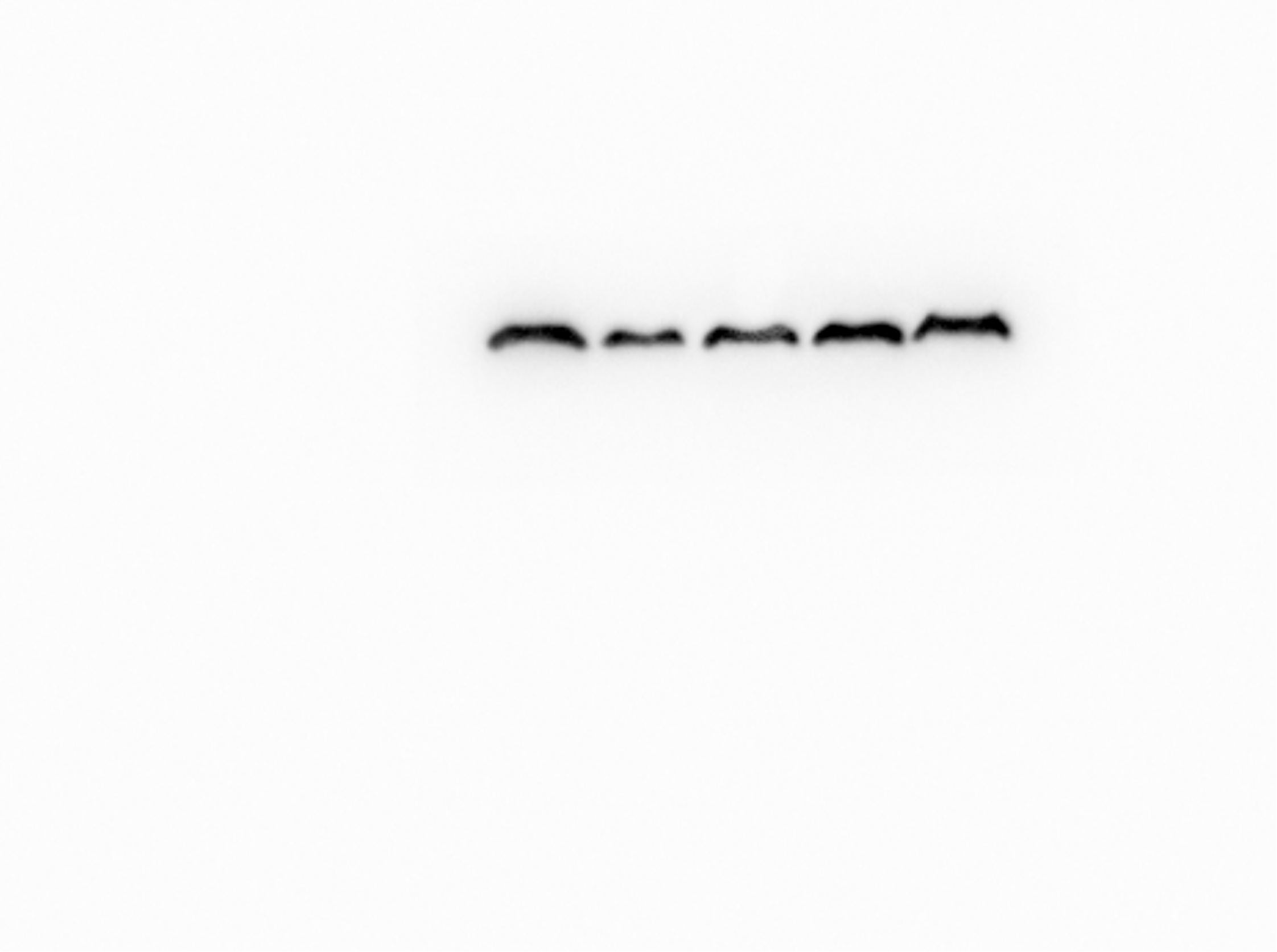

Supplement: Supplementary file 1 [file Presentation1.zip › Western Blot/p-AKT-2.tif]

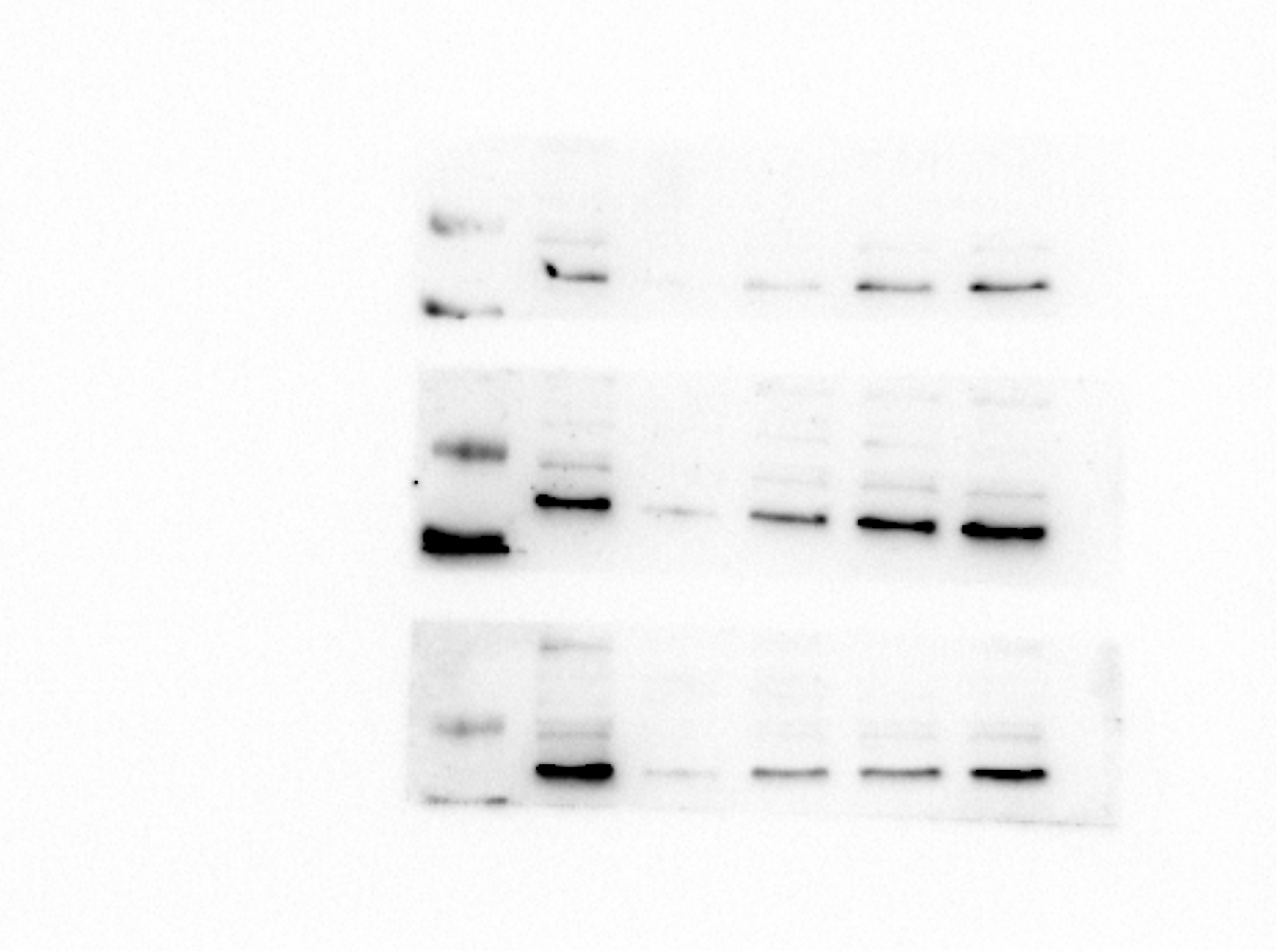

Supplement: Supplementary file 1 [file Presentation1.zip › Western Blot/p-AKT-3.tif]

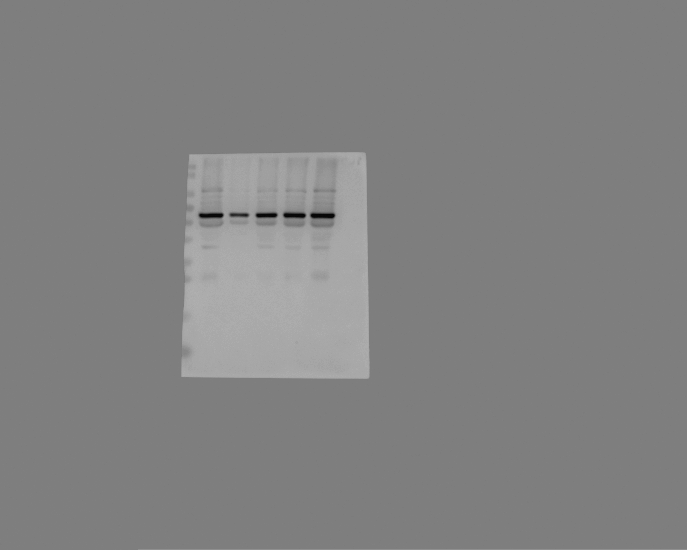

Supplement: Supplementary file 1 [file Presentation1.zip › Western Blot/p-AKT.tif]

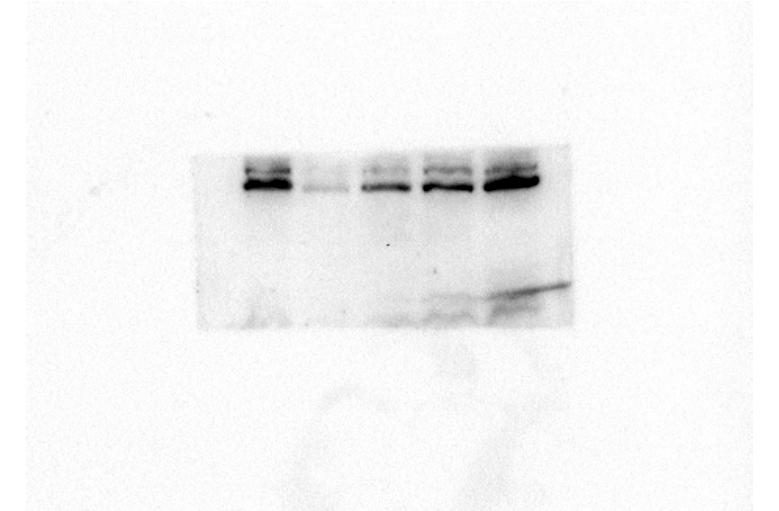

Supplement: Supplementary file 1 [file Presentation1.zip › Western Blot/p-CREB-2.tif]

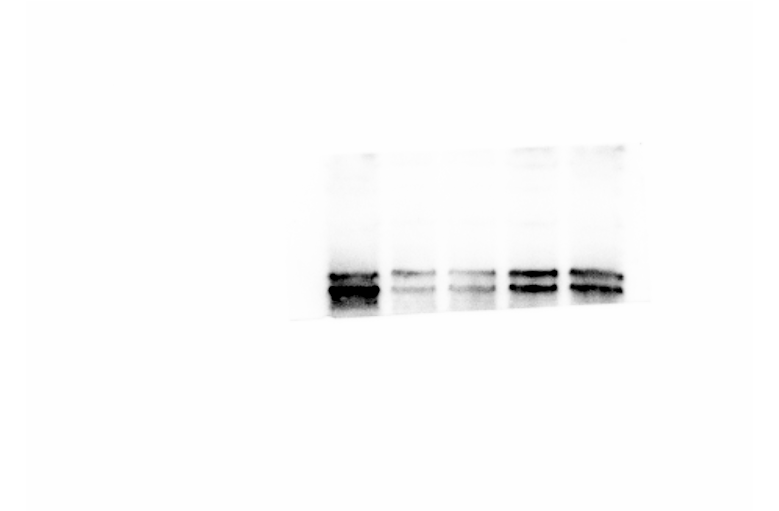

Supplement: Supplementary file 1 [file Presentation1.zip › Western Blot/p-CREB-3.tif]

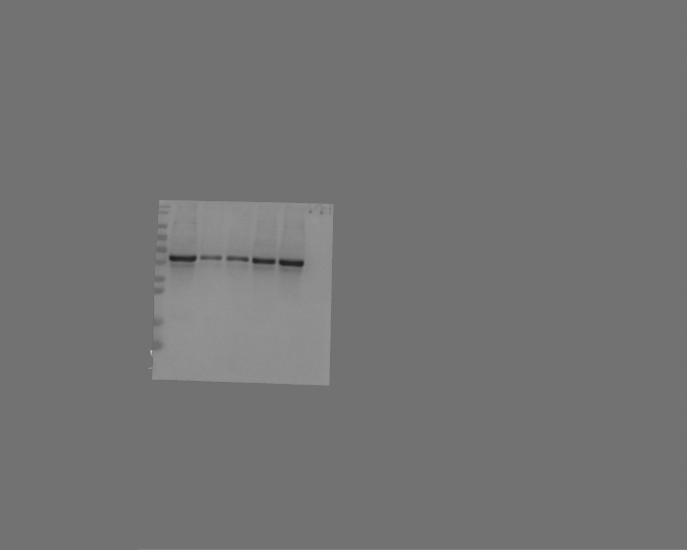

Supplement: Supplementary file 1 [file Presentation1.zip › Western Blot/p-CREB.tif]

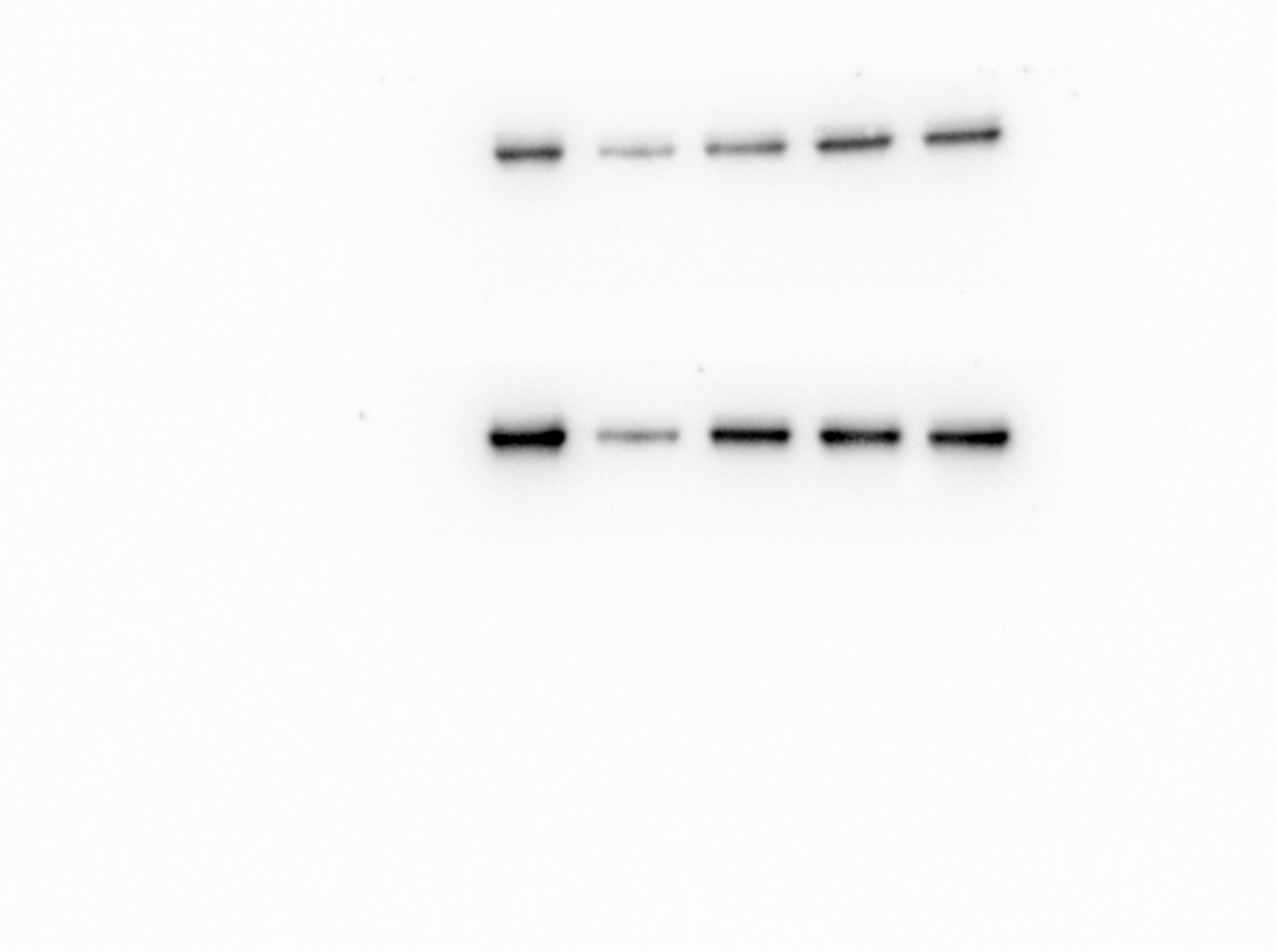

Supplement: Supplementary file 1 [file Presentation1.zip › Western Blot/p-GSK3β-2.tif]

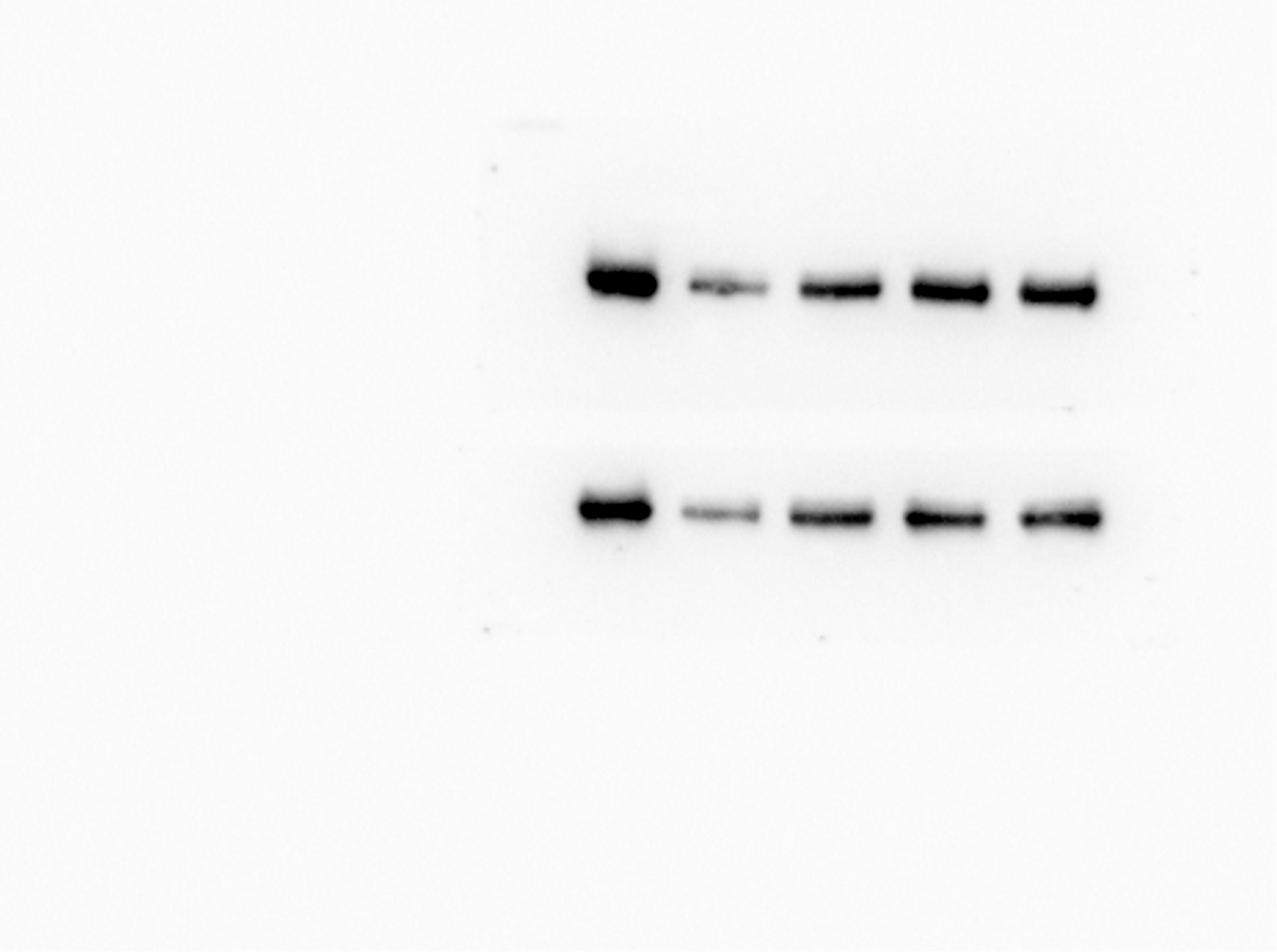

Supplement: Supplementary file 1 [file Presentation1.zip › Western Blot/p-GSK3β-3.tif]

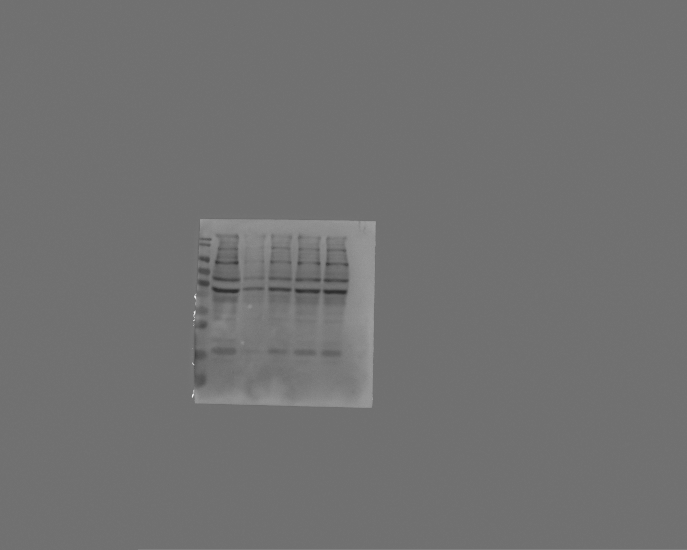

Supplement: Supplementary file 1 [file Presentation1.zip › Western Blot/p-GSK3β.tif]

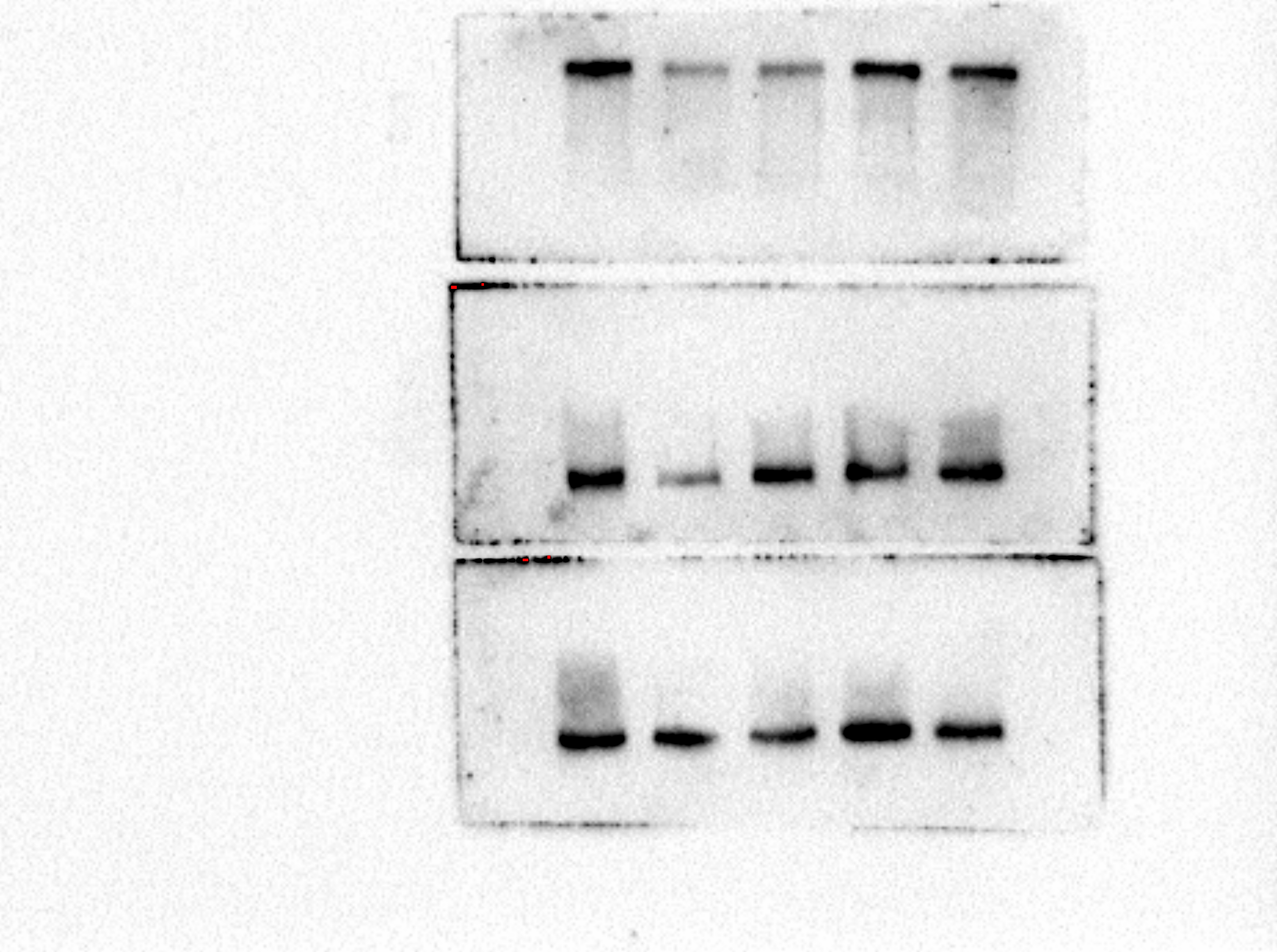

Supplement: Supplementary file 1 [file Presentation1.zip › Western Blot/p-PI3K-2.tif]

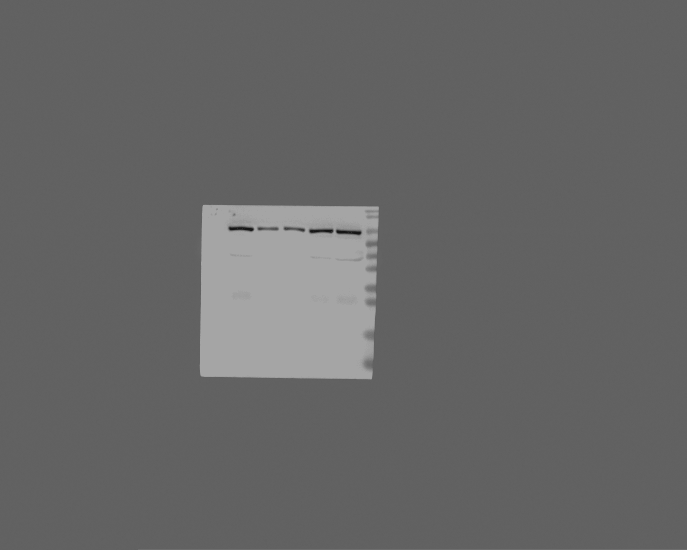

Supplement: Supplementary file 1 [file Presentation1.zip › Western Blot/p-PI3K.tif]

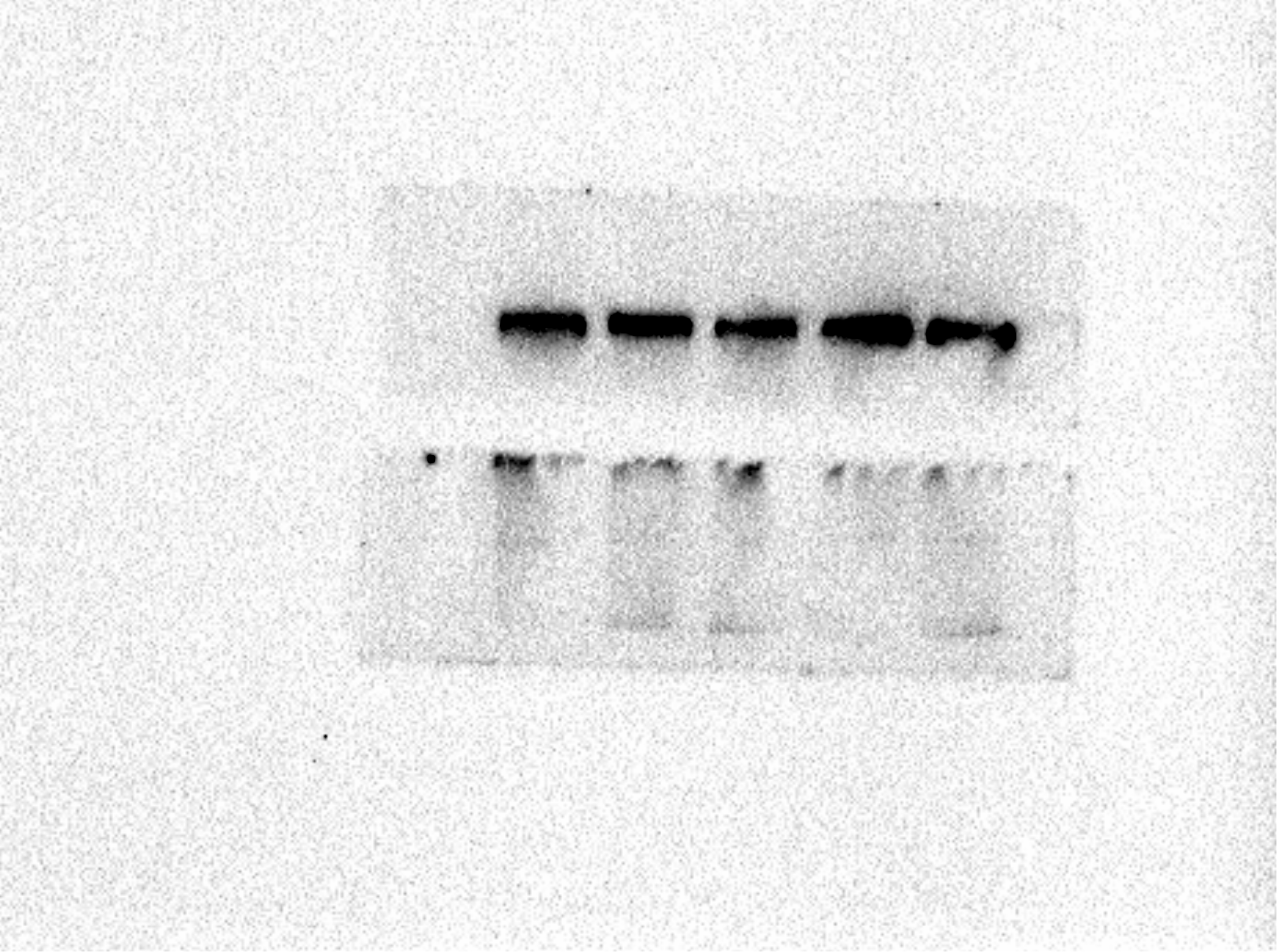

Supplement: Supplementary file 1 [file Presentation1.zip › Western Blot/PI3K-2.tif]

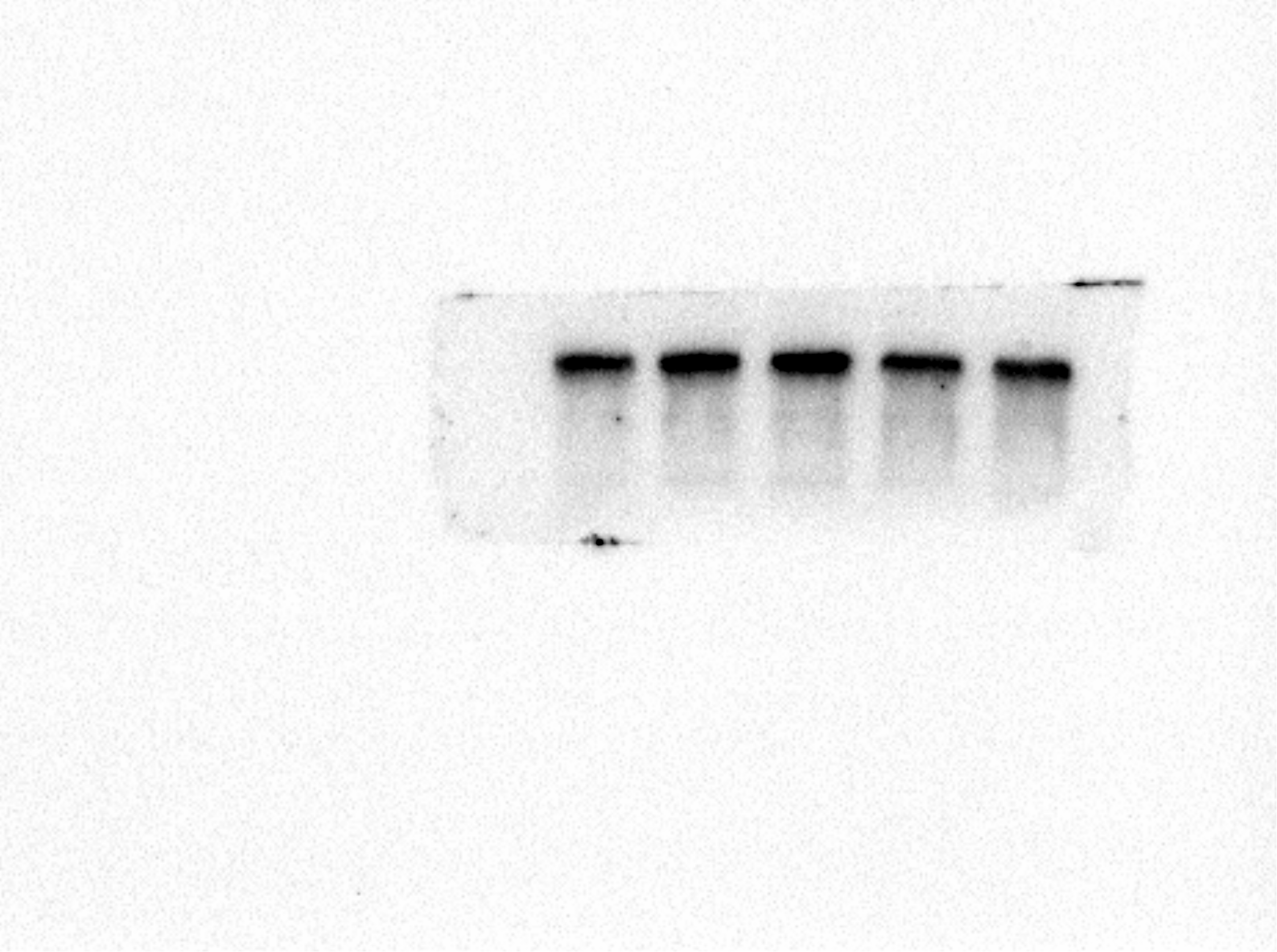

Supplement: Supplementary file 1 [file Presentation1.zip › Western Blot/PI3K-3.tif]

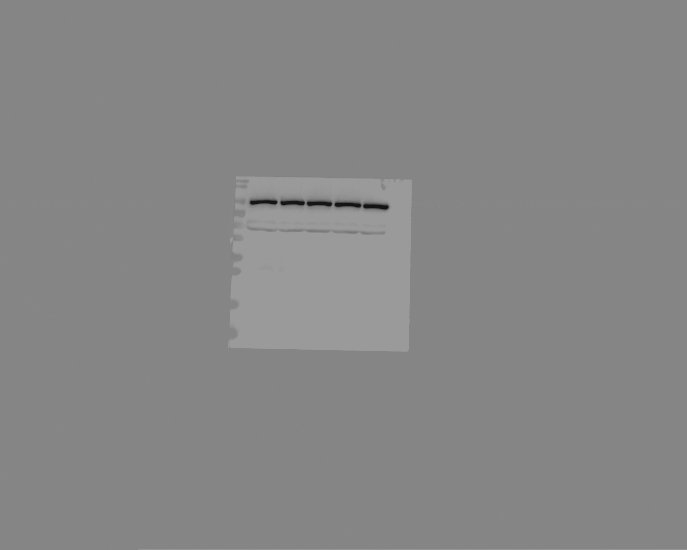

Supplement: Supplementary file 1 [file Presentation1.zip › Western Blot/PI3K.tif]

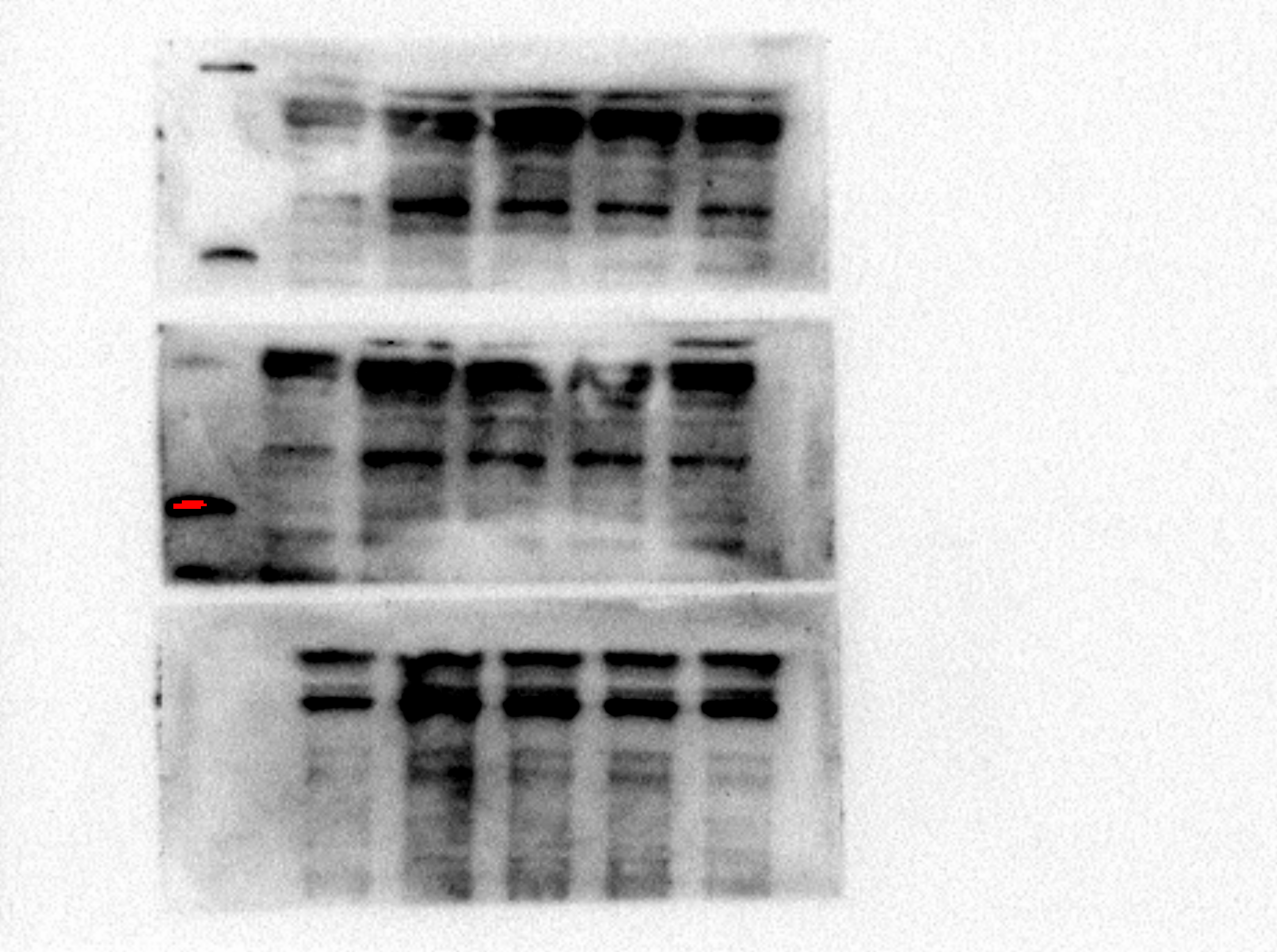

Supplement: Supplementary file 1 [file Presentation1.zip › Western Blot/TNF-α-2.tif]

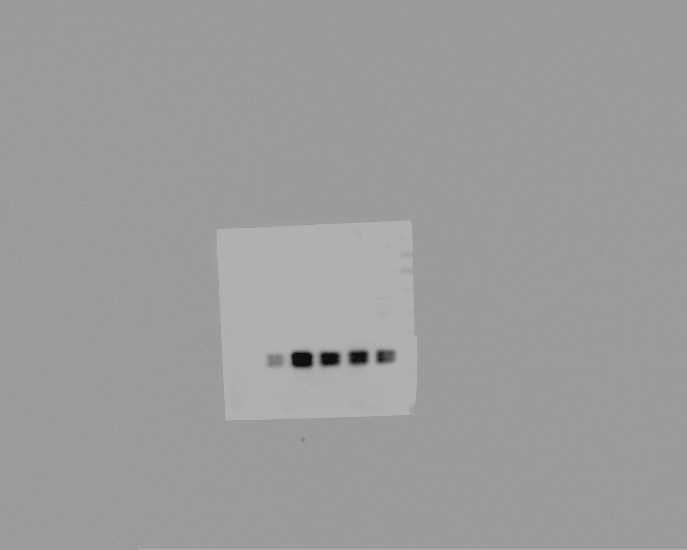

Supplement: Supplementary file 1 [file Presentation1.zip › Western Blot/TNF-α.tif]

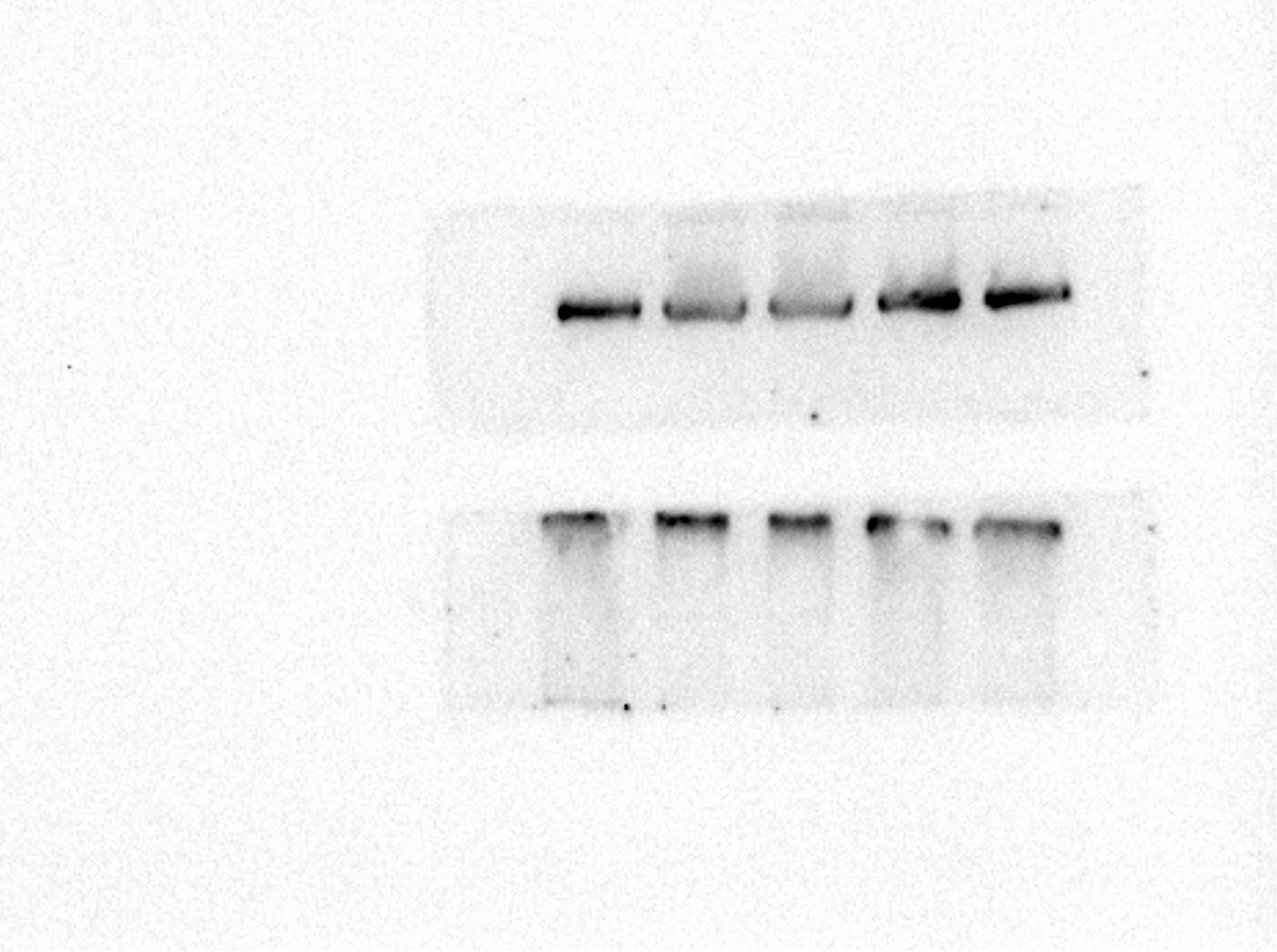

Supplement: Supplementary file 1 [file Presentation1.zip › Western Blot/TrkB-2.tif]

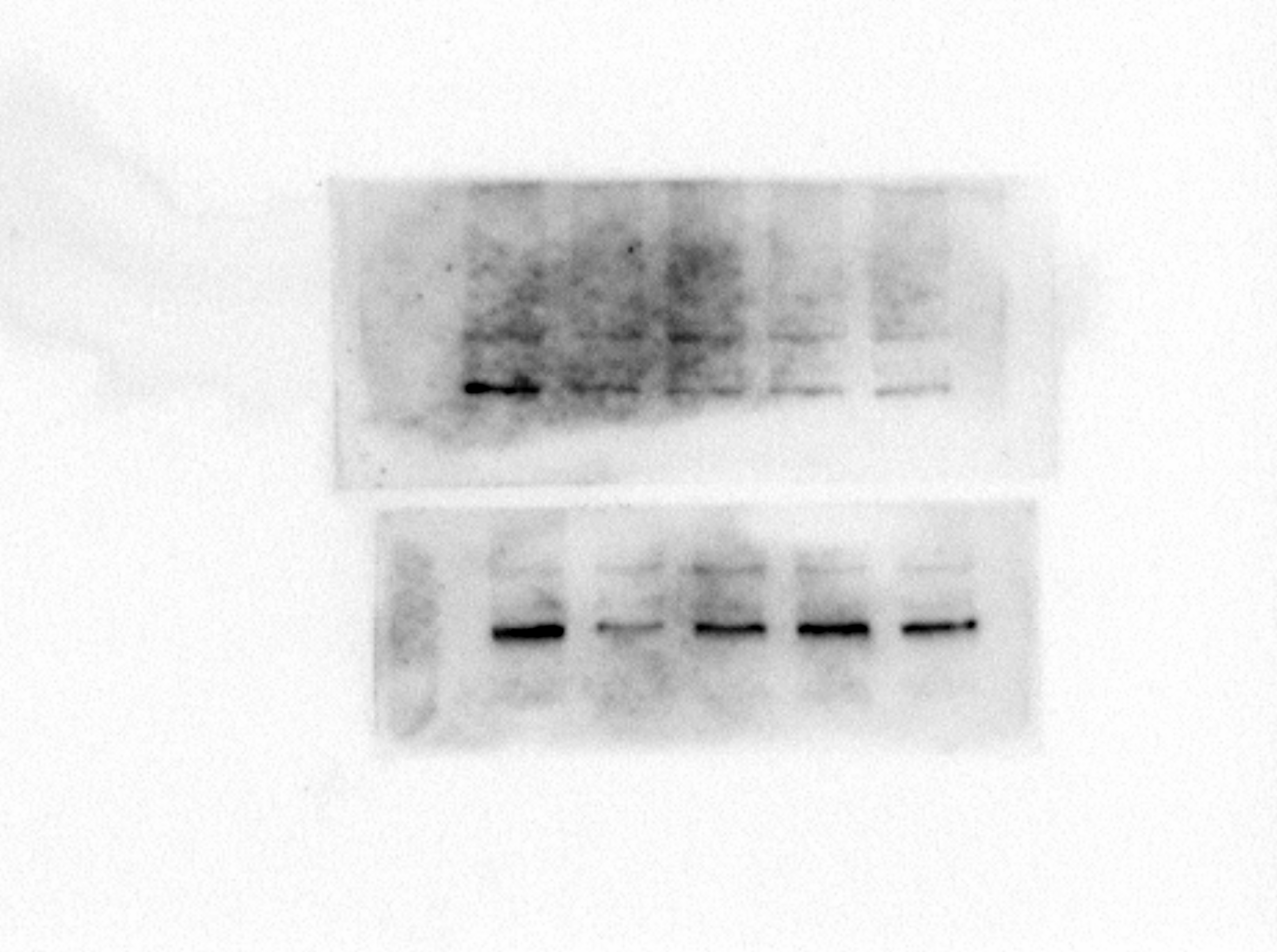

Supplement: Supplementary file 1 [file Presentation1.zip › Western Blot/TrkB-3.tif]

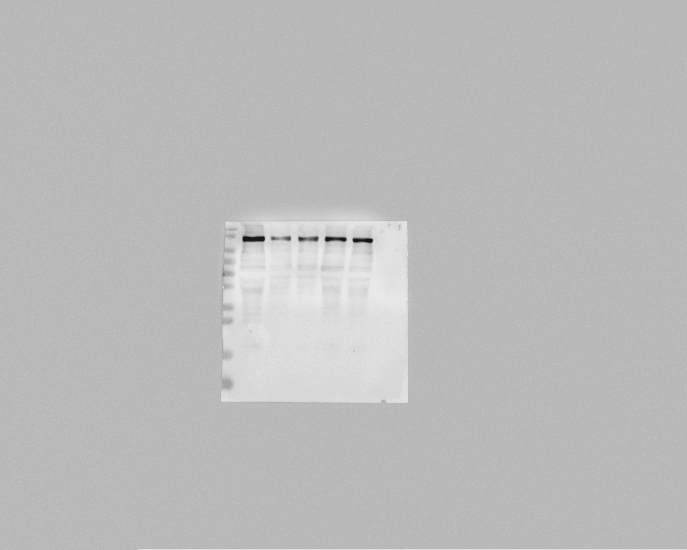

Supplement: Supplementary file 1 [file Presentation1.zip › Western Blot/TrkB.tif]
